# Understanding the causes of missingness in primary care: a realist review

**DOI:** 10.1186/s12916-024-03456-2

**Published:** 2024-06-10

**Authors:** Calum Lindsay, David Baruffati, Mhairi Mackenzie, David A. Ellis, Michelle Major, Catherine A. O’Donnell, Sharon A. Simpson, Andrea E. Williamson, Geoff Wong

**Affiliations:** 1 General Practice and Primary Care, School of Health and Wellbeing, University of Glasgow, Clarice Pears Building, 90 Byres Road, Glasgow, G12 8TB UK; 2 School of Social & Political Sciences, Urban Studies, University of Glasgow, 27 Bute Gardens, Glasgow, G12 8RS UK; 3 Centre for Healthcare Innovation and Improvement Information, Decisions and Operations, Centre for Business Organisations and Society (CBOS), University of Bath, Bath, UK; 4 Homeless Network Scotland, 12 Commercial Rd, Adelphi Centre, Gorbals, Glasgow G5 0PQ UK; 5 MRC/CSO Social & Public Health Sciences Unit, School of Health and Wellbeing, University of Glasgow, Glasgow, UK; 6 Nuffield Department of Primary Care Health Sciences, University of Oxford, Oxford, UK

**Keywords:** Multiple missed appointments, Health service access, Health service equity, Candidacy, Primary care, Realist synthesis

## Abstract

**Background:**

Although missed appointments in healthcare have been an area of concern for policy, practice and research, the primary focus has been on reducing single ‘situational’ missed appointments to the benefit of services. Little attention has been paid to the causes and consequences of more ‘enduring’ multiple missed appointments in primary care and the role this has in producing health inequalities.

**Methods:**

We conducted a realist review of the literature on multiple missed appointments to identify the causes of ‘missingness.’ We searched multiple databases, carried out iterative citation-tracking on key papers on the topic of missed appointments and identified papers through searches of grey literature. We synthesised evidence from 197 papers, drawing on the theoretical frameworks of candidacy and fundamental causation.

**Results:**

Missingness is caused by an overlapping set of complex factors, including patients not identifying a need for an appointment or feeling it is ‘for them’; appointments as sites of poor communication, power imbalance and relational threat; patients being exposed to competing demands, priorities and urgencies; issues of travel and mobility; and an absence of choice or flexibility in when, where and with whom appointments take place.

**Conclusions:**

Interventions to address missingness at policy and practice levels should be theoretically informed, tailored to patients experiencing missingness and their identified needs and barriers; be cognisant of causal domains at multiple levels and address as many as practical; and be designed to increase safety for those seeking care.

**Supplementary Information:**

The online version contains supplementary material available at 10.1186/s12916-024-03456-2.

## Background

Non-attendance at health appointments has garnered significant policy attention in high income countries, often framed in terms of waste, inefficiency, value-for money, long waiting lists or reduced capacity within services struggling to meet demand [1–4]. The existing evidence base around non-attendance has several strands. Some studies identify patient- or service-side variables associated with non-attendance, and increasingly their findings are used to build machine-learning algorithms to predict, and schedule around, non-attendance [5–7]. Other research uses surveys, questionnaires and interviews with staff and patients to build a picture of why appointments are missed. Intervention studies build upon these to test strategies for reducing non-attendance, typically measuring percentage reductions across the general patient population or among all patients missing appointments. These existing approaches rarely distinguish patients missing single appointments—potentially a *situational* issue—from patients who miss multiple appointments, for whom barriers to care may be more *enduring.* They also retain a primary focus on the impacts of non-attendance for health services rather than patients, with interventions oriented towards what provides those services with greatest benefit.

Multiple missed appointments have received occasional attention within this research base, with a variety of definitions. Some take absolute numbers: Traeger et al. [8] differentiate those missing 3 appointments (3.4% of patients) from those missing 5 or more (3.8%); Dumontier et al. [9] focusing on the 2% of patients who missed 6 or more appointments over 18 months; Cashman et al. [10] the 30% of patients missing 3 or more appointments over 18 months; or Chapman et al. [11] the patients missing 4 or more appointments over a 12-month period. Others look in relative terms, whether at missed appointments as a proportion of a patient’s total appointments—in Shimotsu et al. [12] 20% of patients miss more than 30% of their appointments, while 9% of patients miss more than one third of their follow-up appointments in Parker et al. [13]—or relative to other patients, as in Samuels et al. [14] who focus on patients above the 90% percentile in missed appointment rate. This paper follows a large-scale epidemiological project aimed at addressing knowledge gaps around multiple missed appointments in UK primary care. The project differed from many prior papers by directly linking multiple missed appointments to poor health outcomes, providing a clinical justification for a definition of missingness. After controlling for each patients’ total number of appointments, this project found that patients missing more than 2 general practice (GP) appointments per year on average over a 3-year period had multiple long-term physical and mental health conditions, experienced significant socioeconomic disadvantage, complex health needs, had poorer health outcomes and a significantly higher prevalence of premature mortality than patients who did not miss as many appointments [15–19]. This contributed to our current and less numerically stringent working definition of missingness as the repeated tendency not to take up offers of care, such that it has a negative impact on the person and their life chances [20]. We hypothesised that the causes of missingness are different in duration, complexity or intensity than those leading to single missed appointments. Interventions designed to tackle single missed appointments are likely be a poor fit for these patients and may *increase* access inequalities [21]. There is an urgent need to understand why missingness occurs, in order to intervene effectively and mitigate it and its negative impacts.

## Methods

We carried out a realist review of evidence to help us understand the causal dynamics underpinning missingness [22]. Realist approaches aim to develop a theory about the underlying dynamics causing or sustaining a problem [23]. They seek to explain “demi-regularities” or “semi-predictable patterns of behaviour” by exploring the key mechanisms underpinning an outcome pattern and the contexts in which they are activated ([24], p.2). The goal is to produce a realist programme theory that contains Context-Mechanism-Outcome configurations (CMOCs) outlining the mechanisms that cause an outcome and the contexts in which those mechanisms are activated [25].Theory is developed interpretatively by gathering and synthesising evidence from a range of documentary sources. This occurs in five stages, summarised in Table 1 [21, 22, 24]. Our review was registered with PROSPERO (ID CRD42022346006), and the methods are explained fully in the protocol [20]. The review follows the Realist And MEta-narrative Evidence Syntheses: Evolving Standards (RAMESES) project standards for realist synthesis [25, 26]. The views expressed are the authors and not necessarily those of the National Institute for Health and Care Research (NIHR) or the Department of Health and Social Care who funded the study via NIHR. The funders had no role in study design, data collection and analysis, decision to publish or preparation of the manuscript.

**Table 1:** Summary of realist review stages

Stage	Goal	Approach
1) Locating existing theories about causal dynamics;	Identify the existing understandings of what causes missingness or the theories underpinning why interventions work	- Team knowledge and expertise - Initial informal database searching - Consulting Stakeholder Advisory Group of 4 experts-by-experience and 4 professionals
2) Searching for a broad range of evidence from disparate sources	A cyclical, iterative approach to gathering evidence to advance programme theory development from disparate/varied sources	- Tracking backward/forward citation of 6 key reviews [27–32] - Searching bibliographic databases, ‘benchmarking’ against core papers [15–19, 29, 30, 32–38] - Identification of "grey literature”^a^ - Citation alerts for key terms and papers
3) Selecting sources of evidence which might contribute to theory development;	To select documents likely to provide contribute to development of our programme theory	- Deduplication of papers in Endnote X9 and export to DistillerSR - Screening of papers for relevance and rigour and labelling of excluded papers for iterative review - 10% cross-check of screening, discrepancies resolved through discussion
4) and 5) Extracting, organising and synthesising the evidence into an explanatory narrative	To describe and organise included documents. Coding text relevant to understanding causal dynamics, then using retroductive analysis to identify and articulate context-mechanism-outcome configurations that explain the key elements underpinning missingness	- Documentation of key characteristics in Excel - Extraction of data within NVIVO 12/14 and structuring into domains in which causal mechanisms might be found - 10% cross-check of coding - Analysis of data to identify key causal patterns - Summarising emerging findings for discussion and to guide further analyses, searching and interviews

^a^Via ETHOS (British Library Electronic Thesis Online); The King’s Fund Library Database; Revolving Doors Agency; Lankelly Chase; Doctors of the World; Faculty for Homelessness and Inclusion Health; FEANTSA; Health Foundation

### Overview of the evidence base

The findings below come from a total of 197 documents. The majority are from the UK (*n* = 110) or the USA (*n* = 37) and a significant proportion (*n* = 86) focus solely on primary care with others focused on other forms of healthcare provision. As such, while these findings are written with UK-based primary care in mind, they have a broader applicability across different service settings, geographical locations and health systems. A PRISMA diagram and description of included studies are included in Sects. 4 and 5 of Additional file 1. The most common study design explored statistical associations between patient/practice variables and missed appointments by exploring administrative data alone (*n* = 45), combined with questionnaires/surveys (*n* = 22) or with qualitative methods (*n* = 9). These studies vary substantially in their findings which may speak to unaddressed contextual heterogeneity or the use of different or contested variables—issue identified elsewhere in the literature [27, 29, 39–46]. They also provide limited explanatory insight or theoretical engagement [14, 39, 40, 47]. Survey and questionnaire studies (*n* = 27 alone, *n* = 29 mixed methods), designed to provide further causal insight, also contain issues of heterogeneity and definition, use pre-set, closed questions and present broad, prima facie reasons for non-attendance, and have been critiqued for obscuring complex causal realities [27, 48–51]. Studies report issues recruiting patients at risk of missing appointments, who may be less likely to participate in such research [42, 44, 52–59]. Some designs actively exclude relevant patient groups—for example, those with existing mental health issues, cognitive impairments, learning disabilities or who do not speak English [46, 48, 60–64]. Other methods are indirectly exclusionary, such as reliance on single, monolingual postal surveys, which may disadvantage those with no fixed address or who have literacy or language needs [45, 65, 66]. Research, like healthcare, has a “missingness” problem to address [45, 66].

In exploring missingness, there is a risk of perpetuating problematic narratives embedded within the existing literature. Language of non-attendance or non-engagement and related concepts like “non-compliance” [67], “failure to attend” [50], “chaotic” [32, 35, 68, 69] and “hard to reach” [70], p.14) all speak to patient-side problems and create a foundation for studies (and thus findings) that focus attention solely on patients. Often specific subgroups of patients, including those missing multiple appointments, are framed as particularly problematic. This was discussed by our Stakeholder Advisory Group, who felt that many patients *are not attended to* by services, and by literature suggesting that services, not patients, are “hard to reach” [41, 48, 71]. Other definitional challenges included the lack of shared definition of missed appointments—some studies include short-notice cancellations or lateness, while others do not—and of a shared definition of missingness, making comparability or generalisability harder [8–14, 46, 49, 72]. Others question whether missingness even exists as a meaningful category for study [40, 73]. Sections 7.2 of Additional file 1 reflects further on the process of synthesising a narrative from this challenging evidence base.

### Theoretical framework for the evidence synthesis

Despite these challenges, studies can still provide “evidential fragments” that can contribute to the programme theory through realist synthesis ([74], p135). The findings below are derived from this process, using fundamental causation theory and the candidacy framework to interpret the evidence [75–78]. Fundamental causation suggests that limited access to “flexible resources”—including knowledge, money, power, prestige and social connections—inhibits whether and how people pursue good health ([77], p.135). People’s “habitus”—unconscious or semi-conscious ways of knowing, being or acting, patterned by social position and which orient a person in the world—is also influential in health behaviours [75, 77]. A patient’s resources and habitus interact with the institutional processes of service-delivery to permit, enhance or block their health-promoting efforts [75, 77]. Candidacy similarly proposes that inequalities of service access/uptake occur in the interaction between the identities and resources of individuals seeking care (or not) and their (mis)alignment with structural and cultural qualities of services [78–80]. It outlines a series of domains: how patients come to identify themselves as candidates for a service; how they navigate to point-of-entry; how they align with service permeability/porosity—the cultures and structures governing access and use; how they present to services; how services respond with adjudications and offers which candidates, in turn, negotiate or resist [78]. This occurs within local operating conditions—the “localised cultural, organisational and political contexts” of services ([80], p.819). This approach supports a definition of non-attendance as the result of “a critical level of unsuitability in the agreed arrangements for an access episode” ([29], p.183)—an issue of the interaction between patients and healthcare providers. In missingness, this extends to an *enduring* unsuitability covering multiple access episodes. Our theoretical framework is explored further in Sect. 7.1 of Additional file 1, and the summary findings presented below are discussed in further detail in Sect. 7.3.

## Results

### Identification: is this “’for me?”

A patient’s decision of whether to attend is influenced by a range of beliefs and perceptions about themselves and their health and the perceived usefulness, importance or appropriateness of attending. Identification extends beyond the situational lens of the single appointment towards a wider identification around the *service,* unfolding over time. Ideas of ‘normal’ underpin a patient’s sense of acceptable or reasonable action (their habitus) and their sense of whether a service is ‘for me’ [40, 81]. Patients may not attend an appointment because they no longer see any need or purpose—symptoms may resolve or be mild, they may have few complications, minimal concern, feel their condition does not impact upon their everyday lives or is manageable without further input [14, 40, 42, 47, 56, 62, 64, 68, 82–86]. This may be particularly so in the context of long-term health conditions where many appointments are set by service providers, not requested by patients [13, 14, 27, 29, 37, 40, 46, 66, 82, 83, 86–95].

Conversely, some patients experience a high degree of worry or fear around their health or their appointments and may manage that through denial or avoidance [40, 46, 48, 62, 64, 96–99]. Many patients in adverse circumstances or with poor health have low expectations around what constitutes ‘normal’ health or may believe that healthcare can do nothing for them [8, 11, 48, 59, 62, 64, 66, 69, 96, 100, 101]. If past experiences of seeking care and support have not been beneficial, or provider and patient are “misaligned” ([11], p.4) in their understanding of the causes or solutions to health problems, or of the format or purpose of appointments, this might increase the sense that future attendance is of limited value [11, 50, 62, 64, 71, 83, 86, 93, 102, 103]. Identification may be influenced by the dynamics of specific conditions—mental health conditions, dementia, cognitive impairment—which influence how patients understand their health or need for healthcare [50, 87, 103]. Ideas of ‘normal’ exist within social relationships with peers, families or communities, moving the lens from ‘for me’ to for *us*’ [40, 46, 66, 81, 98, 103, 104]. Some candidacies may be suppressed by stigma, shame or embarrassment around health conditions; others by the belief that health services are unsuitable or inappropriate in cultural or community terms for the problems people experience [8, 48, 64, 99, 103, 105–107]. Crucially, these elements of identification can overlap or conflict or change over time—identities are neither static nor singular. The evidence synthesis underpinning this domain can be found in Sects. 7.3.1 and 7.3.2 of Additional file 1.

### Relational candidacies

Patients’ past experiences of services play a significant role in their willingness to attend in future and can impact on the sense that services are “for me” in relational terms*.* Difficult experiences in past appointments or appointment-making might involve patients feeling unheard or unseen, their perspectives or experiences discounted [11, 14, 44, 62, 66, 69, 93, 102–105, 108–111]. Unaddressed interpersonal communication needs may contribute to this—including lack of interpreting, language inaccessibility or power imbalances contributing to anxieties, mistrust or low confidence, all of which inhibit the giving or receiving of information [44, 45, 57, 64, 71, 94, 96, 111–113]. These may contribute to misalignment and the risk of mismatch between a patients’ needs and circumstances (their candidacies) and the adjudications and offers of services [101, 105, 108, 114]. It also creates a problematic power dynamic where patients feel disempowered, disrespected and excluded from their own care.

Relational dynamics are refracted through wider life experiences and circumstances, and some may be particularly at risk of problematic relational or power dynamics and their impacts. Some patients may have experienced judgemental, punitive or stigmatising interactions with services, whether because of missed appointments or other aspects of their lives or identities [11, 31, 40, 68, 69, 96, 101, 103, 105, 111, 113–115]. Stigma is part of fundamental causation because it increases exposure to identity-threatening encounters while reducing interpersonal power, making people less likely to be acknowledged or heard within them [116]. The internalisation and anticipation of stigma or hostility is thus a central part of missingness, resulting in reactive avoidance to prevent relational threat or the threat to identity, even in circumstances of urgent need [46, 96, 104, 105, 111, 117]. Interpersonal encounters with unequal power dynamics may be particularly challenging for patients with difficult experiences of caring relationships and histories of psychological trauma, abuse, attachment issues, neglect or violence, contributing to approach-avoidance—where appointments have the potential to resolve health issues but are also the source of exposure to unmanageable anxiety and stress [8, 11, 33, 40, 45, 50, 71, 93, 108, 112]. The risk of mistrust is greater where systems do not support continuity of clinician, sufficient time within appointments or adequate communication support [11, 13, 14, 29, 32, 40, 42, 44, 46, 47, 51, 69, 82, 86, 90, 91, 93, 103, 105, 108, 118–120]. The evidence synthesis underpinning this domain can be found in Sects. 7.3.2 and 7.3.3 of Additional file 1.

### Competing candidacies, multiple demands and limited resources

Patients experiencing missingness may be positioned in precarious socioeconomic circumstances that expose them to disruptive forces that threaten health, material and social wellbeing [9, 11, 17–19, 43, 49, 59, 62, 65, 71, 96, 103]. Ideas of competing demands or “conflicting candidacies”([79], p.56) shows how multiple urgent and competing priorities might result in reduced prioritisation of health or appointment attendance relative to other needs [30, 32, 36, 40, 50, 55, 57, 59, 62, 82, 83, 99, 102, 103, 107, 121–128]. By virtue of socioeconomic position, patients may have reduced access to the flexible resources to protect them against these forces, while experiencing multiple pressures on the personal, material and social resources they do have access to [77]. These demands include treatment burden and other appointments [4, 10, 11, 15, 17, 19, 28, 41, 43, 44, 48, 59, 61, 62, 64, 73, 83, 84, 86, 93, 100, 128–135]; employment responsibilities, employer inflexibility and the financial costs of missing work [11, 40, 44, 46–49, 59, 64, 81, 106, 107, 109, 115, 123, 135–137]; and caring responsibilities taking precedence [14, 40, 41, 44, 48, 59, 64, 99, 131, 138]. Patients experiencing severe and multiple disadvantages may have to prioritise accommodation, physical safety and survival demands over healthcare [43, 48, 53, 64, 65, 69, 71, 81, 96, 103, 114, 139]. Travelling to appointments requires resources that may be limited or needed to meet other demands [27, 28, 30–32, 36, 39, 40, 47, 55, 56, 83, 90, 125, 126, 140]. Disabilities or severe physical or mental health symptoms can make travelling to appointments unsafe or unmanageable [8, 9, 13, 15, 30–32, 36, 40, 41, 44–50, 54, 55, 58, 60–62, 64, 65, 68, 82, 84, 86, 89, 90, 96, 99, 100, 103, 105–110, 122, 125, 126, 128, 129, 133, 141–150]. Social connections and their resources may be beneficial but are not always available to help [11, 44, 45, 48, 49, 62, 63, 65, 101, 103, 112, 122, 126, 130, 134]. Evidence synthesis Sects. 7.3.4, 7.3.6, and 7.3.7 contribute to the findings in this domain.

### Permeability and appointment-making

Primary care in the UK is a gatekeeper-led system, with a specific structure and certain cultural and behavioural expectations that must be met to navigate it successfully. Patients experiencing missingness may struggle with these aspects of permeability [78]. Registration and appointment systems in general have been described as a “threat” ([78], p.6) to some patients and a contributor to health inequalities [71, 101, 117]. By not attending in the past, patients are positioned as deviant or troublesome in the eyes of staff, creating “friction” ([115], p.642) and many of the relational challenges above [19, 35, 36, 40, 68, 69, 96, 115, 118, 137, 151]. Appointment-making requirements might be narrow and hard for patients to fulfil—calling at certain times, with telephone-only systems, to monolingual staff—and the personal resources (knowledge, skills, confidence, language) needed to fulfil these expectations or to negotiate and persist in pursuit of the right appointment are unequally distributed or depleted by negative experiences with services [18, 32, 47, 58, 69, 96, 105, 117, 137]. If services do not offer a timely appointment, patient circumstances change—they may forget, symptoms resolve, motivations change or other demands emerge, accounting for the almost universal finding that delays lead to missed appointments [18, 27–32, 39, 40, 51, 69, 90, 91, 96, 119, 152] [9–11, 18, 19, 27, 35, 37, 40, 46, 50, 51, 69, 82, 100, 101, 103, 109, 118, 122, 137, 138, 153, 154]. If services do not offer a convenient time, patients may not be able to reconcile attendance with competing demands or with the rhythms and patterns of their lives [9, 11, 29, 32, 37, 40–42, 46, 47, 49–51, 61, 66, 83, 87, 96, 100, 101, 106, 109, 111, 113–115, 123, 128, 131, 135, 155]. If the system does not permit or support patient choice in whom they see, in where or how they attend (in-person/virtually/at home/in practice), in communication support or in how much time is afforded to them, candidacies might be disrupted [18, 40, 44, 57, 59, 66, 101, 137, 148, 156, 157]. Errors, miscommunications or misunderstandings may be caused by service factors: failures to notify patients of appointments; providing incorrect details; booking appointments or notifying patients at short notice; using inaccessible or unsuitable forms of communication; and not providing ways for patients to easily amend or cancel their appointments [11, 29, 30, 36, 40, 41, 44–47, 54, 55, 57, 59, 61, 82, 86, 88–90, 115, 117, 122, 127, 128, 132–135, 142, 143, 145, 147, 158]. Sections 7.3.5, 7.3.7 and 7.3.3 of Additional file 1 provide the evidence synthesis for these findings.

### The importance of local operating conditions

McLean et al. [40] suggest that non-attendance comes from service inaccessibility, inflexibility or unsuitability and recommend “diagnosing which of these pathologies is predominant in a particular setting” (p.101). Yet, research is often limited in reporting on the contextual conditions of service settings or the influence of specific local circumstances on findings. When reported, this information is often limited to patient demographics and numerical staffing data or brief description of some elements of service administration such as hours of operation, booking systems or any existing measures to reduce non-attendance. These are rarely then integrated into findings. Studies exploring administrative data across multiple settings can often make it appear as though missingness occurs in an institutional, political and economic vacuum.

The influence of local conditions can be inferred from the findings above—for example, in whether service staff hold stigmatising attitudes; whether staffing and appointment systems support timely or convenient access or relational continuity; in what additional services are offered within a practice, health centre or wider community. Less well-articulated are the wider structural influences on service delivery—staffing, resourcing and the wider political economy of healthcare. In response to recent UK government statements around non-attendance, the Royal College of General Practitioners points towards an existential crisis in primary care around workload, workforce and staff turnover impacting upon appointment availability and service quality [159–161]. Included studies suggest that these issues are likely to limit appointment availability (and thus convenience and flexibility), time spent with patients or the emotional resources of practitioners to participate in person-centred, trauma-informed care [40, 66, 101, 103, 162]. Models of funding that are based on numerical targets may actively discourage in-depth engagement with patients with more complex needs [66, 69, 71, 117]. Several papers discuss the benefits of specialist primary care provision for groups experiencing access issues, but these services often face insecure and limited funding and local variability and may risk ‘ghettoising’ primary care rather than seeking to improve it [69, 71, 101, 105, 111]. Candidacy issues in other areas of the health and social care system are also relevant. Issues of treatment burden connect to whether care is integrated or co-located or whether collaboration exists between services. If not, patients may fall into gaps, and many experience difficulties navigating referral thresholds and eligibility criteria, leaving them without adequate support and with reduced trust in the system to meet their needs [46, 62, 69, 71, 101, 103, 108, 111, 112, 163]. In the UK, these issues are exacerbated by austerity, with impacts on the resources available to local authorities, third sector organisations, and to many patients themselves. More recently, the cost of living crisis has impacted on the resources available for people to travel to appointments, and to access medications, and other essentials required to maintain or manage their health [140]. The absence of many of wider contextual issues from the existing research means that many causal influences remain unseen.

## Discussion

These findings have several implications. There is a pressing need to move from framing missed appointments as an issue *for* services created *by* patients, towards perspectives exploring the interaction between patients’ circumstances and service dynamics [29, 40, 78]. Studies into non-attendance would benefit from including a ‘missingness’ perspective in their design—identifying missingness in administrative data, actively seeking the perspectives of those experiencing missingness using inclusive methods of recruitment and data collection and stratifying findings to report on missingness rather than just missed appointments. Positive examples in this review include those studies comparing findings around non-attendance, health outcomes and circumstances for those experiencing missingness with those who miss fewer or no appointments [15–19, 56, 58, 64, 93]. Reflections on the representativeness of samples (including non-respondents) would be beneficial for contextualising findings [55, 59, 95]. Further explication of local operating conditions would also support contextualisation of heterogeneous research findings [27, 46, 103]. Finally, we suggest that non-attendance research rebalance the disproportionate focus on statistical associations and surveys with in-depth research (e.g. qualitative or mixed-methods) and work underpinned by substantive theoretical engagement [14, 39, 40, 47]. The emergence of literature focused exclusively on machine-learning and predictive algorithms is not encouraging in this respect, not least because those models are built upon a flawed evidence base.

### Emerging principles for policy and practice

Our interpretation of the evidence suggests that to design services that are more equitable and account for the complex reasons underlining missingness, systems should be designed to identify ‘missing’ patients and interventions tailored to the needs of these patients. Papers included in this review suggest a need to identify and actively reach out to patients experiencing missingness to discuss their candidacy experiences, both to allow tailoring of interventional approaches and to support service design based on patient experience. Dumontier et al. [9] and others suggest that these conversations help build a local evidence base and act as a first line of intervention:

“Personal, respectful, and supportive contact both improves health care providers’ understanding of their patients’ individual and collective issues and may decrease anxiety that those patients may have about seeking care.” (p.640).

Others suggest proactive, structured assessments of domains related to non-attendance (e.g. mental health, particularly depression and anxiety [33, 106, 110, 129]; post-traumatic stress disorder, attachment and Adverse Childhood Experiences [8, 11, 33]; or ‘patient activation’ [58, 164]) or more holistic assessments of patients’ needs and circumstances [41, 105, 163]. Interventions can then be designed with these patients in mind, tailored to them, or evaluated according to effectiveness or appropriateness for ‘missing’ patients [8, 9, 31, 33, 39, 41, 42, 58, 71, 72, 105, 110, 129, 130]. Without tailoring, interventions designed to address missed appointments risk worsening inequalities of access [13, 18, 27, 59, 63, 69, 81, 95, 98, 117, 148, 155, 165–172].

Tailoring and targeting of interventions will be more beneficial if they target multiple causal domains and the interaction between patients and services. Often, policy and practice are designed to tackle a single domain identified in the literature and often focus solely on patient cognition or behaviour—for example, using reminders for forgetfulness or behavioural “nudges” to encourage the ‘correct’ patient behaviour [3, 119, 162, 173–176]. These perpetuate the inaccurate and stigmatising notion that patients are the primary cause of non-attendance, precluding action on other influences. Targeting one domain in isolation is unlikely to resolve the multiple, overlapping causes of missingness outlined here, and any complex intervention must go beyond perspectives on patients’ behaviours to address structural forces, service design and organisational cultures [27, 39, 40, 98, 104, 122, 174].

The evidence above shows that missingness is driven by dynamics where patients feel unheard, disempowered, unsafe or threatened. Promoting relational, cultural and structural safety involves identifying and addressing problematic relational dynamics and power differentials within services, while acknowledging how patients’ candidacies are impacted by the intersections of their broader relational, cultural and structural circumstances [10, 11, 40, 42, 44, 45, 59, 71, 87, 103, 104, 120, 177, 178]. Work is required to address how stigma, judgement and dehumanisation are communicated or to address the mistrust caused by relational difficulties or wider social exclusion [41, 42, 44, 46, 66, 69, 71, 93, 96, 101, 105, 108, 111, 117, 163]. Building on trauma-informed, patient-centred and culturally safe principles, healthy relationships have benefits in themselves and act as the scaffolding upon which other interventions can be built [11, 32, 40, 42, 44, 45, 57, 59, 66, 71, 87, 90, 94, 101, 120, 122, 163, 179].

## Conclusions

This realist review has sought to identify and understand the factors which lead to multiple missed appointments, differentiating the situational causal dynamics of single missed appointments from the enduring dynamics that underpin ‘missingness.’ Patients may not attend because of a belief that services and their offerings are not “for them”—not suitable, beneficial or appropriate for their needs or their identities. This can reflect a lifetime of experiences with services as well as other meaningful relationships that shape patients’ sense of self in relation to health services and to caring relationships. Attendance may not feel beneficial or worthwhile, or it may be a source of unmanageable anxiety or threat. Negative past relational experiences and anticipated future difficulties, including disempowerment, hostility and stigma, make patients feel unsafe and unwelcome. Processes of prioritisation for those exposed to multiple, competing demands and urgencies can interfere with healthcare, and patients’ resources may be insufficient to support the management of competing candidacies or the logistics of attendance. Appointment systems may prevent access to timely, convenient, relationally accessible care. These domains overlap and mutually reinforce, and the content, configuration and relative influence of each vary between patients and settings.

The strength of realist review is in its theory-driven synthesis of a diverse evidence base, creating a coherent, evidence-informed narrative to inform future action. The use of candidacy and fundamental causation has led to a rich and nuanced understanding of the multiple, overlapping influences in missingness, which operate at the micro-, meso- and macro-level. The main limitations of this review relate to the evidence base. The reliance on administrative data and surveys/questionnaires, and the lack of a missingness perspective in research design and reporting, means that parents experiencing missingness are also ‘missing’ from research and public policy conversations around healthcare provision. Realist review allows for some of these gaps to be addressed and enables us to draw inferences from a wide evidence base. There remains a need for new research to broaden and deepen this evidence base so that those seeking to address missingness can act from firm foundations.

There is a need to reframe the problem from one of patient behaviour causing challenges for services and towards an understanding of issues in access/quality in the interaction between patients and services. Moreover, if missingness is an issue of access to and quality of care, it is only one part of the mutually reinforcing relationship between socio-economic status and health. We have to stay open to the important challenge of what contribution this emphasise on service provision makes when wider structural factors are so pervasive [104]. Health services need to be at their best where they are needed most to have a chance of doing this and reversing the inverse care law [180].

### Supplementary Information

Additional file 1: Supplementary files.

## Data Availability

The papers reported in the realist evidence synthesis are listed  in the supplementary file along with the CMOCs which underpin the results. When the paper is published these will be available on the overall study Openscience Framework webpage.

## Abbreviations

CMO or CMOC: Context-Mechanism-Outcome or Context-Mechanism-Outcome Configuration
GP: General practice/general practitioner
NHS: National Health Service
NIHR: National Institute for Health and Care Research
PTSD: Post-traumatic stress disorder
RAMESES: Realist And MEta-narrative Evidence Syntheses: Evolving Standards
