# Supplementary material for: Understanding the causes of missingness in primary care: a realist review
Source: BMC Med. 2024 Jun 10;22:235. doi: 10.1186/s12916-024-03456-2 (PMC11165900; doi:10.1186/s12916-024-03456-2)
Supplement: Supplementary file 1 — Additional file 1: Supplementary files. [file 12916_2024_3456_MOESM1_ESM.docx]

Contents

1. Initial Programme Theory............................................................................................ 2

2. Study flow diagram............................................................................................................ 3

3. Example search.................................................................................................................... 4

4. PRISMA diagram.............................................................................................................. 5

5. Study characteristics.......................................................................................................... 7

6. List of included documents........................................................................................... 8

7. Evidence Synthesis and CMO Configurations............................................................ 32

7.1 Synthesising framework..................................................................................................... 32

7.2 Navigating and synthesising the evidence............................................................................... 33

7.3 Synthesised Findings.............................................................................................................. 34

7.3.1 Identification – is healthcare ‘for me’? ................................................................................ 34

7.3.2 Past presentation, adjudication and relational dynamics.................................................... 36

7.3.3 Presentation, adjudication and stigma.............................................................................. 38

7.3.4 Competing demands, competing candidacies and limited resources.............................. 40

7.3.5 Permeability and ‘ease of access’........................................................................................... 43

7.3.6 Navigation and Presentation - Forgetting............................................................................ 46

7.3.7 Navigation and Presentation: Getting There.................................................................. 47

8. CMOCs and illustrative data............................................................................................. 49

9. Evidence synthesis bibliography.................................................................................. 57

# Initial Programme Theory

This information can also be found in our published research protocol (1).

We carried out an initial scoping search of MEDLINE and Web of Science (Science and Social Science Citation indexes) in January 2022 to inform the conceptualisation phase of the project using keywords describing repeated missed appointments, low engagement/uptake and attendance in healthcare to explore the available existing literature. Through further exploratory and informal searching an initial programme theory was created, shaped around the candidacy framework as an initial theoretical/conceptual frame (2, 3). This framework suggests that “missingness” is an expression of inequalities in access to, and utilisation of, health services. It provides a heuristic framework for moving beyond simplistic ideas of access to understand service engagement as a journey, one that proceeds in several connected and non-linear stages. Our initial programme theory suggests that dynamics within and between stages of this process influence whether a person becomes “missing”:

- Identification: some patients may not view themselves as legitimate and worthy candidates for primary care, or may feel the support provided is not for them or will not benefit them.
- Navigation: patients may have limited resources to overcome practical barriers including travel costs, phone credit, limited knowledge of how to navigate systems, or multiple and competing life priorities that are hard to prioritise.
- Permeability and porosity: GP services may be difficult to access – inflexible appointment times or systems, long waits for appointments, gatekeeping practices, no choice of clinicians or no access to additional support – that are poorly aligned with the resources or preferences of patients.
- Presentation: The act of presenting to general practice may be less of a priority in complex life circumstances. Poor physical or mental health may inhibit to ability to attend, while patients may experience difficulties communicating or being heard in appointment spaces. Prior experiences of stigma or exclusionary practice may inhibit or constrain people’s willingness or ability to present.
- Adjudication: where practitioners make judgements about patients’ health, often these can stigmatise or feel exclusionary and limit future engagements. GP service dynamics may mean adjudications are made on heuristics, categorisations and moral schemas of staff, and there is limited space for trusted relationships to be formed or maintained.
- Offers and resistance: Problems in adjudication can mean the offers made to patients do not meet their hopes or expectations and can inhibit future engagement with services. Often, services view such non-engagement as patient negligence or choice rather than a form of resistance or negotiation that could be addressed by providing care through a “missingness” lens.
- Local operating conditions: the resourcing and context of service provision may prevent identification of patients at risk of “missingness”, may create narrow routes for access and inhibit presentation and adjudication. Professional practice may not meaningfully account for those who are “missing” and respond with support or improved practice (2).

The framework will likely be complemented by other substantive theories in health inequalities, including structural vulnerability (4), fundamental cause theory (5), and other theories that speak to the causal dynamics underpinning the candidacy process.

# Study flow diagram

Adapted from Wong et al 2015 (6).

Step 1: Locate existing theories

- Informal searching
- Team knowledge and expertise
- Input from stakeholder advisory group.
- Develop initial programme theory

Step 2: Search for evidence

(with Information Specialist input)

- Develop, pilot and refine search, benchmarked against core papers
- Identification of grey literature
- Citation tracking from key papers.
- Citation alerts for key terms and papers.

Step 3: Article selection

- Relevance
- Rigour
- Retaining some excluded papers for future review.

Step 4: Extracting and data organising:

- Spreadsheet
- NVivo

Step 5: Synthesising the evidence (and drawing conclusions)

Refined programme theory

- Additional searching as needed.
- Iterative review of previously excluded studies.

Present refined theory to stakeholder advisory group for final input.

Design of a complex intervention based on refined programme theory. (forthcoming)

# Example search

| **Medline (Ovid MEDLINE® Epub Ahead of Print, In-Process & Other Non-Indexed Citations, Ovid MEDLINE® Daily and Ovid MEDLINE®) 1946 to present** | |  |
| --- | --- | --- |
| 1 | (non-attend* or "failure to attend" or no-show* or "no show*").ti. | 734 |
| 2 | ((sporadic or non-regular or irregular or low or pattern*) and (attend* or engag*)).ti. | 1249 |
| 3 | ((miss* or broken or break* or non-utili?ed or fail* or keep* or unkept* or cancel* or adher* or default*) and appointment*).ti. | 620 |
| 4 | (miss* and (care encounter* or care opportunit*)).ti. | 8 |
| 5 | ((low or pattern or under or poor) and attend*).ti. | 692 |
| 6 | (care and avoid*).ti. | 800 |
| 7 | *No-Show Patients/ | 187 |
| 8 | *"Appointments and Schedules"/ and *Patient Compliance/ | 469 |
| 9 | or/1-8 | 3907 |
| 10 | exp United Kingdom/ | 388051 |
| 11 | (national health service* or nhs*).ti,ab,in. | 261866 |
| 12 | (english not ((published or publication* or translat* or written or language* or speak* or literature or citation*) adj5 english)).ti,ab. | 47986 |
| 13 | (gb or "g.b." or britain* or (british* not "british columbia") or uk or "u.k." or united kingdom* or (england* not "new england") or northern ireland* or northern irish* or scotland* or scottish* or ((wales or "south wales") not "new south wales") or welsh*).ti,ab,jw,in. | 2400533 |
| 14 | (bath or "bath's" or ((birmingham not alabama*) or ("birmingham's" not alabama*) or bradford or "bradford's" or brighton or "brighton's" or bristol or "bristol's" or carlisle* or "carlisle's" or (cambridge not (massachusetts* or boston* or harvard*)) or ("cambridge's" not (massachusetts* or boston* or harvard*)) or (canterbury not zealand*) or ("canterbury's" not zealand*) or chelmsford or "chelmsford's" or chester or "chester's" or chichester or "chichester's" or coventry or "coventry's" or derby or "derby's" or (durham not (carolina* or nc)) or ("durham's" not (carolina* or nc)) or ely or "ely's" or exeter or "exeter's" or gloucester or "gloucester's" or hereford or "hereford's" or hull or "hull's" or lancaster or "lancaster's" or leeds* or leicester or "leicester's" or (lincoln not nebraska*) or ("lincoln's" not nebraska*) or (liverpool not (new south wales* or nsw)) or ("liverpool's" not (new south wales* or nsw)) or ((london not (ontario* or ont or toronto*)) or ("london's" not (ontario* or ont or toronto*)) or manchester or "manchester's" or (newcastle not (new south wales* or nsw)) or ("newcastle's" not (new south wales* or nsw)) or norwich or "norwich's" or nottingham or "nottingham's" or oxford or "oxford's" or peterborough or "peterborough's" or plymouth or "plymouth's" or portsmouth or "portsmouth's" or preston or "preston's" or ripon or "ripon's" or salford or "salford's" or salisbury or "salisbury's" or sheffield or "sheffield's" or southampton or "southampton's" or st albans or stoke or "stoke's" or sunderland or "sunderland's" or truro or "truro's" or wakefield or "wakefield's" or wells or westminster or "westminster's" or winchester or "winchester's" or wolverhampton or "wolverhampton's" or (worcester not (massachusetts* or boston* or harvard*)) or ("worcester's" not (massachusetts* or boston* or harvard*)) or (york not ("new york*" or ny or ontario* or ont or toronto*)) or ("york's" not ("new york*" or ny or ontario* or ont or toronto*))))).ti,ab,in. | 1703187 |
| 15 | (bangor or "bangor's" or cardiff or "cardiff's" or newport or "newport's" or st asaph or "st asaph's" or st davids or swansea or "swansea's").ti,ab,in. | 68381 |
| 16 | (aberdeen or "aberdeen's" or dundee or "dundee's" or edinburgh or "edinburgh's" or glasgow or "glasgow's" or inverness or (perth not australia*) or ("perth's" not australia*) or stirling or "stirling's").ti,ab,in. | 250905 |
| 17 | (armagh or "armagh's" or belfast or "belfast's" or lisburn or "lisburn's" or londonderry or "londonderry's" or derry or "derry's" or newry or "newry's").ti,ab,in. | 32815 |
| 18 | or/10-17 | 3013111 |
| 19 | (exp africa/ or exp americas/ or exp antarctic regions/ or exp arctic regions/ or exp asia/ or exp australia/ or exp oceania/) not (exp United Kingdom/ or europe/) | 3280931 |
| 20 | 18 not 19 | 2853824 |
| 21 | (primary care or primary health* or family physician* or general practi* or family practi* or outpatient? or clinic? or ambulatory or health centre? or health center? or office).ti,ab. | 925862 |
| 22 | 9 and 20 and 21 | **290** |

# PRISMA diagram

**Identification of studies: Initial Search**

Duplicate records removed from databases (n=692)

Records identified from Databases (n=1462)

**Remaining papers:1,372**

Duplicate records removed from combining database and citation records (n=84)

Duplicate records removed in Distiller (n=4)

Duplicate records removed from citations (n=189)

Records identified from citations from/of existing reviews (n=879)

**Identification**

Records identified from other sources:

colleagues, grey literature, NHS documents, (n=101)

citation alerts for core papers (n = 14)

**Documents remaining for title and abstract screening – 1487**

Records excluded with reasons: (n=1210)

NHS but not primary care (inc. secondary, outpatient, screening etc) (n = 193)

Missed appointments but non-NHS primary care (other countries) (n = 108)

Missed appointments in non-health services, UK (n = 1)

Missed appointments, other health services, non-UK/location unclear (n = 465)

None of the above/not relevant (n = 443)

Records included: 277

Meets all criteria: (n = 127)

Unclear/other reasons for full-text inclusion (n = 150)

**Title and abstract screening**

**Documents included (n=85)**

Excluded, with labels:

No – moved to background (n = 82)

No – relabelled, excluded (n = 104)

Duplicates removed: (n = 4)

**Full-text screening**

Records included (n = 277)

Texts not retrieved: (n = 2)

**Full texts reviewed (n = 275)**

**Identification of studies: revisiting excluded papers**

**UK, non-primary care:**

- papers excluded (n=208)
- protocols/posters removed (n=2) and replaced by related peer-reviewed studies (n=4 added).

**UK, non-primary care papers included: (n=42)**

Non-UK, primary care papers excluded (n=99)

**Non-UK, primary care papers included (n=37)**

**Documents included: 79**

Papers about non-attendance (UK, non-primary care) (n=248)

Papers about non-attendance (primary care, non-UK): (n=136)

**Identification of studies: Other sources**

**Total number of studies: 197**

Additional grey literature: **5**

Additional papers from ‘pearling’/found opportunistically: **15**

Citation alerts: **13**

**Documents included: 33**

# Study characteristics

| **Location** | **No. of studies** | **Health setting** | **No. of studies** |
| --- | --- | --- | --- |
|  |  |  |  |
| Australia | 3 | Primary care | 87 |
| Canada | 6 | Outpatient/specialist – physical health | 38 |
| Denmark | 3 | Mental health/substance use | 13 |
| Germany | 2 | Non-health settings | 3 |
| Israel | 1 | Healthcare general | 10 |
| Malaysia | 1 | Multiple settings | 15 |
| n.a/other | 31 | Other/n.a/not stated | 31 |
| New Zealand | 1 |  |  |
| Switzerland | 1 |  |  |
| UK | 110 |  |  |
| USA | 38 |  |  |
|  |  |  |  |
| **Study design/methods** |  |  |  |
|  |  |  |  |
| Qualitative (interview, focus group, observation, or combination of these) | 30 |  |  |
| Systematic review, realist synthesis or meta-analysis | 18 |  |  |
| Literature review, non-systematic | 10 |  |  |
| Quantitative (administrative data inc. retrospective, observational, linkage studies) | 45 |  |  |
| Questionnaire/survey | 27 |  |  |
| Mixed (administrative data + questionnaire/survey) | 22 |  |  |
| Mixed (administrative + qualitative data) | 9 |  |  |
| Mixed (other) | 8 |  |  |
| Randomized trials | 6 |  |  |
| Theoretical/conceptual papers | 9 |  |  |
| Policy/guidance documents | 3 |  |  |
| Opinion piece/news article/press releases/letters | 10 |  |  |

# List of included documents

| **Author** | **Title** | **year** | **Source** | **Setting + Geographical area** |
| --- | --- | --- | --- | --- |
| Abdulkadir, L.S.; Mottelson, I.N.; Nielsen, Dorthe, S. | Why does the patient not show up? Clinical case studies in a Danish migrant health clinic | 2019 | Eur J Pers Cent Healthc. 2019;7(2):316-24. | Denmark (migrant health clinic) |
| Aggarwal, A., Davies, J., Sullivan, R. | "Nudge" and the epidemic of missed appointments Can behavioural policies provide a solution for missed appointments in the health service? | 2016 | Journal of Health Organization and Management, 30(4) | n/a (review paper) |
| Akter, S., Doran, F., Avila, C., & Nancarrow, S. | A qualitative study of staff perspectives of patient non-attendance in a regional primary healthcare setting | 2014 | Australas Med J. 2014;7(5):218-26. | Australia (primary care) |
| Amberger, C & Schreyer, D. | What do we know about no-show behavior? A systematic, interdisciplinary literature review | 2022 | J Econ Surv. 2022;38:57–96 | n/a (review paper) |
| Anyaegbu, C.T. | SMS reminders: reducing DNA at a community mental health depot clinic. | 2021 | Journal of Community Nursing, 35(1). | UK (outpatient - community mental health) |
| Arber, S. & Sawyer, L. | Do appointment systems work | 1982 | BMJ. 1982;284(6314):478-80. | UK (primary care) |
| Arnold, O.F. | Reconsidering the “NO SHOW” Stamp: Increasing Cultural Safety by Making Peace with a Colonial Legacy | 2012 | Northern Review (26) | Canada (primary care) |
| Aspinall, P.J. | Inclusive practice: vulnerable migrants, gypsies and travellers, people who are homeless, and sex workers: a review and synthesis of interventions/service models that improve access to primary care & reduce risk of avoidable admission to hospital | 2014 | https://assets.publishing.service.gov.uk/media/5a7db8b2e5274a5eaea65ee4/Inclusive_Practice.pdf | UK (primary care) |
| Ayalde, J., Soong, W., Thomas, S., McCann, P., Griffiths, J., Nicholls, C., Heble, S., Dragovic, M. & Waters, F. | Reasons for non-attendance in youth mental health clinics: Insights from mobile messaging communications | 2022 | Early Interv Psychiatry, 2023;17(9):877-83. | Australia (outpatient – mental health) |
| Ballantyne, M. Liscumb, L., Brandon, E., Jaffar, A., Macdonald, A., Beaune, L. | Mothers’ Perceived Barriers to and Recommendations for Health Care Appointment Keeping for Children Who Have Cerebral Palsy | 2019 | Glob Qual Nurs Res. 2019;6. | Canada (primary care) |
| Bansal, I., Soni, R., Eisen, S., Ward, A., Longley, N., & Sen, C. | Breaking the Barriers to accessing care: co-creating solutions with refugee service users | 2023 | Arch Dis Child. 2023;108. | UK (primary care) |
| Barker, I., Steventon, A., Williamson, R. & Deeny, S.R. | Self-management capability in patients with long-term conditions is associated with reduced healthcare utilisation across a whole health economy: cross-sectional analysis of electronic health records | 2018 | BMJ Qual Saf. 2018;27(12):989-99. | UK (primary care) |
| Barron, W.M | Failed appointments - who misses them, why they are missed, and what can be done | 1980 | Prim Care. 1980;7(4):563-74. | n/a (review paper) |
| Bean, A.G. & Talaga, J. | Appointment breaking: causes and solutions | 1992 | J Health Care Mark. 1992;12(4):14-25. | n/a (review paper) |
| Bech, M. | The economics of non-attendance and the expected effect of charging a fine on non-attendees | 2005 | Health Policy, 74(2) | Denmark (health general) |
| Bickler, C. B. | Defaulted appointments in general practice | 1985 | J R Coll Gen Pract. 985;35(270):19-22. | UK (primary care) |
| Biggs, J., Njoku, N., Kurtz, K., Omar, A. | Decreasing Missed Appointments at a Community Health Center: A Community Collaborative Project | 2022 | J Prim Care Community Health. 2022;13:3. | USA (primary care) |
| Blankenstein, R. | Failed appointments - Do telephone reminders always work? | 2003 | Clin Gov. 2003;8(3):208-12. | UK (primary care - dental) |
| Boksmati, N., Butler-Henderson, K., Anderson, K., & Sahama, T. | The Effectiveness of SMS Reminders on Appointment Attendance: a Meta-Analysis | 2016 | J Med Syst. 2016;40(4):10. | n/a (review paper) |
| Boos, E.M., Bittner, M.J. & Kramer, M.R. | A Profile of Patients Who Fail to Keep Appointments in a Veterans Affairs Primary Care Clinic | 2016 | WMJ. 2016;115(4):185-90. | USA (primary care - veterans affairs) |
| Boshers, E. B., Cooley, M. E. & Stahnke, B. | Examining no-show rates in a community health centre in the United States | 2021 | Health Soc Care Community. 2022;30(5):e2041-e9. | USA (primary care) |
| Bowser, D.M., Utz, S.G, Doris,Harmon, Rebecca | A Systematic Review of the Relationship of Diabetes Mellitus, Depression, and Missed Appointments in a Low-Income Uninsured Population | 2010 | Arch Psychiatr Nurs. 2010;24(5):317-29. | n/a |
| Brewster, S. | The role of community pharmacy in supporting people with diabetes who have a history of repeated non-attendance at healthcare appointments | 2023 | Doctoral dissertation, University of Southampton | UK (primary care - pharmacy - diabetes) |
| Brown, Sarah | Qualitative evaluation of Focused Care | 2019 | https://focusedcare.org.uk/wp-content/uploads/2020/11/FC-Qualitative-Eval.pdf | UK (primary care) |
| Buetow, S. | Non-attendance for health care: When rational beliefs collide | 2007 | Sociol Rev. 2007;55(3):592-610. | n/a |
| Bull, S.L., Frost, Nicki,Bull, Eleanor R. | Behaviourally informed, patient-led interventions to reduce missed appointments in general practice: a 12-month implementation study | 2022 | Fam Pract. 40(1) | UK (primary care) |
| Cameron, E. | A mixed methods investigation of parental factors in non-attendance at general paediatric hospital outpatient appointments | 2015 | Doctoral dissertation, Aston University | UK (primary care + outpatient - paediatrics) |
| Cameron, E., Heath, G., Redwood, S., Greenfield, S., Cummins, C., Kelly, D., Pattison, H. | Health care professionals' views of paediatric outpatient non-attendance: implications for general practice | 2014 | Fam Pract. 2014;31(1):111-7. | UK (primary care + outpatient) |
| Campbell, K., A. Millard, G. McCartney, and S. McCullough. | Who is least likely to attend? An analysis of outpatient appointment ‘Did Not Attend’ (DNA) data in Scotland | 2015 | https://www.healthscotland.scot/media/1129/5348_dna-analysis_nhs-ggc.pdf | UK (outpatient - various) |
| Campbell-Richards, D. | Exploring diabetes non-attendance: An Inner London perspective | 2016 | J Diabetes Nurs. 2016;20(2):73-8. | UK (outpatient - diabetes) |
| Car, J., Sheikh, A. | Telephone consultations | 2003 | BMJ. 2003;326(7396):966-9. | n/a (review paper) |
| Car, J., Ng, C., Atun, R., Card, A. | SMS Text Message Healthcare Appointment Reminders in England | 2008 | J Ambul Care Manage. 2008;31(3):216-9. | UK (health general) |
| Cashman, S. B., Savageau, J. A., Lemay, C. A., Ferguson, W. | Patient Health Status and Appointment Keeping in an Urban Community Health Center | 2004 | J Health Care Poor Underserved. 2004;15(3):474-88. | USA (primary care) |
| Chapman, K.A., Machado, S.S., van der Merwe, K., Bryson, A., Smith, D. | Exploring Primary Care Non-Attendance: A Study of Low-Income Patients | 2022 | J Prim Care Community Health. 2022;13. | USA (primary care) |
| Chen, K., Zhang, C., Gurley, A., Jackson, H., Akkem, S. | Appointment Non-attendance for Telehealth Versus In-Person Primary Care Visits at a Large Public Healthcare System | 2023 | J Gen Intern Med. 2023 Mar;38(4):922-8. | USA (primary care) |
| Ciechanowski, P., Russo, J., Katon, W., Simon, G., Ludman, E., Von Korff, M., Young, B., Lin, E. | Where is the patient? The association of psychosocial factors and missed primary care appointments in patients with diabetes | 2006 | Gen Hosp Psychiatry. 2006;28(1):9-17 | USA (primary care) |
| Claveau, J., Authier, M., Rodrigues, I., Crevier-Tousignant, M. | Patients’ missed appointments in academic family practices in Quebec | 2020 | Can Fam Physician. 2020;66(5):349-55. | Canada (primary care) |
| Corfield, L., Schizas, A., Williams, A., Noorani, A. | Non-attendance at the colorectal clinic: a prospective audit | 2008 | Ann R Coll Surg Engl. 2008;90(5):377-80 | UK (outpatient - colorectal clinic) |
| Corrigan, P.W., Pickett, S., Schmidt, A., Stellon, E., Hantke, E., Kraus, D., Dubke, R. | Peer navigators to promote engagement of homeless African Americans with serious mental illness in primary care | 2017 | Psychiatry Res. 2017;255:101-3. | USA (primary care) |
| Cosgrove, M.P. | Defaulters in general practice: reasons for default and patterns of attendance | 1990 | Br J Gen Pract. 1990;40(331):50-2. | UK (primary care) |
| Coulter, A., Roberts, S., Dixon, A. | Delivering better services for people with long-term conditions: Building the house of care | 2013 | https://www.kingsfund.org.uk/insight-and-analysis/reports/better-services-people-long-term-conditions | n/a (review paper) |
| Cousins, C., Baxter, J., Javier Vilar, S. R. F. | Non-attendance at hospital clinics for Hepatitis C among intravenous drug users: barriers and potential solutions | 2011 | J Infect. 2011 Dec 1;63(6):e65-6. | UK (outpatient - hepatitis C) |
| Crane, M.A., Cetrano, G., Joly, L.M.A, Coward, S., Daly, B.J.M., Ford, C., Gage, H., Manthorpe, J., Williams, P. | Mapping of specialist primary health care services in England for people who are homeless | 2018 | https://kclpure.kcl.ac.uk/ws/portalfiles/portal/88024144/HEARTH_study_Mapping_SummaryReport_2018.pdf | UK (primary care) |
| Crocker, C., Teehan, M., Ursuliak, Z., Morrison, J., Robertson, N., Alexiadis, M., Tibbo, P. | Patient engagement to early intervention in psychosis services: retrospective analysis of engagement patterns | 2020 | Schizophrenia bulletin, 46(Supplement_1), pp.S129-S130. | Canada (outpatient - mental health) |
| Dantas, L. F., Fleck, J.L., Cyrino O., Fernando L., Hamacher, S. | No-shows in appointment scheduling - a systematic literature review | 2018 | Health Policy. 2018;122(4):412-21. | n/a (review paper) |
| Denneny, E. K., Black, S. E., Bogle, Y., Macavei, V. M., O'Shaughnessy, T. C., White, V. L. C., Kunst, H., Jayasekera, N. P. | Tackling poor attendance to tuberculosis clinic – who, why and what can be done | 2014 | Thorax 2014;69:A210. | UK (outpatient - tuberculosis clinic) |
| Department for Levelling Up, Housing and Communities | Frontline support models for people experiencing multiple disadvantage: A Rapid Evidence Assessment | 2023 | https://assets.publishing.service.gov.uk/media/642af3507de82b000c31350c/Changing_Futures_Evaluation_-_Frontline_support_models_REA.pdf | Various (literature review) |
| Deyo, R. A., Inui, T. S. | Dropouts and broken appointments. A literature review and agenda for future research | 1980 | Med Care. 1980;18(11):1146-57. | n/a (review paper) |
| Dinsdale, P. | Practice nurses reject fines for missed appointments | 2001 | Nurs Stand (through 2013) 2001 Aug;15(49):8. | UK (primary care) |
| Dockery, F., Rajkumar, C., Chapman, C., Bulpitt, C., Nicholl, C. | The effect of reminder calls in reducing non-attendance rates at care of the elderly clinics | 2001 | Postgrad Med J. 2001;77(903):37-9. | UK (outpatient - care for the elderly) |
| DuMontier, C., Rindfleisch, K., Pruszynski, J., Frey, J.J., | A Multi-Method Intervention to Reduce No-Shows in an Urban Residency Clinic | 2013 | Fam Med. 2013;45(9):634-41 | USA (primary care) |
| Dunmore, C., Baldwin, L., Akpan, A. | Social determinants and older people hospital outpatient non-attendance | 2017 | Age Ageing. 2017;46(Supplement 3):iii1 | UK (outpatient - care for the elderly) |
| Dyer, B. T., Swann, F., Kadam, M., Draper, J., Mc Gill, L. A., Kapetanakis, S., Ismail, T., Carr-White, G., Webb, J. | Understanding non-attendance to an inner city tertiary centre heart failure clinic: a pilot project | 2019 | Eur Heart J. 2019;40(Supplement 1):3747 | UK (outpatient - tertiary heart clinic) |
| Eades, C., Alexander, H. | A mixed‐methods exploration of non‐attendance at diabetes appointments using peer researchers | 2019 | Health Expect. 2019;22(6):1260-71 | UK (outpatient - diabetes) |
| Edwards, K. | Identifying Patient Preferences in Appointment Reminders for Adults to Reduce Missed Appointments in an Outpatient Mental Health Clinic: A Quality Improvement Project | 2023 | Masters dissertation, Georgia State University | USA (outpatient - mental health) |
| Ellis, D. A., Jenkins, R. | Weekday Affects Attendance Rate for Medical Appointments: Large-Scale Data Analysis and Implications | 2012 | PLoS One. 2012;7(12):4. | UK (primary care + outpatient) |
| Ellis, D. A., McQueenie, R., McConnachie, A., Wilson, P., Williamson, A. E. | Demographic and practice factors predicting repeated non-attendance in primary care: a national retrospective cohort analysis | 2017 | Lancet Public Health. 2017;2(12):E551-E9. | UK (primary care) |
| Fairhurst, K., Sheikh, A. | Texting appointment reminders to repeated non-attenders in primary care: randomised controlled study | 2008 | Qual Saf Health Care. 2008;17(5):373-6. | UK (primary care) |
| Fee, P. A., Hargan, A. M. | An intervention study to assess the effectiveness of a reminder telephone call in improving patient appointment attendance at a Community Dental Service clinic | 2016 | Community Dent Health. 2016;33(4):239-41 | UK (primary care - dental) |
| Finlayson, S., Boelman, V., Young, R., Kwan, A. | Saving lives, saving money: how homeless health peer advocacy reduces health inequalities | 2015 | https://groundswell.org.uk/wp-content/uploads/2018/10/Groundswell-Saving-Lives-Saving-Money-Full-Report-Web-2016.pdf | UK (primary care + outpatient) |
| Fiori, K.P., Heller, C.G., Rehm, C.D., Parsons, A., Flattau, A., Braganza, S., Lue, K., Lauria, M., Racine, A. | Unmet Social Needs and No-Show Visits in Primary Care in a US Northeastern Urban Health System, 2018–2019 | 2020 | Am J Public Health. 2020;110:S242-S50 | USA (primary care) |
| Franciosi, E.B., Tan, A.J., Kassamali, B., Leonard, N., Zhou, G., Krueger, S., Rashighi, M., Lachance, A. | The Impact of Telehealth Implementation on Underserved Populations and No-Show Rates by Medical Specialty During the COVID-19 Pandemic | 2021 | Telemed J E Health. 2021 Aug 1;27(8):874-80 | USA (multiple) |
| Garuda, S. R., Javalgi, R.G., Talluri, V. S. | Tackling no-show behavior: a market-driven approach | 1998 | Health Mark Q. 1998;15(4):25-44 | n/a (review paper) |
| George, A., Rubin, G. | Non-attendance in general practice: a systematic review and its implications for access to primary health care | 2003 | Fam Pract. 2003;20(2):178-84 | n/a (review paper) |
| Gonzalez, J.S., Peyrot, M., McCarl, L.A., Collins, E.M., Serpa, L., Mimiaga, M.J., Safren, S.A. | Depression and Diabetes Treatment Nonadherence: A Meta-Analysis | 2008 | Diabetes Care. 2008;31(12):2398-403 | n/a (review paper) |
| Gray, S., Wells, K., Moodley, S., Rheuban, K. | Predictors of adolescent telemedicine visit no-shows during the covid-19 pandemic | 2022 | J Adolesc Health. 2022 Apr 1;70(4):S45. | USA (outpatient - adolescent health clinic) |
| Gunner, E., Chandan, S.K., Marwick, S., Saunders, K., Burwood, S., Yahyouche, A., Paudyal, V | Provision and accessibility of primary healthcare services for people who are homeless: a qualitative study of patient perspectives in the UK | 2019 | Br J Gen Pract. 2019;69(685):e526-e36 | UK (primary care) |
| Guo, J.F., Bard, J.J., Morrice, D.R., Jaen, C., Poursani, R. | Offering transportation services to economically disadvantaged patients at a family health center: a case study | 2022 | Health Systems. 2022;11(4):251-75 | USA (primary care) |
| Gupta, A., Wagner, S., Raja, L., Struyven, R., Cortina-Borja, M., Keane, P. A., Huemer, J., Balaskas, K., Sim, D., Rahi, J., Solebo, A., Kang, S. | Determinants of Non-Attendance in Face-to-Face Ophthalmic Clinics Pre- and During the Coronavirus Pandemic | 2022 | 2022 Jun 1;63(7):2813-A0143. | UK (outpatient - ophthalmology) |
| Gurewich, D., Linsky, A.M., Harvey, K.L., Li, M., Griesemer, I., MacLaren, R.Z., Ostrow, R. and Mohr, D. | Relationship Between Unmet Social Needs and Care Access in a Veteran Cohort | 2023 | J Gen Intern Med. 2023;38(SUPPL 3):841-8 | USA (multiple - veterans administration) |
| Gurol-Urganci, I., de Jongh, T., Vodopivec-Jamsek, V., Atun, R., Car, J. | Mobile phone messaging reminders for attendance at healthcare appointments | 2013 | Cochrane Database of Systematic Reviews, 2013(12)(Art. No.: CD007458) | n/a (review paper) |
| Hamilton, W. | General practice non-attendance | 1999 | Br J Gen Pract 1999;49(445):664 | n/a (review paper) |
| Hamilton, W. | Non-attendance in general practice: a questionnaire | 2002 | Prim Health Care Res Dev. 2002;3(4):226-30 | UK (primary care) |
| Harrington, E.E., Reese‐Melancon, C. and Bock, J.E. | Sometimes they show, sometimes they don’t: Appointment attendance as a naturalistic prospective memory task | 2023 | Appl Cogn Psychol. 2023 May;37(3):590-9. | USA - college (non-medical study) |
| Healthwatch | Cost of living: People are increasingly avoiding NHS appointments and prescriptions | 2023 | https://www.healthwatch.co.uk/news/2023-01-09/cost-living-people-are-increasingly-avoiding-nhs-appointments-and-prescriptions | UK (health general) |
| Henry, S. R., Goetz, M. B., Asch, S. M. | The Effect of Automated Telephone Appointment Reminders on HIV Primary Care No-Shows by Veterans | 2012 | J Assoc Nurses AIDS Care, 2012 Sep 1;23(5):409-18. | USA (primary care - HIV) |
| Herber, O.R., Jones, M.C., Smith, K., Johnston, D.W. | ‘Just not for me’ – contributing factors to nonattendance/ noncompletion at phase III cardiac rehabilitation in acute coronary syndrome patients: a qualitative enquiry | 2017 | J Clin Nurs. 2017;26(21-22):3529-42 | UK (outpatient - cardiac rehabilitation) |
| Hermoni, D., Mankuta, D., Reis, S. | Failure to Keep Appointments at a Community Health Centre: Analysis of causes | 1990 | Scandinavian journal of primary health care. 1990 Jan 1;8(2):107-11. | Israel (primary care) |
| Hickmott, S., Stroud, T. | Hidden Dimensions. The complexities of podiatry clinic non attendance of people with diabetes | 2009 | Diabet Med. 2009;26(SUPPL. 1):174. | UK (outpatient - diabetes) |
| Horigan, G., Davies, M., Findlay‐White, F., Chaney, D. and Coates, V. | Reasons why patients referred to diabetes education programmes choose not to attend: a systematic review | 2016 | Diabet Med. 2017;34(1):14-26. | n/a |
| Howarth, A. R., Apea, V., Michie, S., Morris, S., Sachikonye, M., Mercer, C. H., Evans, A., Delpech, V. C., Sabin, C., Burns, F. M. | Associations with sub-optimal clinic attendance and reasons for missed appointments among heterosexual women and men living with HIV in London | 2022 | AIDS Behav. 2022;26(11):3620-9. | UK (outpatient - HIV) |
| Hull, A. M., Alexander, D. A., Morrison, F., McKinnon, J. S. | A waste of time: non-attendance at out-patient clinics in a Scottish NHS Trust | 2002 | Health Bull (Edinb). 2002;60(1):62-9. | UK (outpatient - various) |
| Hussain-Gambles, M., Neal, R. D., Dempsey, O., Lawlor, D. A., Hodgson, J. | Missed appointments in primary care: questionnaire and focus group study of health professionals | 2004 | Br J Gen Pract. 2004;54(499):108-13. | UK (primary care) |
| Inglesfield, J. | Non-attendance and mental health problems in primary care | 1999 | Br J Gen Pract. 1999;49(443):488-9. | UK (primary care) |
| Izard, T. | Managing the Habitual No-Show Patient | 2005 | 2005 Feb;12(2):65-6. | USA (primary care) |
| Jefferson, L., Atkin, K., Sheridan, R., Oliver, S., Macleod, U., Hall, G., Forbes, S., Green, T., Allgar, V., Knapp, P. | Non-attendance at urgent referral appointments for suspected cancer: a qualitative study to gain understanding from patients and GPs | 2019 | Br J Gen Pract. 2019;69(689):E850-E9. | UK (outpatient - cancer referral) |
| Johnson, B. J., Mold, J. W.,Pontious, J. M. | Reduction and Management of No-Shows by Family Medicine Residency Practice Exemplars | 2007 | Annals Family Med. 2007;5(6):534-9 | USA (primary care) |
| Jones, M. C., Smith, K., Herber, O., White, M., Steele, F., & Johnston, D. W. | Intention, beliefs and mood assessed using electronic diaries predicts attendance at cardiac rehabilitation: An observational study | 2018 | Int J Nurs Stud. 2018;88:143-52. | UK (outpatient - cardiac rehabilitation) (Scotland) |
| Kaplan-Lewis, E., Percac-Lima, S. | No-Show to Primary Care Appointments: Why Patients Do Not Come | 2013 | J Prim Care Community Health. 2013;4(4):251-5. | USA (primary care) |
| Kiruparan, P., Kiruparan, N., Debnath, D. | Impact of pre-appointment contact and short message service alerts in reducing ‘Did Not Attend’ (DNA) rate on rapid access new patient breast clinics: a DGH perspective |  | BMC Health Serv Res. 2020;20(1):9. | UK (outpatient - new patient breast clinics) |
| Koester, K.A., Johnson, M.O., Wood, T., Fredericksen, R., Neilands, T.B., Sauceda, J., Crane, H.M., Mugavero, M.J., Christopoulos, K.A. | The influence of the ’good’ patient ideal on engagement in HIV care | 2019 | PLoS One. 2019;14(3). | USA (outpatient - HIV care) |
| Lacy, N. L., Paulman, A.,Reuter, M. D., Lovejoy, B. | Why We Don’t Come: Patient Perceptions on No-Shows | 2004 | Ann Fam Med. 2004;2(6):541-5. | USA (primary care) |
| Lakshminarayana, I. | Measures to improve non attendance rates of community paediatric outpatient clinics | 2016 | Arch Dis Child. 2016;101(Supplement 1):A106 | UK (outpatient - community paediatrics) |
| Lasser, K. E., Mintzer, I. L., Lambert, A., Cabral, H., Bor, D. H. | Missed Appointment Rates in Primary Care: The Importance of Site of Care | 2005 | J Health Care Poor Underserved. 2005;16(3):475-86. | USA (primary care) |
| Lawal, M. O. | Non-attendance in diabetes education centres: perceptions of patients and education providers | 2014 | Diabet Med. 2014;31(SUPPL. 1):102-3. | UK (outpatient - diabetes education) |
| Lawal, M., & Woodman, A. | Socio-demographic Determinants of Attendance in Diabetes Education Centres: A Survey of Patients’ Views | 2021 | EMJ Diabetes. 2021;9(1):102-9. | UK (outpatient - diabetes education) |
| Leavey, G., Vallianatou, C., Johnson-Sabine, E., Rae, S., Gunputh, V. | Psychosocial Barriers to Engagement With an Eating Disorder Service: A Qualitative Analysis of Failure to Attend | 2011 | Eat Disord. 2011;19(5):425-40. | UK (outpatient - eating disorder) |
| Liu, S., Ng, J.K.Y., Moon, E.H., Morgan, D., Woodhouse, N., Agrawal, D., Chan, L., Chhabra, R. | Impact of COVID-19-associated anxiety on the adherence to intravitreal injection in patients with macular diseases a year after the initial outbreak | 2021 | Ther Adv Ophthalmol. 2022:1-12. | UK (outpatient - ophthalmology) |
| Lyon, R., Reeves, P. J. | An investigation into why patients do not attend for out-patient radiology appointments | 2005 | Radiography. 2006;12(4):283-90 | UK (outpatient - radiology) |
| Macharia, W.M. | An overview of interventions to improve compliance with appointment keeping for medical services | 1992 | JAMA. 1992;267(13):1813-7. | n/a (review paper) |
| Maehl, N., Bleckwenn, M., Riedel-Heller, S. G., Mehlhorn, S., Lippmann, S., Deutsch, T., Schrimpf, A. | The Impact of the COVID-19 Pandemic on Avoidance of Health Care, Symptom Severity, and Mental Well-Being in Patients With Coronary Artery Disease | 2021 | Front Med (Lausanne). 2021;8. | Germany (primary care) |
| Magan, T., Kirmani, A., Robertson, M., Mohamed, M., Mann, S. | Non-attendance in the ranibizumab treatment clinic for diabetic macular oedema: rates and reasons | 2014 | Eur J Ophthalmol. 2014;24(3):465-6. | UK (outpatient - ranibizumab treatment clinic) |
| Maggs, C., Langley, C. | Why patients miss primary care appointments: involving patients in research | 2008 | Prim Health Care. 2008;18(2):34-7. | UK (primary care) |
| Mahmood, F. | Exploring reasons for clients’ non-attendance at appointments within a community-based alcohol service: clients’ and practitioners’ perspectives. | 2021 | Doctoral dissertation, Manchester Metropolitan University | UK (outpatient - community-based alcohol services) |
| Margham, T. | Reducing missed appointments in general practice: evaluation of a quality improvement programme in East London. | 2021 | Br J Gen Pract. 2021;71(704):109- | UK (primary care) |
| Marshall, D., Quinn, C., Child, S., Shenton, D., Pooler, J., Forber, S., Byng, R. | What IAPT services can learn from those who do not attend | 2016 | J Ment Health. 2016;25(5):410-5. | UK (outpatient - IAPT) |
| Martin, C., Perfect, T., Mantle, G. | Non-attendance in primary care: the views of patients and practices on its causes, impact and solutions | 2005 | Fam Pract. 2005;22(6):638-43. | UK (primary care) |
| Martin, P.M. | Coroner inquest into 'hospital non-attendance' management in primary care | 2019 | Br J Gen Pract. 2019;69(681):195. | UK (primary care) |
| Martin, S. J., Bassi, S., Dunbar-Rees, R. | Commitments, norms and custard creams - a social influence approach to reducing did not attends (dnas) | 2012 | J R Soc Med. 2012;105(3):101-4. | UK (primary care) |
| Mason, C. | Non-attendance at out-patient clinics: a case study | 1992 | J Adv Nurs. 1992;17(5):554-60. | UK (outpatient - various) |
| Masoud, T., Shah, A. and Joomun, S. | Reducing DNA Rates and Increasing Positive Contacts in an Outpatient Chronic Fatigue Service | 2017 | BMJ Quality Improvement Reports. 2017;6(1). | UK (outpatient - chronic fatigue service) |
| Maughan, D.L., Pearce, M. | Reducing non-attendance rates in community psychiatry: a case for sustainable development? | 2015 | BJPSych International, 2015;12(2):36–9. | UK (outpatient - community psychiatry) (England) |
| Mault, S., McDonough, B. J., Currie, P., Burhan, H. | Reasons proffered for non-attendance at a difficult asthma clinic | 2012 | Thorax. 2012;67(SUPPL. 2):A187. | UK (outpatient - asthma clinic) |
| Mayer, J., Abraham, P., Burhan, H., McDonough, B. J., Mault, S. | The effect of distance from the hospital, public transport availability and socioeconomic deprivation on non-attendance at a difficult asthma clinic | 2013 | Thorax. 2013;68:A198. | UK (outpatient - asthma clinic) |
| McCarthy, L., Parr, S., Green, S, Reeve, K. | Understanding models of support for people facing multiple disadvantage: A Literature Review | 2020 | https://www.shu.ac.uk/centre-regional-economic-social-research/publications/understanding-models-of-support-for-people-facing-multiple-disadvantage-a-literature-review | n/a (review paper) |
| McLean, S. M., Booth, A., Gee, M., Salway, S., Cobb, M., Bhanbhro, S., Nancarrow, S. A. | Appointment reminder systems are effective but not optimal: results of a systematic review and evidence synthesis employing realist principles | 2016 | Patient Prefer Adherence. 2016;10:479-99. | n/a (review paper) |
| McLean, S., Gee, M., Booth, A., Salway, S., Nancarrow, S., Cobb, M., Bhanbhro, S. | Targeting the use of reminders and notifications for uptake by populations (TURNUP): a systematic review and evidence synthesis | 2014 | Health Services and Delivery Research. 2014;2(34). | n/a (review paper) |
| McQueenie, R., Ellis, D. A.,McConnachie, A.,Wilson, P.,Williamson, A. E. | Morbidity, mortality and missed appointments in healthcare: a national retrospective data linkage study | 2019 | BMC Med. 2019;17:9. | UK (primary care) |
| McQueenie, R., Ellis, D.A., Fleming, M., Wilson, P., Williamson, A.E. | Educational associations with missed GP appointments for patients under 35 years old: administrative data linkage study | 2021 | BMC Med. 2021 Sep 27;19(1):219 | UK (primary care) |
| Milne, R.G | Reducing non-attendance at specialist clinics: an evaluation of the effectiveness and cost of patient-focussed booking and SMS reminders at a Scottish health board | 2010 | Int J Consum Stud. 2010;34(5):570-80. | UK (outpatient - various) |
| Minshall, I., Neligan, A. | A review of people who did not attend an epilepsy clinic and their clinical outcomes | 2017 | Seizure. 2017;50:121-4. | UK (primary care - epilepsy). |
| Mitchell, A.J., Selmes, Thomas | A Comparative Survey of Missed Initial and Follow-Up Appointments to Psychiatric Specialties in the United Kingdom | 2007 | Psychiatric services (Washington, DC). 2007;58(6):868-71. | UK (outpatient - psychiatry) |
| Morris, J., Campbell-Richards, D., Wherton, J., Sudra, R., Vijayaraghavan, S., Greenhalgh, T., Collard, A., Byrne, E., O'Shea, T. | Webcam consultations for diabetes: findings from four years of experience in Newham | 2017 | Pract Diabetes. 2017;34(2):45-50. | UK (outpatient - diabetes) |
| Morris, L., Haywood, S. | Why do patient miss appointments? A retrospective population study in paediatric outpatients in a metropolitan hospital | 2014 | Arch Dis Child. 2014;99(SUPPL. 1):A96. | UK (outpatient - paediatrics) |
| Moscrop, A. | Would it be a good idea to charge for missed appointments at the doctors surgery? | 2015 | BMJ Opinion. 2015;351:23-. | n/a (opinion) |
| Moscrop, A., Siskind, D., Stevens, R. | Mental health of young adult patients who do not attend appointments in primary care: a retrospective cohort study | 2012 | Fam Pract. 2012;29(1):24-9. | UK (primary care) |
| Murray, M. | Modernising the NHS - Patient care: access | 2000 | BMJ 2000 Jun 10;320(7249):1594-6 | UK (health general) |
| Nancarrow, S., Bradbury, J., Avila, C. | Factors associated with non-attendance in a general practice super clinic population in regional Australia: A retrospective cohort study | 2014 | Australas Med J. 2014;7(8):323-33 | Australia (primary care) |
| Neal, R. D., Lawlor, D. A., Allgar, V., Colledge, M., Ali, S., Hassey, A., Portz, C., Wilson, A. | Missed appointments in general practice: retrospective data analysis from four practices | 2001 | Br J Gen Pract. 2001;51(471):830-2 | UK (primary care) |
| Neal, R.D., Hussain-Gambles, M., Allgar, V.L., Lawlor, D.A., Dempsey, O. | Reasons for and consequences of missed appointments in general practice in the UK: questionnaire survey and prospective review of medical records | 2005 | BMC Fam Pract. 2005;6:47 | UK (primary care) |
| Nguyen, D.L., Dejesus, R.S., Wieland, ML. | Missed Appointments in Resident Continuity Clinic: Patient Characteristics and Health Care Outcomes | 2011 | J Grad Med Educ. 2011;3(3):350-5 | USA (primary care) |
| NHS England | Approaches to implementing two-way appointment reminders​ | 2023 | NHS England | UK (health general) |
| Ogunyemi, A.O. | Reducing the Prevalence of Missed Primary Care Appointments in Community Health Centers. Capstone Project Paper | 2020 | Doctoral Dissertation, University of Southern California | USA (primary care) |
| Opon, S., Ochieng, T., Wanja M., Njoroge, K.M. | The effect of patient reminders in reducing missed appointment in medical settings: a systematic review | 2020 | PAMJ-One Health, 2(9) | n/a (review paper) |
| Pakhomova, T.E., Nicholson, V., Fischer, M., Ferguson, J., Moore, D.M., Salters, K., Lester, R.T., Kremer, H., Dawydiuk, N., Barrios, R. and Parashar, S. | Exploring Primary Healthcare Experiences and Interest in Mobile Technology Engagement Amongst an Urban Population Experiencing Barriers to Care | 2023 | Qual Health Res. 2023;33(8-9):765-77 | Canada (primary care) |
| Pal, B., Taberner, D. A., Readman, L. P., Jones, P. | Why do outpatients fail to keep their clinic appointments? Results from a survey and recommended remedial actions | 1998 | Int J Clin Pract. 1998;52(6):436-7. | UK (outpatient - various) |
| Parker, M.M., Moffet, H.H., Schillinger, D., Adler, N., Fernandez, A., Ciechanowski, P., Karter, A.J. | Ethnic Differences in Appointment Keeping and Implications for the Patient-centeredmedical Home - Findings from the Diabetes Study of Northern California (DISTANCE) | 2012 | Health Serv Res. 2012;47(2):572-93. | USA (primary care - diabetes) |
| Parkes, T., Matheson, C., Carver, H., Foster, R., Budd, J., Liddell, D., Wallace, J., Pauly, B., Fotopoulou, M., Burley, A., Anderson, I., MacLennan, G. | A peer-delivered intervention to reduce harm and improve the well-being of homeless people with problem substance use: the SHARPS feasibility mixed-methods study | 2022 | Health Technol Assess. 2022;26(14):1-128. | UK (outreach care) |
| Parsons, J., Abel, G., Mounce, L.T., Atherton, H. | The changing face of missed appointments | 2023 | Br J Gen Pract. 2023;73(728):134-5. | UK (general) |
| Parsons, J., Bryce, C., Atherton, H. | Which patients miss appointments with general practice and the reasons why: a systematic review | 2021 | Br J Gen Pract. 2021;71(707):E406-E12. | n/a (review paper) |
| Perron, N.J., Dao, M.D., Kossovsky, M.P., Miserez, V., Chuard, C., Calmy, A., Gaspoz, J-M. | Reduction of missed appointments at an urban primary care clinic: a randomised controlled study | 2010 | BMC Fam Pract. 2010;11. | Switzerland (primary care) |
| Poll, R., Allmark, P., Tod, A. M. | Reasons for missed appointments with a hepatitis C outreach clinic: A qualitative study | 2017 | Int J Drug Policy. 2017;39:130-7. | UK (outpatient - hepatitis C) |
| Practice Nurse (no author named) | Missing appointments increases risk of death | 2019 | Practice Nurse. 2019:49(1) | UK (primary care) |
| Practice Nurse (no author named) | Change booking system to cut DNAs | 2020 | Practice Nurse. 2020:50(10) | UK (primary care) |
| Prentice, P | Missed appointments | 2004 | Practice Management.2004:14(8) | UK (primary care) |
| Prudden, G. | Quality improvement project exploring the factors in non-attendance at an NHS musculoskeletal outpatients department | 2021 | Physiotherapy (United Kingdom). 2021;113(Supplement 1):e151-e2. | UK (outpatient - musculoskeletal) |
| Qin, J., Chan, C.W., Dong, J., Homma, S. and Ye, S. | Telemedicine is associated with reduced socioeconomic disparities in outpatient clinic no-show rates | 2023 | Journal of Telemedicine and Telecare. 2023:0(0) | USA (outpatient - internal medicine) |
| Raja, L., Wagner, S., Struyven, R., Cortina-Borja, M., Keane, P. A., Huemer, J., Balaskas, K., Sim, D., Rahi, J., Solebo, A. L., Kang, S. | Determinants of non-attendance in synchronous teleophthalmology clinics. | 2022 | Investigative Ophthalmology and Visual Science. 2022:63(7):1412-A0108. | UK (outpatient - ophthalmology) |
| Reekie, D., Devlin, H. | Preventing failed appointments in general dental practice: a comparison of reminder methods | 1998 | Br Dent J. 1998;185(9):472-4. | UK (primary care - dental) |
| Revolving Doors Agency | Navigating complexity: learning from Navigators across Birmingham | 2020 | https://www.tnlcommunityfund.org.uk/media/insights/documents/Navigating-Complexity-Learning-from-the-navigators-across-Birmingham-2020.pdf?mtime=20220601114929&focal=none | UK (non-health setting) |
| Roberts, L., Garo-Falides, J., Bowran, H. | Non-attendance in musculoskeletal outpatients: The good, the bad and the ugly | 2015 | Physiotherapy (United Kingdom). 2015;101(SUPPL. 1):eS1289-eS90. | UK (outpatient - musculoskeletal) |
| Robotham, D., Satkunanathan, S., Reynolds, J., Stahl, D., Wykes, T. | Using digital notifications to improve attendance in clinic: systematic review and meta-analysis | 2016 | BMJ Open. 2016;6(10):14. | n/a (review paper) |
| Rose, K.D., Ross, J.S., Horwitz, LI.. | Advanced access scheduling outcomes: a systematic review | 2011 | Archives of Internal Medicine, 2022:171(13) | n/a (review paper) |
| Ross, S. K. | Cancellation and default from appointments in primary care | 1991 | Br J Gen Pract. 1991;41(342):34. | UK (primary care) |
| Rowett, M., Reda, S., Makhoul, S. | Prompts to Encourage Appointment Attendance for People With Serious Mental Illness | 2010 | Schizophr Bull. 2010;36(5):910-1. | n/a (review paper) |
| Royal College of General  Practitioners | Missed GP appointments are frustrating – but there may be underlying reasons why patients don't turn up, says College | 2020 | https://www.rcgp.org.uk/news/missed-gp-appointments | UK (primary care) |
| Royal College of General Practitioners | Charging for missed appointments won’t address intense GP pressures | 2022 | https://www.rcgp.org.uk/news/missed-appointments | UK (primary care) |
| Royal College of General Practitioners | Charging for GP appointments would have the biggest impact on vulnerable patients, says College Chair | 2023 | https://www.rcgp.org.uk/News/GP-appointment-charges-response | UK (primary care) |
| Ruggeri, K., Folke, T., Benzerga, A., Verra, S., Buttner, C., Steinbeck, V., Yee, S., Chaiyachati, K. | Nudging New York: adaptive models and the limits of behavioral interventions to reduce no-shows and health inequalities | 2020 | BMC Health Serv Res. 2020;20(1). | USA (multiple) |
| Samuels, R.C., Ward, V.L., Melvin, P., Macht-Greenberg, M., Wenren, L. M., Yi, J., Massey, G., Cox, J.E. | Missed Appointments: Factors Contributing to High No-Show Rates in an Urban Pediatrics Primary Care Clinic | 2015 | Clin Pediatr. 2015;54(10):976-82. | USA (primary care - peadiatric) |
| Schwebel, F.J., Larimer, M. E. | Using text message reminders in health care services: A narrative literature review | 2018 | Internet Interv. 2018;13:82-104. | n/a (review paper) |
| Shah, S.J., Cronin, P., Hong, C.S., Hwang, A.S., Ashburner, J.M., Bearnot, B.I., Richardson, C.A., Fosburgh, B.W. and Kimball, A.B. | Targeted Reminder Phone Calls to Patients at High Risk of No-Show for Primary Care Appointment: A Randomized Trial | 2016 | J Gen Intern Med. 2016;31(12):1460-6 | USA (primary care) |
| Shahab, I., Meili, R. | Examining non-attendance of doctor’s appointments at a community clinic in Saskatoon | 2019 | Can Fam Physician. 2019;65(6):E264-E8 | Canada (primary care) |
| Sharp, D. J., Hamilton, W. | Non-attendance at general practices and outpatient clinics | 2001 | BMJ (Clinical research ed). 2001;323(7321):1081-2. | UK (primary care + outpatient) |
| Sharp, L., Cotton, S., Thornton, A., Gray, N., Cruickshank, M., Whynes, D., Duncan, I., Hammond, R., Smart, L., Little, J., Tombola Grp | Who defaults from colposcopy? A multi-centre, population-based, prospective cohort study of predictors of non-attendance for follow-up among women with low-grade abnormal cervical cytology | 2012 | European Journal of Obstetrics & Gynecology and Reproductive Biology. 2012;165(2):318-25. | UK (outpatient - colposcopy) |
| Shimotsu, S.,Roehrl, A.,McCarty, M.,Vickery, K.,Guzman-Corrales, L.,Linzer, M.,Garrett, N. | Increased Likelihood of Missed Appointments (“No Shows”) for Racial/Ethnic Minorities in a Safety Net Health System | 2016 | J Prim Care Community Health. 2016;7(1):38-40 | USA (multiple) |
| Simmons, D., Clover, G. | A case control study of diabetic patients who default from primary care in urban New Zealand | 2007 | Diabetes Metab. 2007;33(2):109-13. | New Zealand (primary care - diabetes) |
| Sims, H., Sanghara, H., Hayes, D., Wandiembe, S., Finch, M., Jakobsen, H., Tsakanikos, E., Okocha, C.I. and Kravariti, E., | Text Message Reminders of Appointments: A Pilot Intervention at Four Community Mental Health Clinics in London | 2012 | Psychiatr Serv. 2012 Feb;63(2):161-8 | UK (outpatient - community mental health) |
| Stevenson, J. S. | Appointment systems in general practice: How patients use them | 1967 | BMJ 1967 Jun 6;2(5555):827. | UK (primary care) |
| Sumarsono, A., Case, M., Kassa, S. and Moran, B., | Telehealth as a Tool to Improve Access and Reduce No‑Show Rates in a Large Safety‑Net Population in the USA | 2023 | Bull N Y Acad Med. 2023;100(2):398-407 | USA (various) |
| Sun, C.A., Shenk, Z., Renda, S., Maruthur, N., Zheng, S., Perrin, N., Levin, S. and Han, H.R. | Experiences and Perceptions of Telehealth Visits in Diabetes Care During and After the COVID-19 Pandemic Among Adults With Type 2 Diabetes and Their Providers: Qualitative Study | 2023 | JMIR diabetes. 2023;8:e44283-e | USA (outpatient - diabetes care).` |
| Sun, C-A, Taylor, K., Levin, S., Renda, S.M., Han, H-R. | Factors associated with missed appointments by adults with type 2 diabetes mellitus: a systematic review | 2021 | BMJ Open Diabetes Res Care. 2021;9(1) | n/a (review paper) |
| Tait, J., Noyes, K., Bath, L., Henderson, M., Elleri, D. | Clinic non-attendance, glycaemic control and deprivation score in paediatric and young persons' diabetes clinics in Lothian, Scotland | 2017 | Pediatr Diabetes. 2017;18(Supplement 25):88. | UK (outpatient - paediatric/young persons diabetes clinic) |
| Taylor, B. | Patient use of a mixed appointment system in an urban practice | 1984 | BMJ. 1984;289(6454):1277-8. | UK (primary care) |
| Teo, A.R., Niederhausen, M., Handley, R., Metcalf, E.E., Call, A.A., Jacob, R.L., Zikmund-Fisher, B.J., Dobscha, S.K. and Kaboli, P.J. | Using Nudges to Reduce Missed Appointments in Primary Care and Mental Health: a Pragmatic Trial | 2023 | Journal of General Internal Medicine. 2023;38(SUPPL 3):894-904. | USA (primary care) |
| Thapar, A,. Ghosh, A. | Non-attendance at a psychiatric clinic | 1991 | Psychiatr Bull. 1991;15(4):205-6. | UK (outpatient - psychiatry) |
| Tonnesen, M., Hedeager Momsen, A.M. | Bridging gaps in health? A qualitative study about bridge-building and social inequity in Danish healthcare | 2023 | Int J Qual Stud Health Well-being 2023;18(1):2241235. | Denmark (health general) |
| Traeger, L., O'Cleirigh, C.l., Skeer, M.R., Mayer, K.H., Safren, S.A. | Risk factors for missed HIV primary care visits among men who have sex with men | 2012 | J Behav Med. 2012;35(5):548-56 | USA (primary care - HIV) |
| Ullah, S., Rajan, S., Liu, T., Demagistris, E., Jahrstorfer, R., Anandan, S., Gentile, C., Gill, A. | Why do Patients Miss their Appointments at Primary Care Clinics? | 2018 | J Fam Med Dis Prev. 2018;4(3):1-5. | USA (primary care) |
| Unger, K., Lesiuk, A. and Unger, S. | How do we save £1200 lost to dnas’ per complex paediatric respiratory clinic and protect our most vulnerable patients? | 2023 | Arch Dis Child 2023;108:A441 | UK (outpatient - complex paediatric respiratory clinic) |
| NHS | Identifying causes of patient Did Not Attends (DNAs) | 2023 | NHS England | UK (health general) |
| NHS | Identifying causes of patient Did Not Attends (DNAs) – example script | 2023 | NHS England | UK (health general) |
| van Baar, J. D.,Joosten, H.,Car, J.,Freeman, G. K.,Partridge, M. R.,van Weel, C.,Sheikh, A. | Understanding reasons for asthma outpatient (non)attendance and exploring the role of telephone and e-consulting in facilitating access to care: exploratory qualitative study | 2006 | Qual Saf Health Care. 2006;15(3):191-5. | UK (outpatient - asthma) |
| Vetter, I. | Primary health care for people with multiple and complex needs: what does best practice look like? | 2020 | https://www.bht.org.uk/wp-content/uploads/2021/02/Primary-Healthcare-What-does-best-practice-look-like-May-2020.pdf | UK (primary care) |
| Waller, J., Hodgkin, P. | Defaulters in general practice: who are they and what can be done about them? | 2000 | Fam Pract. 2000;17(3):252-3 | UK (primary care) |
| Wang, Y., Baidoo, F.A. | Design of Integral Reminder for Collaborative Appointment Management | 2017 | 50^th^ Annual Hawaii International Conference on System Sciences (HICSS). 2017:910-9. | n/a (other) |
| Weltermann, B.M., Doost, S.M., Kersting, C., Gesenhues, S. | Hypertension management in primary care: how effective is a telephone recall for patients with low appointment adherence in a practice setting? | 2014 | Wiener klinische Wien Klin Wochenschr. 2014;126(19-20):613-8. | Germany (primary care - hypertension) |
| Wilkinson, M. J. | Effecting change in frequent non-attenders | 1994 | Br J Gen Pract. 1994 May;44(382):233 | UK (primary care) |
| Williamson, A. E., Ellis, D.A., Wilson, P., McQueenie, R., McConnachie, A. | Understanding repeated non-attendance in health services: a pilot analysis of administrative data and full study protocol for a national retrospective cohort | 2017 | BMJ Open. 2017;7(2):11 | UK (primary care) |
| Williamson, A.E., McQueenie, R., Ellis, D.A., McConnachie, A., Wilson, P. | Missingness' in health care: Associations between hospital utilization and missed appointments in general practice. A retrospective cohort study | 2021 | PLoS One. 2021;16(6):e0253163. | UK (primary care) |
| Wilsey, K.L. | Why Patients Miss Appointments at an Integrated Primary Care Clinic | 2020 | Doctoral Dissertation, Antioch University | USA (primary care) |
| WIison, B., Astley, P. | Gatekeepers: Access to Primary Care for those with Multiple Needs | 2016 | Stoke-on-Trent: VOICES, Healthwatch and Expert Citizens CIC | UK (primary care) |
| Wilson, R., Winnard, Y. | Causes, impacts and possible mitigation of non-attendance of appointments within the National Health Service: a literature review | 2022 | J Health Organ Manag. 2022;36(7):892-911. | UK (health general) |
| Winkley, K., Evwierhoma, C., Amiel, S. A., Lempp, H. K., Ismail, K., Forbes, A. | Patient explanations for non-attendance at structured diabetes education sessions for newly diagnosed Type 2 diabetes: a qualitative study | 2015 | Diabet Med. 2015;32(1):120-8. | UK (outpatient - diabetes education) |
| Woodcock, E.W. | Managing your appointment 'no-shows' | 2000 | J Med Pract Manag. 2000;15:284-8. | USA (primary care) |
| Yates, L., Brittleton, L., Bean, N. | An investigation into the factors which inﬂuence attendance rates for psychology appointments in an adult intellectual disability service | 2022 | Adv Ment Health Intellect Disabil. 2022 Aug 31;16(4):216-25 | UK (outpatient - psychology/intellectual disability) |
| Zailinawati, A. H., Ng, C. J., Nik-Sherina, H. | Why do patients with chronic illnesses fail to keep their appointments? A telephone interview | 2006 | Asia-Pac J Public Health. 2006;18(1):10-5. | Malaysia (primary care) |

# Evidence Synthesis and CMO Configurations

## 7.1 Synthesising framework

Fundamental causation is a middle-range theory positing that there is a direct causal flow from socioeconomic status (SES) to health inequalities, sustained by multiple mechanisms. Within this framework, individuals make use of “flexible resources” – including knowledge, money, power, prestige, and beneficial social connections – in their health-promoting actions. Inequalities in the distribution of these resources, and in the distribution of risk and protective factors, contribute to health inequalities – and, we propose, to inequalities of service use (5, 7, 8). Lutfey and Freese (8) extend this model with some additional “metamechanisms” (p.1327), seeking to extend beyond a singular focus on human agency. Among these, “habitus” is of most value here. **Habitus** refers to a person’s way of being, knowing or acting in the world, “internalised structures” (9) influenced by social position and producing an unconscious or semi-conscious sense of orientation, a map guiding routine actions in the world (10, 11).

The candidacy framework proposes that inequalities of service uptake lie in the interaction between the identities and resources of people (‘candidates’) and their (mis)alignment with the structural and cultural qualities of services (2, 12). The framework outlines several domains of candidacy, each in a recursive relationship with the others. **Identification** is the process by which a person comes to identify as a candidate – a person with a need that a service might meet. This includes identifying symptoms and seeking instrumental help for them, as well as perceptions of the suitability and appropriateness of a service. Identification is influenced by past service experiences, by personal, familial and communal beliefs around health and care, and macro-level discourses around health, illness and service use, as each informs the habitus and how a person is oriented towards health and healthcare (13). Considering candidacy as a matter of identity frames the candidacy process as one of seeking recognition as needful, but also as being deserving and a legitimate user of a service.

**Navigation** refers to the process of getting to a service’s point-of-entry by mobilising resources that are unequally distributed, akin to flexible resources of fundamental causation. **Permeability and porosity** refer to service-side dynamics governing access, particularly the degree of alignment between a service’s rules, expectations and pathways and a candidate’s identifications, resources and behaviours (2). Whether and how a candidate can access a service, and how they will be received, depends on their alignment with a service’s image of the “ideal user”, who uses services…

.“…precisely in the way they are intended for precisely the problems providers have identified the services as serving [...] with the exact set of competencies and resources required.”(2, 14)

**Presentation** refers to the process of asserting one’s candidacy as needful and legitimate, while **adjudication** involves the appraisal of that candidacy by providers. In the original framework, adjudication is based on “routine judgements” informed by the heuristics of the provider – their established patterns of sense-making, problem-solving or decision-making as they make both moral and practical judgements of candidacies (2, p. 8). Adjudications result in **offers** which candidates respond to through acceptance, refusal or **resistance**. All of this occurs within **local operating conditions** incorporating the design, resourcing, capacity, staffing arrangements, rules and regulations, “specific, localized cultural, organizational and political contexts” of services (12, p.819).

## 7.2 Navigating and synthesising the evidence.

As the main paper suggests, the evidence base for this study is both flawed and limited, and it is reasonable to ask how a coherent programme theory might be synthesised from literature that appears compromised by significant methodological, theoretical or conceptual difficulties. We accounted for the limitations of the evidence base in several ways. Firstly, we included a range of sources of evidence that a systematic review would likely have excluded for lack of direct relevance to multiple missed appointments in UK primary care. Some papers on single missed appointments may provide insight into multiple missed appointments if the problems they describe could plausibly endure over time. Papers on access or service issues generally might refer to missed appointments as a small part of their overall picture, or their findings overlap with things relevant to or mentioned in missed-appointment research. Papers in other outpatient services, or in other geographical settings, hold relevance where their findings overlap with those found in UK primary care, or they deepen insight into causal mechanisms shared between settings. As such the synthesis, while focused on UK primary care, might provide insights relevant to these other settings.

Realist review uses a combination of inductive, deductive and retroductive reasoning, the latter involving the identification of hidden causal forces and mechanisms through “imaginative leaps” into the space beneath the empirical evidence (15, 16). One might question, for example, why we have discarded forgetting as a sufficiently satisfactory causal explanation despite its often being labelled as the primary cause of missed appointments in survey or questionnaire studies and in prior reviews (17-36), with some going so far as to suggest an “epidemic of forgetfulness ”(17, p.101). This partly reflects our thoughts on the limitations of survey research, but also reflects the realist logic of analysis through which we judged that often forgetting was not a *cause* of missingness but more an *outcome* of another causal mechanism. This is also suggested by the fact that interventions using reminders often report quite modest impacts, or no impacts at all for certain populations, suggesting other mechanisms at play (13, 37-39).

Retroductive analysis supported further strategies for managing limitations. Through corroboration, findings come from inferences drawn from the cumulative weight of related evidence across study types, settings and locations, as well as connection to established theories or explanatory frameworks. For example, the demi-regularities (i.e. outcome patterns) related to the concept of ‘treatment burden’ are the consequence of using retroduction to synthesis an existing theoretical concept with findings from administrative, survey and qualitative studies in multiple settings:

“The number of visits was related to the risk of having a no-show (OR = 1.11, CI 1.07, 1.15). [No-show visits] were also more likely among Veterans having multiple comorbidities (OR = 1.71, CI 1.37–2.15), being in priority group 1 (OR = 1.85, CI 1.34, 2.55) or priority groups 4 and 5 (OR = 1.65, CI 1.19, 2.28).” (40, p.844)

“There are times when I just get so fed up with all the pills and all the doctor’s appointments. I’m guilty. I just walk away. […] I won’t go to doctor’s appointments and I stop taking my medications just because I gotta take a break. I got to. Because I would like just for a little bit to feel like a normal person.” (41, p.8)

Other evidence gains salience precisely because of its idiosyncracy, its ability to answer questions that go unasked in other studies. Consider, for example, the role of stigma or misalignment in Chapman et al (42) that is not accounted for in surveys or questionnaires, or the role of attachments and deeper psychological dynamics in surveys in Ciechanowski et al (43) and qualitative study from Leavey et al (44) - concepts that appear in relatively few studies but which propose plausible, persuasive causal mechanisms.

## 7.3 Synthesised Findings

### 7.3.1 Identification – is healthcare ‘for me’?

One of the few areas of theoretical engagement with missed appointments is around peoples’ perceptions of their health and of services, with papers variously discussing the health belief model (24, 33, 45, 46), the theory of planned behaviour (13, 26, 47, 48), and other approaches focused on patient perception, cognition or behaviour around health and healthcare use (49). These models suggest that attendance behaviour is a function of how people think about health and particularly illness – its causes, severity, consequences, level of threat and susceptibility, whether they can control it, whether appointments are beneficial and their costs manageable**.** In theory, where illness is not seen as concerning and appointments are not perceived to be important or to contribute to improved health, or where their purpose or benefits are unclear, patients are less likely to attend. Conversely, attendance is more likely where patients perceive their health to require urgent or important input and that input is seen as necessary and beneficial – patients may be more likely to remember, to afford the appointment priority among other demands, to use limited resources for attendance, and to persist against other barriers in the candidacy process (13, 41, 46, 50-57).

The evidence in this area is difficult to quantify, often built on survey data or questionnaires where participants suggest symptom improvement or deterioration as causes for missing single appointments (13, 18-25, 27-29, 31, 32, 35, 50, 51, 54, 58-66), or studies exploring the relative influence of measures of perceived appointment value or efficacy (28, 34, 46, 47, 67). These studies often felt like a poor fit for *multiple* missed appointments, with a focus on calculative or instrumental rationality and cost-benefit analyses (68, 69). These ways of thinking were roundly rejected by our stakeholder advisory group, who felt this did not support a deeper or more rounded model of human behaviour. They were also a poor fit for our chosen theoretical frameworks of fundamental causation and candidacy and their concepts of identification and habitus, both of which connect health behaviours to deeper internal psychological processes as well as social, cultural and structural forces that pattern ways of being (2, 5, 7, 12, 13, 68). The umbrella term “not for me” comes from a qualitative study in cardiac rehabilitation non-attendance, and encompassed in a single phrase the enduring sense of distance between how a service operates and the identities of candidates (70).

The additional perspective provided by candidacy integrates health, illness and *identity.* In the health belief model, aspects of identity are stratified into “demographic variables”, “psychological characteristics” and “health motivations”, abstracted from patients’ situated understandings of themselves (71). In our stakeholder advisory group, participants spoke about the need to integrate beliefs about illness with candidates’ deeply held beliefs about themselves as people, and their deep-rooted relationships with health and with care. Participants also noted that a person might hold several beliefs simultaneously and that these beliefs can cohere, interfere, conflict and change over time. Ideas of what constitutes normal health, or the appropriate threshold for intervention speak to a person’s expectations for themselves but also to “norms, values and meaning systems” (13, p.67) within families and wider communities (53, 67, 68, 70, 72-76). Considering this as a matter of identification and habitus supports a deeper understanding of whether patients feel a service is *for them.*

For some patients, missingness results when they do not perceive a health problem as requiring assistance. ‘Situational’ non-attendance research, focused on single missed appointments, shows that some patients do not attend because symptoms have resolved or their health is at a ‘normal’ or manageable level (13, 18, 20, 21, 23-25, 28, 32, 50, 51, 54, 58, 62, 65, 66, 77-80). Some frequent non-attendance can be attributed to patients with no long-term conditions, or who demonstrate better control and fewer complications in their health (22, 81-83). We do not class this is ‘missingness’ as it does not have a negative impact on health or life chances – although these studies may be impacted by issues of data recording or monitoring that may obscure poor health outcomes, and negative outcomes may well emerge in future (75, 81, 84, 85). Among those with long-term conditions (whom evidence suggests are at greater risk of missingness) some patients do not identify as candidates for regular preventive care appointments because they feel well, have no or few complications, feel their condition does not affect their everyday lives or is not a source of concern (13, 28, 34, 47, 59, 81). Some may consider themselves able to manage independently, or with medication alone and no further input required (28, 47, 50, 53, 70, 76, 86). The belief among patients that their health is fine may mask deteriorating health conditions and contribute to the poor outcomes associated with missingness in the future (34, 81).

While greater fear, anxiety and concern about health can support attendance, they can also inhibit identification for care (13, 30, 53, 70, 73, 87-90). Denial is reported as an issue in several studies, with patients seeking safety by not acknowledging their health conditions or pursuing investigations, information, or treatment (28, 53, 68, 73, 74, 86, 91). Some describe not attending because it is likely to increase worry or concern about their health, rather than helping them reduce or manage their concerns (47). Fear or anxiety about procedures, examinations, pain, discomfort or bad news can also make non-attendance protective (13, 30, 42, 47, 54, 58, 66, 73, 87, 89-92). Some health problems are stigmatised or seen as shameful or embarrassing; patients may have internalised this stigma, or may anticipate it from services or social networks (28, 44, 67, 72-76, 86, 93, 94). Patients in Poll et al (73) describe the multiple forms of fear and anxiety inhibiting attendance at a Hepatitis C clinic:

“scared of a positive diagnosis because they believed the infection was not curable, that it resulted in premature death and they would feel ashamed if they were infected because of perceived stigma.” (p.134)

In their study and in others, patients do not attend because of a belief that nothing can be done for them, or that solutions to poor health are not within their power or the power of services, which may be a particular issue for those with multiple conditions, poor control or worsening symptoms (28, 42, 47, 58, 67, 73). Some patients have very low expectations for their health or low self-esteem and self-worth, or may be seeking to harm themselves (40, 57, 64, 70, 87, 91, 95). This may reflect the degree of adversity patients experience in other areas of their lives, given the intersections between non-attendance, poor mental health, multimorbidity and socio-economic marginalisation and deprivation (13, 25, 40, 46, 51-53, 56, 57, 64, 74, 75, 83-85, 87, 91, 93, 96-110). In Jefferson et al (91), one patient with “chronic pain, diabetes, a history of alcoholism, and depression” (p.e853) did not attend for tests because life was so difficult there was no point in attending just to discover another problem. In Herber et al (73) patients with cardiac conditions incorporated their heart problems within existing low expectations for ‘normal’ health, with improvement neither a priority nor perceived as feasible within a service not designed for their needs.

Exposure to multiple, competing urgencies or demands whose perceived impacts are greater may reduce identification of healthcare as a priority issue (27, 28, 40, 47, 56, 57, 63, 70, 72, 73, 87, 91, 105, 109, 111-114). In the context of multiple disadvantage, or of fears and anxieties or stigma and discrimination, many patients only seek out care in circumstances of crisis or urgent need, and may cease attending when crisis has passed (2, 13, 56, 57, 73, 87). All of these elements contribute to the sense that service offers, even services themselves, are “not for me.”

| **Relevant Context-Mechanism-Outcome configurations (CMOCs)** |
| --- |
| CMOC 1: Where patients do not perceive their health/health conditions to be serious (C), they may be less inclined to attend (O) because they do not see appointments as beneficial or necessary (M) or worth the expenditure of resources (M). |
| CMOC 2: Where patients have past experiences of being unable to improve health (for example, because of enduring poor health, complex health/non-health needs, or past experiences of services being unable to help) (C) they may not attend (O) because they feel that improvements are not desirable, possible or within their/service control (M). |
| CMOC 6: Where patients experience a high degree of fear or anxiety around aspects of attending (C), non-attendance may become a protective strategy (O) because the appointment represents a source of threat and non-attendance a route to safety (M). |
| CMOC 3: Where attendance involves acting counter to normative expectations (C), non-attendance may occur (O) because attendance may place someone at risk of exclusion/sanction/stigma within families, peer groups and communities (M). |
| CMOC 14: Where people with access to fewer resources are exposed to a greater number of urgent, competing, or conflicting demands (C) they may be unable to attend (O), because prioritising resources to attend to those demands means they are not available to attend healthcare appointments (M). |

### 7.3.2 Past presentation, adjudication and relational dynamics

Often, issues of candidacy are framed in terms of a patient’s deficits in “health literacy”, framed as a lack of knowledge around health and healthcare impeding candidacy (41, 47, 74, 81, 88, 95, 115). Rather than being purely an issue of patient deficits, some studies suggest ‘literacy’ issues may reflect whether services have communicated with people meaningfully around health and healthcare in their past candidacy experiences (13, 22, 47, 66, 115). Issues of “misalignment”(42, p.1) or “mismatch”(22, p.13) in how services and patients understand the nature of a health problem, the appropriateness of a proposed solution, or expectations for an appointment, can create gaps between what services offer and what people want or need (13, 24, 42, 44, 77, 90, 112). In Chapman et al (42), patients with multiple missed appointments have high levels of mistrust caused by “perceived misdiagnosis or what they viewed as careless treatment plans”:

“[One patient] would cancel appointments or not show up for appointments because she did not want to hear that her physical health problem was instead mental or behavioral. Others described scenarios in which they had received a misdiagnosis that led to unnecessary surgeries, medications, and interventions.” (pp.4-5)

Others have similarly connected persistent non-attendance to disagreement about the nature of the problem, or whether there is a problem at all, with some suggesting particular issues around mental health and substance use (13, 44, 72, 77). Where patients have long-term conditions with minimal everyday impacts there may be misalignment around whether patients, their significant others, and services share an understanding of the rationales for service- or family-initiated appointments, follow-up appointments, or regular preventive care appointments – all typically missed at a higher rate than patient-initiated appointments (13, 18, 22, 24, 35, 39, 41, 51-53, 59, 77, 95, 106, 116-120). A perspective on misalignment shows the role of services here, and whether they communicate around health or appointments in ways that are meaningful, accessible, and resonant with patients’ candidacies (13, 22, 47, 66, 115).

Qualitative studies connect issues of misalignment to the communication and relational dynamics of appointments, an area where statistical findings on missingness can be synthesised with research on the healthcare experiences of groups at greater risk of missed appointments and missingness: patients experiencing homeless, poor mental health and substance use; with poor physical health and multiple long-term health conditions; living in poverty; from migrant and ethnic minority communities; or whose overlapping or intersecting adversities constitute multiple and complex needs (13, 24-28, 39, 40, 42, 51, 54, 64, 67, 72, 75, 78, 83, 91, 92, 95, 97, 103, 109, 110, 113, 121-128). Patients may have experience of there being little space to engage in meaningful communication: short appointments, shallow or cursory assessments, with minimal discussion, and no space for patients’ perspectives or for meaningful discussion (13, 41, 42, 47, 54, 57, 59, 69, 72, 74, 84, 90, 95, 129-131). Many patients experience unaddressed communication barriers, with the common finding that linguistic minorities miss appointments at a higher rate (13, 24, 54, 119, 132-134) coupled with survey, qualitative and review findings suggesting language barriers contribute to missingness (13, 53, 72, 96, 111, 119, 122, 135) In settings where actions have been taken by services to address linguistic or ‘cultural’ barriers, the association may be reduced (20, 51, 79, 81, 119, 122, 136).

Other communication needs include those around cognitive impairment, sensory impairment, poor mental health (depression, anxiety, PTSD), literacy, and the use of inaccessible medical language, all of which can inhibit communication and lead to patients feeling anxious, unsafe, unheard and disempowered in presenting their candidacies (28, 54, 84, 87, 96, 112, 129, 131, 137, 138). Where issues of communication are not addressed, patients may be additionally reliant on people attending with them, which can be challenging for those who are isolated or living alone, can create vulnerabilities around family and community power dynamics, and lead to a loss of ownership where other peoples’ perceptions of necessity, urgency or priority become a determining factor (36, 112, 114, 133, 135, 137). The broader relational impacts of communication dynamics are discussed below (section 7.3.3). Here the focus is on how these unaddressed communication barriers contribute to instrumental issues. Missingness may occur when poor communication environments produce treatment plans or ‘offers’ that do not align with or incorporate candidates’ perspectives, experiences, or life circumstances, or are solely driven by service prerogatives or medicalised models of care **-** leaving patients feeling that they are unlikely to have their needs met in the future (13, 34, 47, 53, 66, 96, 115, 129, 139).

| **Relevant CMOCs** |
| --- |
| CMOC 4: Where services do not communicate about patients’ health in ways that are accessible, meaningful and resonant (C), patients may not attend (O) because they do not perceive benefits (M) and may feel the service is not ‘for them.’ (M). |
| CMOC 7: Where there is misalignment between patients’ wishes, needs and circumstances (candidacies), and what is offered by services (C), non-attendance may occur (O) because patients do not trust the service (M) or feel what the service offers is ‘for them’ (M). |
| CMOC 11: When patients have communication needs which are not addressed at each stage of candidacy (C), they may be less likely to attend (O) because they may feel unheard and may not be able to give/receive information that will make the appointment instrumentally beneficial (M) or they may feel a sense of discrimination, exclusion, neglect or stigma (M). |
| CMOC 12: Where there are unaddressed power disparities in interactions with services (C) patients may mistrust providers (O) because they feel excluded (M), that there is little space for their own involvement in their own care (M), or that services are neglectful, intrusive or coercive (M). |
| CMOC 13: Where services do not take account of patients’ wider circumstances or view patients as people (C), patients may not trust services (O) because they feel labelled or stereotyped (M), may come to believe that what is offered does not fit with their needs or best interests (M). |

### 7.3.3 Presentation, adjudication and stigma

If candidacy is about identities, then adjudications and offers of service access or support are not only instrumentally unhelpful, but represent a threat to or denial of those identities (2). Patients often describe negative relational experiences with services in these terms - that services do not see them as people deserving of care, feeling dismissed or that they are wasting services’ time, or are a burden on the healthcare system (13, 56, 57, 74, 79, 87, 96, 131, 132, 140). While in a prior realist review of missed appointments the causal role of stigma was unclear (13), here it is central as patients describe being labelled, stereotyped, and experiencing “disapproval, rejection, exclusion and discrimination” from powerful actors in services (141, p.367). This might be because of missingness itself; patients describe feeling reprimanded, criticised, judged or shamed because of missed appointments, and staff in many studies frame non-attendance as a problem caused by irrational, chaotic, demanding, rude, lazy, entitled, feckless, and otherwise irresponsible “users and abusers”(142, p.233) of the health system (47, 53, 54, 66, 68, 79, 95, 98, 104, 140, 143-145). This stigma can be internalised as patients see themselves as deviant and solely responsible for non-attendance in some studies (41, 54, 62, 81, 145, 146). “Less-well-retained” participants in Koester et al (41) express particularly strong judgements about “transgressing” from the “good patient […] informed, adherent, well-organized, self-managing, responsible” (p.5).

Stigma may also relate to other aspects of patients’ lives – homelessness, substance use, relationships, poverty, place of residence, gender transitions, or behaviours and ‘lifestyles’ seen to contribute to poor health(19, 41, 42, 44, 53, 57, 74, 87, 93, 96, 105, 112, 124, 129):

“Individuals described a disinterest in being told to change lifestyle habits or having an illness-related appointment be dominated by conversations of weight, diet, and substance use. Similar stories were expressed related to domestic abuse or sexual behaviors. [This] adds to the anxiety of the appointment and results in either less motivation to attend an appointment or non-attendance.”(42, p.4)

Stigma increases the risk of exposure to stressful or threatening encounters, while reducing the influence that people have within those encounters (94). This can manifest where patients describe services as “accusatory” intrusive or interrogatory about their lives, or where patients feel services show no interest in their wider circumstances and rely on labels, stereotypes and judgements (56, 72, 129, 138). The candidacy framework argues that adjudications are made on practitioners’ categorisations, heuristics and “routine judgements”(2, p.8); where there are stigmatising, treatment pathways are closed off and patients’ candidacies are left unresolved (56, 129). Fearing stigmatising interactions, patients may avoid care entirely, or withhold information to present themselves as worthy candidates; in turn treatment pathways may become less relevant to their needs (3, 74).

Relational dynamics are refracted through wider life experiences and circumstances, and some patients may be particularly attuned to or vigilant in responding to problematic relational or power dynamics because of past caring experiences and marginalised social-structural positions (69, 93, 112, 131). Several studies have connected non-attendance and missingness to patient histories of psychological trauma, abuse, neglect, violence or marginalisation and exclusion, or to patients’ attachment styles, which reflect early life experiences of relationships that pattern approaches to caring relationships throughout life (42-44, 67, 112, 135, 137). Ciechanowski et al (43) connect non-attendance to fearful and dismissing attachment styles, as both speak to negative beliefs about dependence on others. Participants in Leavey et al (44, 112) describe mistrust and fears of abandonment and rejection as reasons for non-attendance at an eating disorder service, rooted in their difficult experiences of caring relationships.

Some have suggested expanding the trauma lens to “social trauma” (112, p.16) and the impacts of poverty, racism and social inequalities on relational elements of access (72). For example, high levels of mistrust towards services are reported among racial and ethnic minority communities, as are higher levels of missed appointments in many studies (13, 59, 67, 72, 79, 125). McLean et al (13) use their own realist logic of analysis to connect these finding on attendance to research on general access challenges, suggesting the a combination of poverty and limited resources, barriers related to language and “health literacy”, and “issues of trust, mutual respect and cultural competency” (p.84). Mahmood (72) builds on the work of Memon et al (2016) around mental health services, arguing that racial/ethnic minority communities experience ineffective communication, poor recognition of/response to their needs, power imbalances, and a “lack of understanding of cultural histories, lack of sensitivity, and racial discrimination” (72, p.155) that connect to issues of stigma and exclusion above – issues found in other settings (13, 69, 95, 96). In other studies, minoritized communities are more likely to have negative perceptions of providers communication style (124).

Experiences of stigma, trauma and exclusion may cause mistrust and distrust to become a central part of service interactions and caring relationships or create feelings of threat or vulnerability for patients in these interactions. These relational issues can cause patients to engage in avoidance of services – reactive-avoidance (53, 74, 87, 93) or approach-avoidance (13, 44, 129) are both named in the literature, where service interactions hold the risk of retraumatisation or exposure to unmanageable threat, stress and distress. It is little surprise that patients who miss appointments express little trust or confidence in their providers, or retain strong negative feelings towards that can override even the most urgent health needs (23, 41, 46, 53, 56, 57, 74, 95, 132, 146). We take an intersectional view of these issues, and the ways in which structural dynamics create multiple, reinforcing inequalities or marginalisations around class, age, gender, or other characteristics that manifest in elements of service design and interpersonal interactions project, reinforcing the sense that a service is not a safe, secure space, and not “for me” (96, 112).

Non-attendance generally has been associated with contexts where patients do not consistently see the same clinician or their preferred staff member, and features in qualitative studies where patients describe the role discontinuity in non-attendance or continuity in supporting attendance (18, 22, 42, 51, 52, 57-59, 124, 143, 147). Where patients have no existing working relationship with a practitioner, they may become anxious or more concerned about judgement, stigma or other unpleasant relational experiences (13, 44, 53). They may feel that doctors know nothing about their needs or may be less motivated to help them (34, 54), or have concerns about talking to a stranger about difficult, retraumatising or stigmatising topics (42, 74, 129). Patients may not attend in order to avoid specific staff members because of past negative experiences (41, 72). Consistency and continuity of a positive relationship may reduce anxieties and fears, stigma and judgement, or misalignment rooted in poor communication, and may support patients and practitioners to build trust and an “alliance” (13, p.92) that motivates attendance through feelings of loyalty and a desire to protect a meaningful relationship (13, 26, 34, 46, 53, 54, 72, 79, 117, 148)

| **Proposed CMOCs** |
| --- |
| CMOC 4: Where services do not communicate about patients’ health in ways that are accessible, meaningful and resonant (C), patients may not attend (O) because they do not perceive benefits (M) and may feel the service is not ‘for them.’ (M). |
| CMOC 6: Where patients experience a high degree of fear or anxiety around aspects of attending (C), non-attendance may become a protective strategy (O) because the appointment represents a source of threat and non-attendance a route to safety (M). |
| CMOC 5: Where patients have difficult histories around caring relationships (psychological trauma, attachment issues, ‘relational injuries’) (C), they may not be willing to attend (O) because asking for help is inhibited by mistrust (M), feels unsafe/threatening or antithetical to their core relational beliefs (M) or they may be concerned that they will struggle to manage interpersonal elements of appointments (M). |
| CMOC 8: When patients anticipate negative interactions with services (hostility, judgement, neglect, disrespect, stigma) (C), attendance is made difficult (O) because the appointment represents a source of threat and non-attendance a route to safety (M) . |
| CMOC 9: Service responses to non-attendance that are punitive, sermonising or stigmatising (C) make the service unsafe, unwelcoming and unhelpful (M) leading to patients not attending (O). |
| CMOC 10: When patients have a background of experienced, internalised and anticipated stigma or discrimination around their identities, lifestyles or health (C) they may be less willing to attend (O) because: they do not feel worthy or deserving of help and support (M); may want to avoid thinking about their stigmatised health condition (M); may anticipate further stigma at the service (M) and thus appointments may be perceived as unthreatening/unsafe (M). |
| CMOC 12: Where there are unaddressed power disparities in interactions with services (C) patients may mistrust providers (O) because they feel excluded (M), that there is little space for their own involvement in their own care (M), or services are neglectful, intrusive or coercive (M). |
| CMOC 13: Where services do not take account of patients’ wider circumstances or view patients as people (C), patients may not trust services (O) because they feel labelled or stereotyped (M), may come to believe that what is offered does not fit with their needs or best interests (M). |

### 7.3.4 Competing demands, competing candidacies and limited resources

Competing demands, priorities or urgencies are used here as a catch-all term for a broad range of phenomena identified in the literature under different names – things like “family and work commitments” (13, p.73), “intervening life problems” (149, p.103), “lack of availability” and “not a priority” (111, p.A411), or “unexpected logistical challenges” (62 p.37,). Related terms appear throughout the survey literature and reviews of missed appointment research (18-21, 23-26, 32, 36, 44, 58, 76, 86, 90, 150, 151). Similar general issues appear in missingness-specific studies, accounting for 43% of responses in Boshers et al’s (146) study, while in Dumontier et al (113) “personal and family issues” (p.635) and “managing the complexity of family, money, and emotional and physical health” (p.639) are evident among their “no-show” cohort. Evidence shows that even in circumstances of significant health need, or where patients do consider attendance important, other urgencies or priorities may take precedence and that candidacies may be inhibited by the “concrete, situated contingencies [and] practical demands of daily living ”(13, p.9; 47, 91). The cumulative evidence around competing demands suggests that patients may forget, lose track of appointments among other urgencies, or may prioritise their resources for other issues experienced as more important. Identification is impacted by how people conceive of or prioritise different demands, according to personal, deeply-held beliefs about how to prioritise their health or their resources, as well as social and cultural patterning of habitus around how people manage (or are expected to manage) their lives (5, 7, 10, 13, 68, 69, 95). The synthesised evidence suggests that those experiencing missingness are positioned in more precarious circumstances and exposed to disruptive forces, while having access to fewer protective or buffering resources to mitigate them by virtue of their socioeconomic position, producing more competing urgencies (7, 94). Some of these urgencies become “competing candidacies” (152, p.49), as patients have to navigate multiple candidacy processes, while having access to fewer resources to do so successfully (7, 94, 152).

The umbrella term “competing demands” contains a multitude of circumstances and possibilities (24, 44, 73), of which we have highlighted four. The first is treatment burden, where the workload of managing healthcare might overwhelm the limited or precarious material, emotional, psychological and social resources of patients (49, 99, 153). The well-established link between missingness and multiple long-term health conditions suggests that such patients have a large number of appointments to attend, and evidence shows that patients who miss multiple appointments often also *have* more appointments (22, 24, 28, 33, 40, 49, 54, 64, 83, 92, 99, 100, 102, 103, 107, 109, 113, 117, 154). Patients talk about not attending because of multiple appointments causing forgetfulness or confusion (22, 73, 91, 114, 129), and having to prioritise between different appointments and manage time-conflicts across parts of the health service (20, 30, 32, 47, 50, 66, 72, 73, 139, 145, 146). Links between missingness, poverty and appointment frequency suggest a financial component to managing the logistics and practicalities of competing candidacies and other demands (83, 97, 98, 110). Treatment burdens are also subjective and experiential. Papers on long-term conditions describe how burdens are made to feel less manageable by worsening symptoms, reduced energy and low mood, social isolation, negative service interactions, and unrealistic or disproportionate treatment plans, and patients can feel overwhelmed, burnt out or drained by the requirements of managing their health (28, 41, 47-49, 53, 54, 67, 72, 74, 84, 86, 131). Patients in these circumstances may have a higher degree of “emotional and physical vulnerability” and mistrust (41, p.7), lower “resiliency”(42, p.4) or “activation”(100, p.990) as both cause and consequence of the burdens of treatment and ill-health. Patients with HIV in Koester et al (41) describe non-attendance as part of their need to “take a break” from burdens of treatment and of having the disease:

“I just get so fed up with all the pills and all the doctor’s appointments. I’m guilty. I just walk away. […] I won’t go to doctor’s appointments and I stop taking my medications just because I gotta take a break. […] I would like just for a little bit to feel like a normal person.” (p.8)

As with “health literacy”, words like activation and resilience can focus on patient deficits as the problem to the exclusion of service and structural contributors to treatment burden, such as the unequal distribution of resources to manage these burdens, or the role of service design and dynamics in their creation. Fragmentation between services, or the restrictions of short, single-issue appointments in primary care, mean that patients with multiple or more complex needs have to identify, navigate and present across multiple appointments, sometimes across multiple services, in different locations (47, 53, 57, 72, 74, 95, 112, 115, 129, 131, 137, 145). The communication issues above may also contribute – experiences of stigma and exclusion increasing burdens on psychological and emotional resources, or a lack of space for patient perspectives resulting in burdensome treatment plans.

The second domain of competing demands relates to work and money. Employment issues feature heavily in several studies across multiple settings and locations on non-attendance generally (22, 23, 28, 36, 42, 47, 53, 54, 58, 59, 63, 65, 70, 73, 76, 77, 86, 91, 130, 133, 139, 140, 145, 146, 149). Patients in qualitative studies describe difficulties negotiating inflexible work arrangements, a lack of understanding from employers, and concerns about losing wages – all of which may be worsened by treatment burden and the volume of appointments (42, 54, 63, 73, 76, 91, 133, 155). This may account for part of the intersection between missingness and poverty, where patients in low-paid or hourly roles may have less flexibility and lose money to attend(13, 63, 73, 96, 101, 150, 156, 157), making missed appointments a “necessary sacrifice or unfortunate reality”(42, p.6). In other studies, patients describe having to attend benefits appointments or experiencing difficulties with benefits changes or problems – a further intersection between exposure to competing demands and limited financial resources (72, 73, 114).

The third domain relates to caring responsibilities and social connection. Many patients may have to balance their own health with the needs of other family members or the demands of family life (13, 28, 33, 51, 53, 58, 59, 73, 79, 86, 88, 91, 114, 137, 139, 145, 146, 150). Norms and expectations are influential in whether people feel they can prioritise their health over the needs of others (13, 28, 145). Some patients may not be able to draw on family or social networks to support with care, to pay for childcare, or to take family members to appointments (54, 88, 133). Stigma plays a role when patients do not want to tell employers, friends, family members or other services about their health conditions in order to secure support with attendance (28, 73, 76). Education and social lives may also act as competing issues or priorities, and again the burden of multiple appointments likely plays a role (54, 58, 59, 62, 72, 73, 145, 146).

The final domain of competing demands relates to peoples’ exposure to forms of marginalisation that create urgent, immediate demands about meeting basic living needs. Correlative research oriented around “social needs” shows that patients who have a greater number of unmet needs (e.g. housing needs, food insecurity, financial needs) are more likely to miss appointments (40, 109). Concepts like “additional complex needs” (72, p.76) or “severe and multiple disadvantage” (112, p.4) suggest the presence of overlapping, interconnected issues that might create competing candidacies and reduce the relative significance of a health appointment (27, 28, 70, 105, 113, 125, 129, 137). Many use the language of “chaos”, a phrase containing stigmatising judgements about patients’ perceived failures to adequately organise, structure or prioritise their everyday lives in a rational manner (13, 26, 52, 64, 72, 79, 91, 96, 98, 104, 112, 137, 140, 142). Several papers here suggest that what appears chaotic or irrational may in fact be well organised but oriented towards meeting other needs and priorities, in a context where precarity and unequal exposure to disruptive forces cause recurring crises (41, 57, 63, 68, 73). Healthcare might be less of a priority than finding a safe place to stay, seeking basic essentials, seeking drugs or alcohol, or candidacy processes for other crucial services (28, 56, 57, 72, 73, 87, 105, 114). As with treatment burden, multiple, urgent demands may make attendance less practically and emotionally or psychologically attainable, and patients may become overwhelmed or disorientated (22, 24, 63):

“You don’t know where you are from day to day [or] from moment to moment, really. So you don’t know when you’re going to wake up in here […] By the time you’ve sorted yourself out you might have missed that appointment.” (87, p.10)

Complex non-medical needs contribute to poor health and to patients’ capacity to comply with treatment, but may be unacknowledged or unaddressed by services, beyond their remit, lowering the perceived value of attending and contributing to frustration, mistrust and low confidence in service offers (34, 44, 53, 55-57, 74, 91, 96). As outlined under “presentation, adjudication and stigma” (7.3.3) services may deny access to certain types of care where they don’t think patients are ‘right’ for it (2, 56, 96, 129). Health services are also designed to promote particular forms of ‘engagement’ that may be a particularly poor fit for patients in these circumstances, explored further below (section 7.3.5).

| **Proposed CMOCs** |
| --- |
| CMOC 14: Where people with access to fewer resources are exposed to a greater number of urgent, competing, or conflicting demands (C) they may be unable to attend (O), because prioritising resources to attend to those demands means they are not available to attend healthcare appointments (M). |
| CMOC 15: Where patients have several health conditions or complex needs (C) and fewer flexible resources (C) they may be less likely to attend appointments (O) because “treatment burden” exhausts or overwhelms their resources (M). |
| CMOC 16: Patients who work in low-paid, inflexible, casual, or hourly-paid employment (C) or who have multiple appointments (C) may not be able to attend (O) because they cannot get time away from work (M), or because taking time off has costs that they cannot bear (M). |
| CMOC 17: Patients who have caring responsibilities (C) may struggle to attend scheduled appointments (O) because they prioritise using their resources to meet the needs/demands/urgencies of the people they care for (M). |
| CMOC 3: Where attendance involves acting counter to normative expectations (C), non-attendance may occur (O) because attendance may place someone at risk of exclusion/sanction/stigma within families, peer groups and communities (M). |
| CMOC 2: Where patients have past experiences of being unable to improve health (for example, because of enduring poor health, complex health/non-health needs, or past experiences of services being unable to help) (C) they may not attend (O) because they feel that improvements are not desirable, possible or within their/service control (M). |
| CMOC 27: When patients live in poverty (C), attendance may be made practically more difficult (O) by a lack of money for transport (M) or to meet other competing demands (M). |

### 7.3.5 Permeability and ‘ease of access’

Permeability and porosity refer to the rules of service access and service use, and particularly the degree of “alignment” or “fit” between a service’s rules, expectations and pathways and a candidate’s identifications, resources and behaviours (2). Misalignment between patient habitus and resources and the permeability of services is evident throughout the missed appointments literature as well as the wider literature on inequalities of access. Many patients may already feel excluded from care because they have been refused registration or access - for lacking a fixed address, because of migration status, substance use, prior missed appointments, or because of service perceptions that they are challenging or difficult – while others may have been reprimanded or sanctioned for missed appointments or other behaviours (e.g through fines, deregistration, or threats thereof) (39, 93, 96, 104, 112, 129, 158). Once a patient is registered, appointment systems might be a poor ‘fit’ for some patients and their resources:

“[Appointments systems] are a threat to permeability by socio-economically disadvantaged people because they require resources and competencies (including stable addresses, being able to read, and being able to present in particular places at particular times).” (2, p.8)

Systems that require patients to make appointments exclusively by phone, or to call at specific times of the day, can be a poor fit with patterns of patients’ demands or their access to phones and phone credit, and patients who miss appointments report long waits on hold and difficulties fulfilling service expectations in making appointments (13, 25, 57, 58, 63, 73, 90, 131, 159). “Friction”(140, p.642) at this stage has been connected to missed appointments, with patients in several studies describing reception staff being difficult, interfering, overreaching or hindering access to care (19, 57, 93, 140, 156). Patients may feel that staff do not understand or account for their specific needs or circumstances in appointment-making, and describe a lack of empathy or a sense of judgement, hostility or stigma akin to that outlined above (13, 57, 87, 93). Indeed, several studies have found stigmatising and negative attitudes among staff towards patients experiencing missingness (66, 69, 79, 95, 98, 104, 140, 142-144). Martin et al (140) suggest a feedback loop, where patients do not attend because of difficult interactions in appointment-making, and staff attitudes towards them worsen, increasing the likelihood of future difficulties. Recent moves towards “total triage” have not been assessed as part of missed appointment research but may introduce further impermeable layers to the appointment-making process (160).

Within a gatekeeper-led appointment system, patients may experience difficulties in arranging appointments that are timely, convenient, and suitable for their needs. The link between greater time-to-appointment and missed appointments has been found consistently across settings and locations in 40 years of studies and has been described in reviews as “the most important predictor” (103, p.416) of non-attendance (13, 18, 25, 26, 39, 44, 46, 51-53, 57, 62, 72, 87, 96-98, 103, 104, 106, 117, 132, 143, 147, 157, 161). In studies on multiple missed appointments this also appears to have a degree of relevance (97, 113, 125). Delays influence missingness in several ways. If patients perceive a need to be urgent or are experiencing crisis but offered appointments far in the future, they may seek care elsewhere or may not return, and this may exacerbate feelings of disrespect, neglect or exclusion (13, 39, 57, 90, 130, 156). As noted in the section “identification – is healthcare ‘for me’?” (7.3.1) identifications change over time, as do physical and mental health symptoms, motivations or beliefs, material circumstances and the constellation of competing demands around a patient, and they may simply forget as time passes or other demands emerge (13, 42, 44, 46, 58, 64, 88, 90, 92, 113, 125, 162). There may be a temporal “window of opportunity” (13, p.103) caused by the alignment of factors enabling identification, navigation and presentation; if appointments fall outside this window, they are less likely to be attended. This includes appointments offered on short notice, which can make navigation of competing demands difficult for those who require notice to get time off work, to arrange transport or otherwise plan their attendance, or may require balancing urgency with choice or continuity of practitioner (13, 17, 24, 26, 32, 33, 42, 54, 77, 106, 113, 118, 124, 139). When patients arrive for their appointments, further delay may occur through long periods in waiting rooms. Some patients find waiting rooms to be unsafe, stressful and anxiety-inducing or spaces of stigma and judgement, or they may have feelings of neglect or lack of care caused or exacerbated by waiting (34, 53, 54, 57, 58, 66, 69, 90, 95, 104). Patients may have other demands to attend to so long waits (or anticipated long waits) or unexpectedly long appointments can complicate planning (42, 54, 62). When these waits end with short, cursory or difficult appointments, people may feel the time and resources spent waiting may have been wasted and their time has not been respected (54, 69, 95). These may account in part for finding that same-day and walk-in appointments, while typically missed at a lower rate, are still missed (21, 43, 117, 122, 124, 158).

Competing demands, often framed as a patient-side issue, can here be reconceptualised as a matter of permeability and the degree of (in)convenience or (in)flexibility offered by services (13, 33, 34, 44, 47, 52-54, 58, 62, 101, 114, 130, 145, 163). Systems may not allow patients to choose appointments that are convenient or suitable to them – whether because of competing demands, or the rhythms and patterns of their lives, or because appointments are only available during narrow daytime or weekday hours (13, 33, 34, 44, 46, 52, 53, 58, 63, 79, 96, 101, 130, 131, 139, 140, 163). Given the significance of relational and communication dynamics, where appointment systems do not allow for patient choice of practitioner, for longer appointments, or for communication support, missed appointments may be more likely. Where patients have several complicated needs to discuss, they are a poor fit for a primary care system designed around short, single-issue appointments and or services built around eligibility criteria that their problems do not neatly fit within (56, 87, 95, 137, 138). Home visits were only mentioned in one study, where many patients who were too unwell to attend did not think they had merited a visit (64). Getting a convenient appointment, with a preferred practitioner, in the right location, requires negotiation that may be harder for some patients due to a combination of low confidence, unaddressed communication barriers, gatekeeping behaviours and rigid appointment systems (13, 79, 91, 96, 101, 111, 131, 134, 156, 164). Difficulties in managing the gatekeeping systems of primary care or other health services may contribute to patients feeling the lack of confidence or skill (or “activation”) in navigating the structures or expectations of services reported above (74, 87, 100).

A further permeability issue relates to administrative error and miscommunication, one of the most common causes of non-attendance in survey studies across health settings (13, 18-20, 22, 25, 51-54, 62, 65, 139, 145). These include patients not receiving notification of appointments; receiving them after appointments were due to take place; on such short notice that they were unable to arrange attendance; or simply receiving the wrong details, undermining attendance but also trust and confidence in the system (29-33, 35, 42, 51, 54, 60, 61, 65, 66, 80, 91, 93, 135, 139, 151). Miscommunications and misunderstandings may be an artefact of inadequate IT or booking systems, but may also speak to practices failing to account for patients’ communication needs, such as sending appointment letters to patients with no fixed address, relying on phone systems or text reminders for patients without phones, or single-language written communications to those who might struggle to read and understand them (91, 93, 114, 151). In many studies patients describe being unable to make contact with services to cancel or rearrange appointments, or cancelling but not having this recorded on practice systems (18, 20, 31, 52, 54, 58, 60, 62, 80, 91, 111, 116, 135, 139, 140).

| **Proposed CMOCs** |
| --- |
| CMOC 7: Where there is misalignment between patients’ wishes, needs and circumstances (candidacies), and what is offered by services (C), non-attendance may occur (O) because patients do not trust the service (M) or feel what the service offers is ‘for them’ (M). |
| CMOC 8: When patients anticipate negative interactions with services (hostility, judgement, neglect, disrespect, stigma) (C), attendance is made difficult (O) because the appointment represents a source of threat and non-attendance a route to safety (M). |
| CMOC 11: When patients have communication needs which are not addressed at each stage of candidacy (C), they may be less likely to attend (O) because they may feel unheard and may not be able to give/receive information that will make the appointment instrumentally beneficial (M) or they may feel a sense of discrimination, exclusion, neglect or stigma (M). |
| CMOC 12: Where there are unaddressed power disparities in interactions with services (C) patients may mistrust providers (O) because they feel excluded (M), that there is little space for their own involvement in their own care (M), or that services are neglectful, intrusive or coercive (M). |
| CMOC 20: Where practices ask patients to see practitioners that they do not choose or have no relationship with (C) patients may be: less willing to attend (O) because of their fears and concerns about seeing someone they do not know or trust (M); because the practitioner knows less about their lives/circumstances/wishes/needs and therefore their advice/support is perceived to be less beneficial (M) or communicating with them is more difficult (M). |
| CMOC 21: When practices or services offer less control, choice or flexibility over the day and time of an appointment to patients (C), they are more likely to miss it (O) because it may conflict with other urgent demands (M). |
| CMOC 22: Where there are delays between a person making an appointment and the appointment itself (C), patients may be less likely to attend (O) because: their symptoms might improve and they feel the appointment is no longer necessary (M); they may worsen and attendance becomes practically impossible (M); they may be more likely to forget; other competing demands may emerge or take priority (M); fears or anxieties may increase; patients may feel disrespected, neglected or unwanted (M). |
| CMOC 23: Where patients are offered appointments at very short notice (C), attendance may be prevented (O) because they cannot negotiate their competing demands (M) or access the resources they need to facilitate attendance (M). |
| CMOC 24: When there are administrative errors with appointment scheduling and/or communication (C) patients do not attend (M) because they are given the wrong details for their appointments, or never receive those details and so are not aware of when they are expected (M). |
| CMOC 25: If services do not have systems for patients to contact them to cancel or rearrange, or these systems are burdensome (C), patients may miss appointments (O) because they are unable to cancel or amend appointments. |
| CMOC 29: Where physical spaces are not welcoming or comfortable (C), patients may not feel comfortable attending (O) because they do not feel safe, secure or welcome (M). |
| CMOC 13: Where services do not take account of patients’ wider circumstances or view patients as people (C), patients may not trust services (O) because they feel labelled or stereotyped (M), may come to believe that what is offered does not fit with their needs or best interests (M). |

### 7.3.6 Navigation and Presentation - Forgetting

The issue of forgetting features prominently in surveys and questionnaires on missed appointments across service contexts and locations (18-23, 27-36), and thus also in reviews (24-26, 39). The abundance of research flagging forgetting as a problem has caused some authors to cite an “epidemic of forgetfulness” on the part of patients (17, p.101) that underpins a significant interventional literature on reminders (13, 37, 38, 45, 60, 80, 89, 120, 147, 154, 158, 159, 163, 165-180). Some cautionary points are necessary around this literature. In surveys and questionnaires, forgetting may be a “smoke screen”(13, p.103) for more complex issues or act as protection against stigma or judgement (13, 37-39). Forgetting may link to dementia, brain injury, cognitive impairment, poor mental health, PTSD or other influences on prospective memory (13, 43, 48, 67, 72, 83, 92, 101, 104, 129, 135, 137, 169) and extends beyond just forgetting the date and time of an appointment into forgetting what it is for, where it is or how to get there safely (“wayfinding”(137, p.5)). Wilson and Astley (93) provide insight into the complexity of forgetting:

“[Tom] developed an opiate dependency, alcohol dependency, severe anxiety, memory lapses and sometimes poor cognition and confusion. Tom also suffers from epilepsy which is exacerbated by his alcohol dependency. […] His memory difficulties and poor physical health can leave him struggling to remember appointments, which can result in missed attendances. When Tom tries to book appointments again, he is met with resistance, reminded of his past missed attendances with no constructive solution.” (p.22)

The absence of this extra context in prior literature on forgetting may reflect many of the issues in the overview of the evidence base provided in the main paper, including the reliance on surveys and questionnaire and the challenges of sampling and recruitment that may exclude patients with these complex needs from research. Forgetting might also be a consequence of competing demands and urgencies (section 7.3.4); to limited networks of support where patients rely on other people to help them remember (135, 181); and to service issues including the absence of or issues with reminder systems (42, 63); delays to appointments (section 7.3.5); or misalignments around the purpose or significance of an appointment (section 7.3.2). As such, we view forgetting less as a cause of missingness and more an *outcome* of a range of context-mechanism interactions. This may account for the modest or mixed evidence for reminders, particularly in circumstances related to missingness, as they leave many of these issues unaddressed (13, 37, 38, 147).

| **Relevant CMOCs** |
| --- |
| CMOC 26: When people have illnesses or conditions that affect cognition or memory (C), they may forget to attend appointments (O), struggle with logistics (O) or feel unsafe or anxious in managing the demands of appointment attendance (O) because of their cognitive difficulties (M). |
| CMOC 22: Where there are delays between a person making an appointment and the appointment itself (C), patients may be less likely to attend (O) because: their symptoms might improve and they feel the appointment is no longer necessary (M); they may worsen and attendance becomes practically impossible (M); they may be more likely to forget; other competing demands may emerge or take priority (M); fears or anxieties may increase; patients may feel disrespected, neglected or unwanted (M). |
| CMOC 18: When patients are isolated/lack social support (C), they may be less likely to attend (O) because they do not have people to help with practicalities of attendance (M), to support with managing competing demands (M), to overcome fears and anxieties around attending (M) or to facilitate communication (M) |
| CMOC 19: When patients are reliant on other people to help them attend (C), their non-attendance (O) may be connected to the other person’s availability, and the influence of other demands on them (M); the other person’s perception of the value of the appointment (M); the other person’s resources (M). |
| CMOC 14: Where people with access to fewer resources are exposed to a greater number of urgent, competing, or conflicting demands (C) they may be unable to attend (O), because prioritising resources to attend to those demands means they are not available to attend healthcare appointments (M). |
| CMOC 1: Where patients do not perceive their health/health conditions to be serious (C), they may be less inclined to attend (O) because they do not see appointments as beneficial or necessary (M) or worth the expenditure of resources (M). |

### 7.3.7 Navigation and Presentation: Getting There

Some patients may consistently struggle to travel to their appointments. Transport and logistics feature in literature reviews on non-attendance (13, 24-26, 39, 51, 103, 132, 161) and in survey, questionnaire, and interview studies with varying degrees of prominence influenced by setting, patient population and the relative prevalence of other blockers or barriers (18-21, 23, 57-60, 81, 91, 151, 182). In some studies on multiple missed appointments it is much less prominent than other barriers (28, 90, 119), and greater distance between home and healthcare settings has been associated with *higher* rates of attendance (13, 92, 97). Contextual factors – urban settings, the relative affluence of patient populations, or hospital settings that are likely further away than general practices – might influence these findings (22, 97, 119). In other studies, transport is a prominent part of missingness (27, 40, 42, 86, 111, 114, 182). Transport is included alongside other “societal barriers” as a problem for 30% of patients missing more than half of their appointments in Boshers et al (146, p.e2047). Using public transport is associated with missed appointments, and several papers discuss costs, unreliability, and the greater time and effort required (21, 42, 47, 52, 54, 63, 72, 77, 86, 114, 145, 182, 183). Car transport brings its own issues in terms of fuel costs, parking issues, weather, traffic and a range of other issues (33, 54, 61, 86, 114, 145). Where patients have limited financial resources, these costs may be unmanageable or patients may have competing priorities for their money, particularly for those who have more appointments (27, 40, 42, 54, 59, 70, 72-74, 87, 88, 91, 111, 129, 135, 145, 183). Where health services provide assistance with transport, patients report issues with inaccessible booking systems, lack of sufficient notice, errors, transport failures, and issues with reimbursement (32, 33, 42, 60, 66, 73, 91, 113, 145).

Patients’ mobilities might be impacted by health conditions, disabilities or worsening symptoms (36, 47, 61, 72-74, 86, 87, 129, 130, 145, 155, 182, 184) and in many studies, patients describe not attending because they feel too unwell (13, 18-23, 25, 27-32, 35, 50, 51, 54, 58, 60-66, 79, 126, 145). In missingness, we might differentiate “short illness” – a situational barrier – from “long illness” which might more consistently or frequently impede attendance (73, p.134). This may be an issue for patients with multiple, long-term health conditions or more complex health needs – a group much more likely to be missing (13, 25, 26, 33, 40, 53, 72, 83, 113, 125) – and may contribute to the connection between non-attendance and indicators of poor illness control parameters or deteriorating health, including hospitalisation (28, 49, 58, 99, 124, 132, 134, 185, 186). This incorporates both mental and physical health. Patients in several studies describe agoraphobia or significant anxieties about travel (44, 63, 73, 129, 135) In Tonnesen and Momsen (137) patients struggle with “wayfinding” because of mental health or cognitive difficulties:

“I suffer from anxiety – to a point where I lose awareness of where I am heading. I panic – where do I go and how.” (p.5)

Fatigue, low mood, low energy, crises, breakdowns and the sense of being overwhelmed may impact on whether people feel able to attend, and may particularly impact patients with multiple health conditions, mental health conditions, or who are exposed to multiple stressors (28, 42-44, 47-49, 63, 67, 73, 75, 84, 92, 187).

Further issues include difficulties travelling long distances, particularly difficult for patients for whom transport is not accessible or travelling is distressing and difficult (13, 54, 145). In several studies transport and travel are described in terms of threat and safety - due to frailty (76); disabilities (130, 145); travelling through areas where abusive partners live (130); or where drug-taking acquaintances are (73); and concerns about contracting diseases such as COVID-19 (36). Treatment burden is again an issue – if every journey is difficult, complex or costly, and if patients have to make multiple or frequent journeys, missingness might result. In many studies patients describe the role of family members or friends helping with the practical or emotional burdens of travel (21, 42, 63, 73, 135, 184). Reliance on other people for support with travel and transport can foster conditions for missingness. Friends or family may be unavailable, unable or unwilling to assist; people may feel they are burdening others with their own demands; or they may have limited support networks, difficult relationships with family, or worry about stigma (21, 40, 42, 47, 54, 62, 63, 72, 73, 96, 114, 135, 137, 184).

| **Relevant CMOCs** |
| --- |
| CMOC 27: When patients live in poverty (C), attendance may be made practically more difficult (O) by a lack of money for transport (M) or to meet other competing demands (M). |
| CMOC 28: When patients have reduced or impaired mobility/functional abilities (C), they may feel unable to attend (O) because travel and attendance become physically/psychologically unmanageable (M) or unsafe (M). |
| CMOC 6: Where patients experience a high degree of fear or anxiety around aspects of attending (C), non-attendance may become a protective strategy (O) because the appointment represents a source of threat and non-attendance a route to safety (M). |
| CMOC 14: Where people with access to fewer resources are exposed to a greater number of urgent, competing, or conflicting demands (C) they may be unable to attend (O), because prioritising resources to attend to those demands means they are not available to attend healthcare appointments (M). |
| CMOC 15: Where patients have several health conditions or complex needs (C) and fewer flexible resources (C) they may be less likely to attend appointments (O) because “treatment burden” exhausts or overwhelms their resources (M). |
| CMOC 18: When patients are isolated/lack social support (C), they may be less likely to attend (O) because they do not have people to help with practicalities of attendance (M), to support with managing competing demands (M), to overcome fears and anxieties around attending (M) or to facilitate communication (M). |
| CMOC 19: When patients are reliant on other people to help them attend (C), their non-attendance (O) may be connected to the other person’s availability, and the influence of other demands on them (M); the other person’s perception of the value of the appointment (M); the other person’s resources (M). |

# CMOCs and illustrative data

| **CMOC Number and description** | **Illustrative data excerpt** |
| --- | --- |
| CMOC 1: Where patients do not perceive their health/health conditions to be serious (C), they may be less inclined to attend (O) because they do not see appointments as beneficial or necessary (M) or worth the expenditure of resources (M) | “Responses by cases to “If you have not seen anyone about your diabetes recently, why is this?” [included] a belief that the diabetes had gone (five (14%)), that they felt all right, did not need to attend, that their diabetes was not a serious problem.” (81, p.111) |
| CMOC 2: Where patients have past experiences of being unable to improve health (for example, because of enduring poor health, complex health/non-health needs, or past experiences of services being unable to help) (C) they may not attend (O) because they feel that improvements are not desirable, possible or within their/service control (M) | “Among these factors, lower appointment expectancy (OR = 1.93, 95% CI =\1.00–3.71) and lower self-efﬁcacy (OR = 2.24, 95% CI = 1.24–4.05) predicted greater risk for non-adherence.”(67, p.553) |
| CMOC 3: Where attendance involves acting counter to normative expectations (C), non-attendance may occur (O) because attendance may place someone at risk of exclusion/sanction/stigma within families, peer groups and communities (M). | “The top reason that participants gave for ever missing their appointments at the HIV clinic was related to stigma. […] One fifth of our participants had not told anyone (apart from healthcare professionals) about their HIV status, and about two thirds reported markers of internalised HIV stigma. […] One fifth of those with sub-optimal attendance in the past year had missed appointments because they did not want to be seen at the clinic.”(28, p.3626) |
| CMOC 4: Where services do not communicate about patients’ health in ways that are accessible, meaningful and resonant (C), patients may not attend (O) because they do not perceive benefits (M) and may feel the service is not ‘for them.’ (M) | “While perceptions may be complex and influenced by previous experiences and beliefs, doctor–patient communication was influential. Some patients were unaware of the potential appointment urgency and reported that their GP did not explain:  *'[The GP] didn’t sort of explain that much. I didn’t really know what it was all about.’* (Pt111, F, age 22 years).”(91, p.e854)  “He’d asked the doctor … Just to let me know what it’s all about, you know what I mean? Half the time, I don’t understand what they’re saying. I don’t know what they’re saying, or what they’re talking about, what’s wrong with me.”(87, p.18) |
| CMOC 5: Where patients have difficult histories around caring relationships (psychological trauma, attachment issues, ‘relational injuries’) (C), they may not be willing to attend (O) because asking for help is inhibited by mistrust (M), feels unsafe/threatening or antithetical to their core relational beliefs (M) or they may be concerned that they will struggle to manage interpersonal elements of appointments (M). | “[Adelaide’s] explanations of failure to engage in therapy were somewhat interlinked within her narrative on insecure attachments in early childhood and her inability to trust others or to make positive relationships. In a similar way she didn’t believe professionals were trustworthy and didn’t expect to be cared for. […] The notion of abandonment hinted at within Adelaide’s story emerged throughout other patients’ narratives.”(44, p.431) |
| CMOC 6: Where patients experience a high degree of fear or anxiety around aspects of attending (C), non-attendance may become a protective strategy (O) because the appointment represents a source of threat and non-attendance a route to safety (M) | “Participants were hesitant when they were certain they would endure some uncomfortable procedures, […] Ms. U remembered her feelings regarding an appointment she had missed: “I said, ‘Nope, I’m not going! That’s uncomfortable’… so I just didn’t come.” While Ms. U was willing to make the appointment for follow-up care as her doctor had advised, she chose not to keep the appointment because of her negative anticipation of the visit. […] Fear was another barrier. Ms. H’s response typiﬁed fear of the unknown: “I just don’t want to go to the doctor. I’m scared they might tell you something, some bad news…. Come in with a headache and they say you’ve got a big brain tumor.”(90, p.543) |
| CMOC 7: Where there is misalignment between patients’ wishes, needs and circumstances (candidacies), and what is offered by services (C), non-attendance may occur (O) because patients do not trust the service (M) or feel what the service offers is ‘for them’ (M) | “Most participants described, at length, issues of perceived misdiagnosis or what they viewed as careless treatment plans that undermine their trust in providers. One woman described how she would cancel appointments or not show up for appointments because she did not want to hear that her physical health problem was instead mental or behavioural. Others described scenarios in which they had received a misdiagnosis that led to unnecessary surgeries, medications, and interventions.”(42, pp.4-5) |
| CMOC 8: When patients anticipate negative interactions with services (hostility, judgement, neglect, disrespect, stigma) (C), attendance is made difficult (O) because the appointment represents a source of threat and non-attendance a route to safety (M) | “Participants described being repeatedly proﬁled in care settings, with dismissive and judgemental attitudes contributing to hesitation to ﬁnd better care or change providers, as well as ‘reactive avoidance’ (Muncan et al., 2020) in anticipation of harm – leading many to forgo medically necessary or timely care. […] Discriminatory experiences in the healthcare system were highly gendered, with women, particularly transgender women, experiencing disproportionate structural violence within the healthcare system, and pervasive stigma related to both current or former substance use as well as their status as DTES residents. An overarching sentiment among the women’s group was that care experiences often lacked recognition of the humanity of those accessing care, and that providers showed a fundamental and consistent lack of respect towards them.”(129, pp769-772) |
| CMOC 9: Service responses to non-attendance that are punitive, sermonising or stigmatising (C) make the service space unsafe, unwelcoming and unhelpful (M) leading to patients not attending (O) | “Relationships, appointment systems and attitudes to non-attendance were clearly important as individual issues and there may also be a ‘feedback effect’ acting between them. Some patients fail to attend because of negative experiences in booking appointments or a poor relationship with the GP or practice in general and this in turn may result in a hardening of attitudes and a further deterioration in relationships.”(140, p.641) |
| CMOC 10: When patients have a background of experienced, internalised and anticipated stigma or discrimination around their identities, lifestyles or health (C) they may be less willing to attend (O) because: they do not feel worthy or deserving of help and support (M); may want to avoid thinking about their stigmatised health condition (M); may anticipate further stigma at the service (M) and thus appointments may be perceived as unthreatening/unsafe (M). | “[…] scared of a positive diagnosis because they believed the infection was not curable, that it resulted in premature death and they would feel ashamed if they were infected because of perceived stigma.”(73, p.134)  “[…] Others perceived themselves as being victims of discrimination and stigmatisation by HCPs due to their living circumstances, immigration status, and health issues. Some participants recounted mistrust between themselves and the HCPs […] The influence of staff attitude on a patient’s health-seeking behaviour was emphasised by several participants ad said to be ‘a decider between life and death’ for some patients who are homeless.”(74, p.e532) |
| CMOC 11: When patients have communication needs which are not addressed at each stage of candidacy (C), they may be less likely to attend (O) because they may feel unheard and may not be able to give/receive information that will make the appointment instrumentally beneficial (M) or they may feel a sense of discrimination, exclusion, neglect or stigma (M). | “Understanding what is being explained, the health issue and associated treatment can be challenging for people experiencing homelessness. Some have poor literacy, others have limited English. This can result in missed appointments and a lack of adherence to treatment regimes. It also creates a lack of confidence in, and ability to, engage with and navigate the healthcare system, and builds barriers to future interaction with health services.”(87, p.10) |
| CMOC 12: Where there are unaddressed power disparities in interactions with services (C) patients may mistrust providers (O) because they feel excluded (M), that there is little space for their own involvement in their own care (M), or that services are neglectful, intrusive or coercive (M) | “Distrust was reinforced when participants felt that they were treated as inferior and talked down to by providers, who they believed neglected their lived experiences and knowledges about their own health needs; as one participant recounted: ‘I think they talk down to me, they don’t give credit that you’re actually intelligent people’ (participant, FGD1). According to participants, judgement and disregard, which comes from not being listened to, affected the relationship for both sides, making it difﬁcult to get things done.”(129, p.770) |
| CMOC 13: Where services do not take account of patients’ wider circumstances or view patients as people (C), patients may not trust services (O) because they feel labelled or stereotyped (M), may come to believe that what is offered does not fit with their needs or best interests (M). | “where services have taken a shortcut and made assumptions about needs based on generic labels this was picked up by people and experienced as 'silencing' and 'uncaring'.”(112, p.25)  Negative stereotyping and labelling of a patient, such as a “no show,” and the disrespect for personal circumstances, are important dimensions of invalidating the experiences of marginalized clients.”(69, p.90) |
| CMOC 14: Where people with access to fewer resources are exposed to a greater number of urgent, competing, or conflicting demands (C) they may be unable to attend (O), because prioritising resources to attend to those demands means they are not available to attend healthcare appointments (M). | “…our interviews with [frequent non-attenders] provided insight not only into why patients found it difficult to keep appointments but also into their lives, giving us a sense of the struggles they face in managing the complexity of family, money, and emotional and physical health.”(113, p.639) |
| CMOC 15: Where patients have several health conditions or complex needs (C) and fewer flexible resources (C) they may be less likely to attend appointments (O) because “treatment burden” exhausts or overwhelms their resources: | “There are times when I just get so fed up with all the pills and all the doctor’s appointments. I’m guilty. I just walk away. […] I won’t go to doctor’s appointments and I stop taking my medications just because I gotta take a break. I got to. Because I would like just for a little bit to feel like a normal person. […] There are times when I just have to—have to take a break. I just gotta. Then I disengage.” [...] less-well-retained patients experienced HIV care as burdensome to a higher degree than retained participants [...] This depiction of care engagement as burdensome by less-well-retained patients makes sense in light of the presence of vulnerability among patients with fewer material and emotional resources to draw on than retained patients.”(41, p.8) |
| CMOC 16: Patients who work in low-paid, inflexible, casual, or hourly-paid employment (C) or who have multiple appointments (C) may not be able to attend (O) because they cannot get time away from work (M), or because taking time off has costs that they cannot bear (M). | “I do have problems because the minute you say you have to do this [the employer] want, ‘Can't another member of the family?’... I got an appointment for my daughter, ‘Well, you should've booked it.’ Well most of the time I'll end up losing pay over it… It can be very frustrating, that sometimes I have to lie, sometimes I have to say I'm not well. Just to be able to take her to the hospital.’ […] More commonly, interviewees mentioned concerns about taking ‘too much’ time off work to attend appointments, stating they had “still got to work” (P22) or “couldn’t keep having time off work” (P12). This perceived restriction was more likely to be problematic when children had many or frequent appointments.”(54, p.103) |
| CMOC 17: Patients who have caring responsibilities (C) may struggle to attend scheduled appointments (O) because they prioritise using their resources to meet the needs/demands/urgencies of the people they care for (M). | “Having ‘*children’* was a reason for non-attendance due to competing demands. The participants explained that there might not be anyone else to look after them, they needed collecting from school or contact with their child arranged through social services was on weekdays and not at the weekends.” (73, p.134) |
| CMOC 18: When patients are isolated/lack social support (C), they may be less likely to attend (O) because they do not have people to help with practicalities of attendance (M), to support with managing competing demands (M), to overcome fears and anxieties around attending (M) or to facilitate communication (M) | “Some users had no family or significant others, whereas others could not or would not ask family or other network to accompany them; afraid of being a burden, wanting children to live their own lives without worrying about their parents, or finding relatives incapable of providing the help needed. […] Relying on others to make it to health appointments makes visible one’s network or lack of network.”(137, p.6) |
| CMOC 19: When patients are reliant on other people to help them attend (C), their non-attendance (O) may be connected to the other person’s availability, and the influence of other demands on them (M); the other person’s perception of the value of the appointment (M); the other person’s resources (M). | “Many participants emphasized that their reliance on others for transportation rendered appointment attendance out of their control. Sometimes others were not able or willing to transport them to appointments and the participants were left with few options. One participant discussed his temporary reliance on his sister for transportation and issues with attending appointments due to the instability of her work schedule. Another participant explained that she had made prior arrangements to have others take her to her appointment but the individuals were not available when the time came.”(42, p.6) |
| CMOC 20: Where practices ask patients to see practitioners that they do not choose or have no relationship with (C) patients may be: less willing to attend (O) because of their fears and concerns about seeing someone they do not know or trust (M); because the practitioner knows less about their lives/circumstances/wishes/needs and therefore their advice/support is perceived to be less beneficial (M) or communicating with them is more difficult (M). | “Concerns were expressed that doctors who are constantly changing feel less responsible towards individual patients and, furthermore, often do not have an opportunity to get to the bottom of things: ‘‘… if you are visiting a new doctor, they don’t have the responsibility of the patient as their, their own responsibility to manage that patient. […] You can’t discuss your physical situation …You don’t come to the fine details basically, you know, cannot go from, one point to the other. And what happens is that you start and give him the full details and now the conversation cannot go into the next gear, the next level of detail.”(34, p.193) |
| CMOC 21: When practices or services offer less control, choice or flexibility over the day and time of an appointment to patients (C), they are more likely to miss it (O) because it may conflict with other urgent demands (M). | “Reduced patient involvement is identified as another factor associated with DNAs, and several of the review articles shared key features of improving communication and encouragement of patients in making their own appointments, both of which were found to be major determinants of non-attendance. In doing this, underlying causes of non-attendance, such as poor communication, short notification and inconvenient timings were negated” (26, p.905) |
| CMOC 22: Where there are delays between a person making an appointment and the appointment itself (C), patients may be less likely to attend (O) because: their symptoms might improve and they feel the appointment is no longer necessary (M); they may worsen and attendance becomes practically impossible (M); they may be more likely to forget; other competing demands may emerge or take priority (M); fears or anxieties may increase; patients may feel disrespected, neglected or unwanted (M). | “The review generated strong consistent evidence that a longer time between the date of the appointment being made and the date of the appointment taking place is associated with higher non-attendance. […] it has been suggested that other issues are more likely to arise that mean the appointment timing becomes incompatible. It has also been suggested that when appointments are scheduled a long time in advance, patient symptoms may be resolved, either because they resolve themselves or because the patient (or referring health-care professional) opts to seek alternative care […] some authors suggested that under particular circumstances, a long delay could result in reduced motivation on the part of the patient despite continued need […] It may be that scheduling an appointment a long time in advance can convey the impression to the patient that the appointment is unimportant.”(13, p.57) |
| CMOC 23: Where patients are offered appointments on very short notice (C), attendance may be prevented (O) because they cannot negotiate their competing demands (M) or access the resources they need to facilitate attendance (M). | “50% of the non-attenders in this study had been given notice of a week or less, which is considered inadequate when transport, family and work commitments are to be re-arranged.”(33, p.285) |
| CMOC 24: When there are administrative errors with appointment scheduling and/or communication (C) patients do not attend (M) because they are given the wrong details for their appointments, or never receive those details and so are not aware of when they are expected (M). | “The most common reason given for non-attendance was not receiving the appointment. The next most prevalent reason was forgetting about the appointment. […] The category of “administrative problems” accounted for 80% (106) of all non-attenders, of which two-thirds (67) were at an institutional level.”(65, p.66) |
| CMOC 25: If services do not have systems for patients to contact them to cancel or rearrange, or these systems are burdensome (C), patients may miss appointments (O) because they are unable to cancel or amend appointments. | “Within the category 'misunderstandings and mistakes', the largest sub-category was 'by the practice'. Further, in the response to the pre-defined categories, 30% stated that they had tried to cancel their appointment.”(19, p.4)  “The miscommunication category included patients who said they thought they had canceled the appointment, patients who thought the appointment was a different date or time, patients who tried to call the clinic but did not get through, and patients who did not realize they needed to call and cancel.”(20, p.253) |
| CMOC 26: When people have illnesses or conditions that affect cognition or memory (C), they may forget to attend appointments (O), struggle with logistics (O) or feel unsafe or anxious in managing the demands of appointment attendance (O) because of their cognitive difficulties (M). | “Tom also had developed an opiate dependency, alcohol dependency, severe anxiety, memory lapses and sometimes poor cognition and confusion. […] His memory difficulties and poor physical health can leave him struggling to remember appointments, which can result in missed attendances. When Tom tries to book appointments again, he is met with resistance, reminded of his past missed attendances with no constructive solution for the problem.”(93, p.22) |
| CMOC 27: When patients live in poverty (C), attendance may be made practically more difficult (O) by a lack of money for transport (M) or to meet other competing demands (M). | “I know I have to look after my diabetes, but they stop our benefits 2 month ago. When I have to go to appointments, I take three buses or pay £5 to go and £5 to come back by taxi, so you tell me how easy it is to get to my appointments.”(114, p.76)  “In December, 11% of respondents said they had avoided attending an NHS appointment because they couldn’t afford to travel to it, up from 6% in October.”(183, p.4) |
| CMOC 28: When patients have reduced or impaired mobility/functional abilities (C), they may feel unable to attend (O) because travel and attendance become physically/psychologically unmanageable (M) or unsafe (M). | “A PTSD diagnosis causes fear and anxiety and some patients had severe panic attacks and flashbacks, which gave them difficulties in using public transport:  *“I have had a patient who had some incidents where she almost fainted and fell over, so she was so afraid to take the bus and she lived relatively far away. Even though she was a young girl and did not have any physical problems, she feared that she would suddenly become ill or dizzy.””*(135, p.319) |
| CMOC 29: Where physical spaces are not welcoming or comfortable (C), patients may not feel comfortable attending (O) because they do not feel safe, secure or welcome (M). | “Several clients stated that poor therapeutic environment made significant impact on their decisions to attend their sessions in the past. For clients, spacious, welcoming, clean, private, discrete, and multipurpose environment contributed to positive therapeutic environment.”(72, p.232) |
| CMOC 30: When different aspects of patients’ candidacies (identifications, navigations, presentations, resistances) have been challenged, undermined or questioned (C), they have low confidence in their capacity to successfully navigate services (O) or develop mistrust of the service (O), because of the repeated rejection (M) | “Distrust was reinforced when participants felt that they were treated as inferior and talked down to by providers, who they believed neglected their lived experiences and knowledges about their own health needs; as one participant recounted: ‘I think they talk down to me, they don’t give credit that you’re actually intelligent people’ (participant, FGD1). According to participants, judgement and disregard, which comes from not being listened to, affected the relationship for both sides, making it difﬁcult to get things done.”(129, p.770) |

# Evidence synthesis bibliography

1. Lindsay C, Baruffati D, Mackenzie M, Ellis DA, Major M, O'Donnell K, et al. A realist review of the causes of, and current interventions to address ‘missingness’ in health care. *NIHR Open Res*. 2023;3.

2. Dixon-Woods M, Cavers D, Agarwal S, Annandale E, Arthur A, Harvey J, et al. Conducting a critical interpretive synthesis of the literature on access to healthcare by vulnerable groups. *BMC Med* Res Methodol. 2006;6:1-13.

3. Mackenzie M, Gannon M, Stanley N, Cosgrove K, Feder G. ‘You certainly don't go back to the doctor once you've been told,“I'll never understand women like you.”’Seeking candidacy and structural competency in the dynamics of domestic abuse disclosure. *Sociol Health Ill*. 2019;41(6):1159-74.

4. Quesada J, Hart LK, Bourgois P. Structural vulnerability and health: Latino migrant laborers in the United States. *Med Anthropol*. 2011;30(4):339-62.

5. Freese J, Lutfey K. Fundamental causality: challenges of an animating concept for medical sociology. In *Handbook of the sociology of health, illness, and healing: A blueprint for the 21st century*: Springer; 2010. p. 67-81.

6. Wong G, Brennan N, Mattick K, Pearson M, Briscoe S, Papoutsi C. Interventions to improve antimicrobial prescribing of doctors in training: the IMPACT (IMProving Antimicrobial presCribing of doctors in Training) realist review. *BMJ Open*. 2015;5(10):e009059.

7. Clouston SA, Link BG. A retrospective on fundamental cause theory: State of the literature and goals for the future. *Annu Rev Sociol*. 2021;47:131-56.

8. Lutfey K, Freese J. Toward some fundamentals of fundamental causality: Socioeconomic status and health in the routine clinic visit for diabetes. *Am J sociol*. 2005;110(5):1326-72.

9. Bourdieu P. *The logic of practice:* Stanford university press; 1990.

10. Veenstra G. Infusing fundamental cause theory with features of Pierre Bourdieu’s theory of symbolic power. *Scand J Public Health*. 2018;46(1):49-52.

11. Baruffati D. A Bourdieusian ethnographic exploration of the fundamental causation of health among men across two Glasgow neighbourhoods: Thesis, University of Glasgow; 2022.

12. Mackenzie M, Conway E, Hastings A, Munro M, O'Donnell C. Is ‘candidacy’a useful concept for understanding journeys through public services? A critical interpretive literature synthesis. *Soc Policy Adm*. 2013;47(7):806-25.

13. McLean S, Gee M, Booth A, Salway S, Nancarrow S, Cobb M, et al. Targeting the use of reminders and notifications for uptake by populations (TURNUP): a systematic review and evidence synthesis. *Health Services and Delivery Research.* 2014;2(34).

14. Dixon-Woods M, Kirk MD, Agarwal MS, Annandale E, Arthur T, Harvey J, et al. *Vulnerable groups and access to health care: a critical interpretive review.* National coordinating centre NHS service delivery organ RD (NCCSDO). 2005. https://citeseerx.ist.psu.edu/document?repid=rep1&type=pdf&doi=c31d40b30bf0fce7dd0e82bf9dde437db5285b9b [accessed 30.04.2024]

15. Haynes A, Brennan S, Redman S, Williamson A, Makkar SR, Gallego G, et al. Policymakers’ experience of a capacity-building intervention designed to increase their use of research: a realist process evaluation. *Health Res Policy Syst*. 2017;15(1):99.

16. Greenhalgh T WG, Westhorp G. *Retroduction in realist evaluation.* The RAMESES II Project, 2017. https://www.ramesesproject.org/media/RAMESES_II_Retroduction.pdf [accessed 30.04.2024]

17. Martin SJ, Bassi S, Dunbar-Rees R. Commitments, norms and custard creams - a social influence approach to reducing did not attends (DNAs). *J R Soc Med*. 2012;105(3):101-4.

18. Hamilton W. Non-attendance in general practice: a questionnaire. *Prim Health Care Res Dev*. 2002;3(4):226-30.

19. Neal RD, Hussain-Gambles M, Allgar VL, Lawlor DA, Dempsey O. Reasons for and consequences of missed appointments in general practice in the UK: questionnaire survey and prospective review of medical records. *BMC* *Fam Pract*. 2005;6:47.

20. Kaplan-Lewis E, Percac-Lima S. No-Show to Primary Care Appointments: Why Patients Do Not Come. *J Prim Care Community Health*. 2013;4(4):251-5.

21. Shahab I, Meili R. Examining non-attendance of doctor's appointments at a community clinic in Saskatoon. *Can Fam Physician*. 2019;65(6):E264-E8.

22. Zailinawati AH, Ng CJ, Nik-Sherina H. Why do patients with chronic illnesses fail to keep their appointments? A telephone interview. *Asia-Pac J Public Health*. 2006;18(1):10-5.

23. Ullah S, Rajan S, Liu T, Demagistris E, Jahrstorfer R, Anandan S, et al. Why do patients miss their appointments at primary care clinics. *J Fam Med Dis Prev.* 2018;4(3):1-5.

24. Deyo RA, Inui TS. Dropouts and broken appointments. A literature review and agenda for future research. *Med Care*. 1980;18(11):1146-57.

25. Parsons J, Bryce C, Atherton H. Which patients miss appointments with general practice and the reasons why: a systematic review. *Br J Gen Pract*. 2021;71(707):E406-E12.

26. Wilson R, Winnard Y. Causes, impacts and possible mitigation of non-attendance of appointments within the National Health Service: a literature review. *J Health Organ Manag*. 2022;36(7):892-911.

27. Dyer BT, Swann F, Kadam M, Draper J, Mc Gill LA, Kapetanakis S, et al. Understanding non-attendance to an inner city tertiary centre heart failure clinic: A pilot project. *Eur Heart J.* 2019;40(Supplement 1):3747.

28. Howarth AR, Apea V, Michie S, Morris S, Sachikonye M, Mercer CH, et al. Associations with sub-optimal clinic attendance and reasons for missed appointments among heterosexual women and men living with HIV in London. *AIDS Behav*. 2022;26(11):3620-9.

29. Lakshminarayana I. Measures to improve non attendance rates of community paediatric outpatient clinics. *Arch Dis Child*. 2016;101(Supplement 1):A106.

30. Magan T, Kirmani A, Robertson M, Mohamed M, Mann S. Non-attendance in the ranibizumab treatment clinic for diabetic macular oedema: Rates and reasons. *Eur J Ophthalmol*. 2014;24(3):465-6.

31. Mault S, McDonough BJ, Currie P, Burhan H. Reasons proffered for non-attendance at a difficult asthma clinic. *Thorax*. 2012;67(SUPPL. 2):A187.

32. Pal B, Taberner DA, Readman LP, Jones P. Why do outpatients fail to keep their clinic appointments? Results from a survey and recommended remedial actions. *Int J Clin Pract.* 1998;52(6):436-7.

33. Lyon R, *Reeves* PJ. An investigation into why patients do not attend for out-patient radiology appointments. Radiography. 2006;12(4):283-90.

34. van Baar JD, Joosten H, Car J, Freeman GK, Partridge MR, van Weel C, et al. Understanding reasons for asthma outpatient (non)-attendance and exploring the role of telephone and e-consulting in facilitating access to care: exploratory qualitative study. *Qual Saf Health Care*. 2006;15(3):191-5.

35. Unger K, Lesiuk A, Unger S. 947 How do we save£ 1200 lost to DNAs’ per complex paediatric respiratory clinic and protect our most vulnerable patients? *Arch Dis Child* 2023;108:A441.

36. Liu S, Ng JKY, Moon EH, Morgan D, Woodhouse N, Agrawal D, et al. Impact of COVID-19-associated anxiety on the adherence to intravitreal injection in patients with macular diseases a year after the initial outbreak. *Ther Adv Ophthalmol.* 2022:1-12.

37. Robotham D, Satkunanathan S, Reynolds J, Stahl D, Wykes T. Using digital notifications to improve attendance in clinic: systematic review and meta-analysis. *BMJ Open*. 2016;6(10):14.

38. Henry SR, Goetz MB, Asch SM. The effect of automated telephone appointment reminders on HIV primary care no-shows by veterans. *J Assoc Nurses AIDS Care*. 2012;23(5):409-18.

39. Amberger C, Schreyer D. What do we know about no-show behavior? A systematic, interdisciplinary literature review. *J Econ Surv*. 2022;38:57–96

40. Gurewich D, Linsky AMM, Harvey KLL, Li M, Griesemer I, MacLaren RZZ, et al. Relationship Between Unmet Social Needs and Care Access in a Veteran Cohort. *J Gen Intern Med*. 2023;38(SUPPL 3):841-8.

41. Koester KA, Johnson MO, Wood T, Fredericksen R, Neilands TB, Sauceda J, et al. The influence of the 'good' patient ideal on engagement in HIV care. *PLoS One*. 2019;14(3).

42. Chapman KA, Machado SS, van der Merwe K, Bryson A, Smith D. Exploring Primary Care Non-Attendance: A Study of Low-Income Patients. *J Prim Care Community Health*. 2022;13.

43. Ciechanowski P, Russo J, Katon W, Simon G, Ludman E, Von Korff M, et al. Where is the patient? The association of psychosocial factors and missed primary care appointments in patients with diabetes. *Gen Hosp Psychiatry.* 2006;28(1):9-17.

44. Leavey G, Vallianatou C, Johnson-Sabine E, Rae S, Gunputh V. Psychosocial barriers to engagement with an eating disorder service: a qualitative analysis of failure to attend. *Eat Disord.* 2011;19(5):425-40.

45. Edwards K. *Identifying Patient Preferences in Appointment Reminders for Adults to Reduce Missed Appointments in an Outpatient Mental Health Clinic: A Quality Improvement Project.* Dissertation, Georgia Southern University. 2023. https://scholarworks.gsu.edu/nursing_dnpprojects/43/ [accessed 01.05.2024]

46. Bean AG, Talaga J. Appointment breaking: causes and solutions. *J Health Care Mark.* 1992;12(4):14-25.

47. Eades C, Alexander H. A mixed-methods exploration of non-attendance at diabetes appointments using peer researchers. *Health Expect*. 2019;22(6):1260-71.

48. Jones MC, Smith K, Herber O, White M, Steele F, Johnston DW. Intention, beliefs and mood assessed using electronic diaries predicts attendance at cardiac rehabilitation: an observational study. *Int J Nurs Stud*. 2018;88:143-52.

49. Bowser DM, Utz S, Glick D, Harmon R. A Systematic Review of the Relationship of Diabetes Mellitus, Depression, and Missed Appointments in a Low-Income Uninsured Population. *Arch Psychiatr Nurs*. 2010;24(5):317-29.

50. Roberts L, Garo-Falides J, Bowran H. Non-attendance in musculoskeletal outpatients: The good, the bad and the ugly*. Physiotherapy (United Kingdom).* 2015;101(SUPPL. 1):eS1289-eS90.

51. Barron WM. Failed appointments - who misses them, why are they missed, and what can be done. *Prim Care*. 1980;7(4):563-74.

52. George A, Rubin G. Non-attendance in general practice: a systematic review and its implications for access to primary health care. *Fam Pract*. 2003;20(2):178-84.

53. Brewster SZ. *The Role of Community Pharmacy in Supporting People with Diabetes Who Have a History of Repeated Non-Attendance at Healthcare Appointments. Dissertation, University of Southampton.* 2023. https://eprints.soton.ac.uk/475222/ [accessed 01.05.2024]

54. Cameron EZ. *A mixed methods investigation of parental factors in non-attendance at general paediatric hospital outpatient appointments.* Dissertation, Aston University. 2015. https://research.aston.ac.uk/en/studentTheses/a-mixed-methods-investigation-of-parental-factors-in-non-attendan [accessed 01.05.2024]

55. Brown S. *Qualitative evaluation of Focused Care.* Focused Care, 2019. https://focusedcare.org.uk/wp-content/uploads/2020/11/FC-Qualitative-Eval.pdf. [Accessed 29.04.2024]

56. Parkes T, Matheson C, Carver H, Foster R, Budd J, Liddell D, et al. A peer-delivered intervention to reduce harm and improve the well-being of homeless people with problem substance use: the SHARPS feasibility mixed-methods study. *Health Technol Assess*. 2022;26(14):1-128.

57. Crane MA, Cetrano G, Joly LMA, Coward S, Daly BJM, Ford C, et al. *Mapping of specialist primary health care services in England for people who are homeless. Summary of findings and considerations for health service commissioners and providers.* 2018. Social Care Workforce

Research Unit, King's College London. https://doi.org/10.18742/pub01-091 [accessed 29.04.2024]

58. Claveau J, Authier M, Rodrigues I, Crevier-Tousignant M. Patients' missed appointments in academic family practices in Quebec. *Can Fam Physician*. 2020;66(5):349-55.

59. Samuels RC, Ward VL, Melvin P, Macht-Greenberg M, Wenren LM, Yi J, et al. Missed Appointments: Factors Contributing to High No-Show Rates in an Urban Pediatrics Primary Care Clinic. *Clin Pediatr*. 2015;54(10):976-82.

60. Dockery F, Rajkumar C, Chapman C, Bulpitt C, Nicholl C. The effect of reminder calls in reducing non-attendance rates at care of the elderly clinics. *Postgrad Med J*. 2001;77(903):37-9.

61. Morris L, Haywood S. Why do patient miss appointments? A retrospective population study in paediatric outpatients in a metropolitan hospital. *Arch Dis Child*. 2014;99(SUPPL. 1):A96.

62. Wilsey KZ. *Why Patients Miss Appointments at an Integrated Primary Care Clinic.* Dissertation, Antioch University, 2020. https://www.proquest.com/docview/2461616923?pq-origsite=gscholar&fromopenview=true&sourcetype=Dissertations%20&%20Theses [accessed 30.04.2024]

63. Ayalde J, Soong W, Thomas S, McCann P, Griffiths J, Nicholls C, et al. Reasons for non-attendance in youth mental health clinics: Insights from mobile messaging communications. *Early Interv Psychiatry.* 2023;17(9):877-83.

64. Cosgrove MP. Defaulters in general practice: reasons for default and patterns of attendance. *Br J Gen Pract*. 1990;40(331):50-2.

65. Hull AM, Alexander DA, Morrison F, McKinnon JS. A waste of time: non-attendance at out-patient clinics in a Scottish NHS Trust. *Health Bull (Edinb)*. 2002;60(1):62-9.

66. Mason C. Non-attendance at out-patient clinics: a case study. J Adv Nurs. 1992;17(5):554-60.

67. Traeger L, O'Cleirigh C, Skeer MR, Mayer KH, Safren SA. Risk factors for missed HIV primary care visits among men who have sex with men. *J Behav Med*. 2012;35(5):548-56.

68. Buetow S. Non-attendance for health care: When rational beliefs collide*. Sociol Rev.* 2007;55(3):592-610.

69. Arnold OF. Reconsidering the "NO SHOW" Stamp: Increasing Cultural Safety by Making Peace with a Colonial Legacy. *Northern Review*. 2012(36):77-96.

70. Herber OR, Smith K, White M, Jones MC. ‘Just not for me’–contributing factors to nonattendance/noncompletion at phase III cardiac rehabilitation in acute coronary syndrome patients: a qualitative enquiry. *J Clin Nurs.* 2017;26(21-22):3529-42.

71. Abraham CS, Paschal. The Health Belief Model. In: Conner MN, Paul, editor. *Predicting and Changing Health Behaviour: Research and Practice with Social Cognition Models*. 3 ed. UK: McGraw-Hill; 2015.

72. Mahmood FZ. *Exploring Reasons for Clients' Non-Attendance at Appointments Within a Community-Based Alcohol Service : Clients' and Practitioners' Perspectives*. Dissertation, Manchester Metropolitan University, 2021. https://e-space.mmu.ac.uk/628034/1/FaisalMahmood_14501562_PhDThesis_Final.pdf [accessed 30.04.2024]

73. Poll R, Allmark P, Tod AM. Reasons for missed appointments with a hepatitis C outreach clinic: A qualitative study*. Int J Drug Policy*. 2017;39:130-7.

74. Gunner E, Chandan SK, Marwick S, Saunders K, Burwood S, Yahyouche A, et al. Provision and accessibility of primary healthcare services for people who are homeless: a qualitative study of patient perspectives in the UK. *Br J Gen Pract*. 2019;69(685):e526-e36.

75. Moscrop A, Siskind D, Stevens R. Mental health of young adult patients who do not attend appointments in primary care: a retrospective cohort study. *Fam Pract*. 2012;29(1):24-9.

76. Winkley K, Evwierhoma C, Amiel SA, Lempp HK, Ismail K, Forbes A. Patient explanations for non-attendance at structured diabetes education sessions for newly diagnosed Type 2 diabetes: a qualitative study. *Diabet Med*. 2015;32(1):120-8.

77. Thapar A, Ghosh A. Non-attendance at a psychiatric clinic. *Psychiat Bull.* 1991;15(4):205-6.

78. Hamilton W. General practice non-attendance [11]. *Br J Gen Pract*. 1999;49(445):664.

79. Cameron E, Heath G, Redwood S, Greenfield S, Cummins C, Kelly D, et al. Health care professionals' views of paediatric outpatient non-attendance: implications for general practice. *Fam Pract*. 2014;31(1):111-7.

80. Corfield L, Schizas A, Williams A, Noorani A. Non-attendance at the colorectal clinic: a prospective audit. *Ann R Coll Surg Engl*. 2008;90(5):377-80.

81. Simmons D, Clover G. A case control study of diabetic patients who default from primary care in urban New Zealand. *Diabetes Metab*. 2007;33(2):109-13.

82. Minshall I, Neligan A. A review of people who did not attend an epilepsy clinic and their clinical outcomes. *Seizure*. 2017;50:121-4.

83. McQueenie R, Ellis DA, McConnachie A, Wilson P, Williamson AE. Morbidity, mortality and missed appointments in healthcare: a national retrospective data linkage study. *BMC Med*. 2019;17:9.

84. Gonzalez JS, Peyrot M, McCarl LA, Collins EM, Serpa L, Mimiaga MJ, et al. Depression and Diabetes Treatment Nonadherence: A Meta-Analysis. *Diabetes Care*. 2008;31(12):2398-403.

85. Inglesfield J. Non-attendance and mental health problems in primary care [11]. *Br J Gen Pract*. 1999;49(443):488-9.

86. Horigan G, Davies M, Findlay‐White F, Chaney D, Coates V. Reasons why patients referred to diabetes education programmes choose not to attend: a systematic review. *Diabet Med*. 2017;34(1):14-26.

87. Finlayson S, Boelman V, Young R, Kwan A. *SAVING LIVES, SAVING MONEY: How Homeless Health Peer Advocacy Reduces Health Inequalities*. Groundswell; 2015. https://groundswell.org.uk/wp-content/uploads/2018/10/Groundswell-Saving-Lives-Saving-Money-Full-Report-Web-2016.pdf [accessed 30.04.2024]

88. Sharp L, Cotton S, Thornton A, Gray N, Cruickshank M, Whynes D, et al. Who defaults from colposcopy? A multi-centre, population-based, prospective cohort study of predictors of non-attendance for follow-up among women with low-grade abnormal cervical cytology. *Eur J Obstet Gynecol Reprod Biol*. 2012;165(2):318-25.

89. Blankenstein R. Failed appointments - Do telephone reminders always work? *Clin Gov.* 2003;8(3):208-12.

90. Lacy NL, Paulman A, Reuter MD, Lovejoy B. Why we don't come: Patient perceptions on no-shows. *Ann Fam Med.* 2004;2(6):541-5.

91. Jefferson L, Atkin K, Sheridan R, Oliver S, Macleod U, Hall G, et al. Non-attendance at urgent referral appointments for suspected cancer: a qualitative study to gain understanding from patients and GPs. *Br J Gen Pract*. 2019;69(689):E850-E9.

92. Cashman SB, Savageau JA, Lemay CA, Ferguson W. Patient health status and appointment keeping in an urban community health center. *J Health Care Poor Underserved.* 2004;15(3):474-88.

93. Wilson B, Astley P. Gatekeepers: Access to Primary Care for those with Multiple Needs. 2016.

94. Hatzenbuehler ML, Phelan JC, Link BG. Stigma as a fundamental cause of population health inequalities. *Am J Public Health.* 2013;103(5):813-21.

95. Akter S, Doran F, Avila C, Nancarrow S. A qualitative study of staff perspectives of patient non-attendance in a regional primary healthcare setting. *Australas Med J.* 2014;7(5):218-26.

96. Aspinall PJ. *Inclusive practice: vulnerable migrants, gypsies and travellers, people who are homeless, and sex workers: a review and synthesis of interventions/service models that improve access to primary care & reduce risk of avoidable admission to hospital.* University of Kent; 2014. https://assets.publishing.service.gov.uk/media/5a7db8b2e5274a5eaea65ee4/Inclusive_Practice.pdf [accessed 30.04.2024]

97. Ellis DA, McQueenie R, McConnachie A, Wilson P, Williamson AE. Demographic and practice factors predicting repeated non-attendance in primary care: a national retrospective cohort analysis. *Lancet Public Health.* 2017;2(12):E551-E9.

98. Williamson AE, Ellis DA, Wilson P, McQueenie R, McConnachie A. Understanding repeated non-attendance in health services: a pilot analysis of administrative data and full study protocol for a national retrospective cohort. *BMJ Open*. 2017;7(2):11.

99. Williamson AE, McQueenie R, Ellis DA, McConnachie A, Wilson P. 'Missingness' in health care: Associations between hospital utilization and missed appointments in general practice. A retrospective cohort study. *PLoS One*. 2021;16(6):e0253163.

100. Barker I, Steventon A, Williamson R, Deeny SR. Self-management capability in patients with long-term conditions is associated with reduced healthcare utilisation across a whole health economy: cross-sectional analysis of electronic health records. *BMJ Qual Saf.* 2018;27(12):989-99.

101. Moscrop A. Would it be a good idea to charge for missed appointments at the doctors surgery? *BMJ Opinion.* 2015;351:23-.

102. Martin PM. Coroner inquest into 'hospital non-attendance' management in primary care. *Br J Gen Pract*. 2019;69(681):195.

103. Dantas LF, Fleck JL, Cyrino Oliveira FL, Hamacher S. No-shows in appointment scheduling - a systematic literature review. *Health Policy.* 2018;122(4):412-21.

104. Hussain-Gambles M, Neal RD, Dempsey O, Lawlor DA, Hodgson J. Missed appointments in primary care: questionnaire and focus group study of health professionals. *Br J Gen Pract*. 2004;54(499):108-13.

105. Corrigan PW, Pickett S, Schmidt A, Stellon E, Hantke E, Kraus D, et al. Peer navigators to promote engagement of homeless African Americans with serious mental illness in primary care. *Psychiatry Res*. 2017;255:101-3.

106. Margham T. Reducing missed appointments in general practice: evaluation of a quality improvement programme in East London (vol 71, pg e31, 2021). *Br J Gen Pract*. 2021;71(704):109-.

107. Waller J, Hodgkin P. Defaulters in general practice: who are they and what can be done about them? *Fam Pract*. 2000;17(3):252-3.

108. Herber OR, Jones MC, Smith K, Johnston DW. Assessing acute coronary syndrome patients' cardiac-related beliefs, motivation and mood over time to predict non-attendance at cardiac rehabilitation. *J Adv Nurs.* 2012;68(12):2778-88.

109. Fiori KP, Heller CG, Rehm CD, Parsons A, Flattau A, Braganza S, et al. Unmet Social Needs and No-Show Visits in Primary Care in a US Northeastern Urban Health System, 2018-2019. *Am J Public Health*. 2020;110:S242-S50.

110. Campbell K, Millard A, McCartney G, McCullough S. *Who is least likely to attend? An analysis of outpatient appointment ‘did not attend’(DNA) data in Scotland.* Edinburgh: NHS Health Scotland. 2015. https://www.healthscotland.scot/media/1129/5348_dna-analysis_nhs-ggc.pdf [accessed 30.04.2024]

111. Bansal I, Soni R, Eisen S, Ward A, Longley N, Sen C. 464 Breaking the barriers to accessing care: co-creating solutions with refugee service users. *Arch Dis Child*. 2023;108.

112. McCarthy L, Parr S, Green S, Reeve K. *Understanding models of support for people facing multiple disadvantage: A Literature Review.* 2020. Fulfilling Lives Lambeth, Southwark & Lewisham. https://www.shu.ac.uk/centre-regional-economic-social-research/publications/understanding-models-of-support-for-people-facing-multiple-disadvantage-a-literature-review [accessed 29.04.2024]

113. DuMontier C, Rindfleisch K, Pruszynski J, Frey JJ. A Multi-Method Intervention to Reduce No-Shows in an Urban Residency Clinic. *Fam Med*. 2013;45(9):634-41..

114. Campbell-Richards D. Exploring diabetes non-attendance: an Inner London perspective. *J Diabetes Nurs*. 2016;20(2):73-8.

115. Coulter A, Roberts S, Dixon A. *Delivering better services for people with long-term conditions: Building the house of care.* The King's Fund, 2013. https://www.kingsfund.org.uk/insight-and-analysis/reports/better-services-people-long-term-conditions [accessed 30.04.2024]

116. Prudden G. Quality improvement project exploring the factors in non-attendance at an NHS musculoskeletal outpatients department. *Physiotherapy (United Kingdom).* 2021;113(Supplement 1):e151-e2.

117. Bickler CB. Defaulted appointments in general practice. *J R Coll Gen Pract*. 1985;35(270):19-22.

118. Stevenson JS. Appointment systems in general practice: How patients use them. *BMJ*. 1967;2(5555):827-+.

119. Biggs J, Njoku N, Kurtz K, Omar A. Decreasing Missed Appointments at a Community Health Center: A Community Collaborative Project. *J Prim Care Community Health*. 2022;13:3.

120. Anyaegbu CT. SMS reminders: reducing DNA at a community mental health depot clinic. *Journal of Community Nursing.* 2021;35(1).

121. Boos EM, Bittner MJ, Kramer MR. A Profile of Patients Who Fail to Keep Appointments in a Veterans Affairs Primary Care Clinic. *WMJ*. 2016;115(4):185-90.

122. Lasser KE, Mintzer IL, Lambert A, Cabral H, Bor DH. Missed appointment rates in primary care: The importance of site of care. *J Health Care Poor Underserved*. 2005;16(3):475-86.

123. Nancarrow S, Bradbury J, Avila C. Factors associated with non-attendance in a general practice super clinic population in regional Australia: A retrospective cohort study. *Australas Med J.* 2014;7(8):323-33.

124. Parker MM, Moffet HH, Schillinger D, Adler N, Fernandez A, Ciechanowski P, et al. Ethnic Differences in Appointment-Keeping and Implications for the Patient-Centered Medical Home: Findings from the Diabetes Study of Northern California (DISTANCE). *Health Serv Res.* 2012;47(2):572-93.

125. Shimotsu S, Roehrl A, McCarty M, Vickery K, Guzman-Corrales L, Linzer M, et al. Increased Likelihood of Missed Appointments ("No Shows") for Racial/Ethnic Minorities in a Safety Net Health System. *J Prim Care Community Health*. 2016;7(1):38-40.

126. Dunmore C, Baldwin L, Akpan A. Social determinants and older people hospital outpatient non-attendance. *Age Ageing*. 2017;46(Supplement 3):iii1.

127. Mitchell AJ, Selmes T. A comparative survey of missed initial and follow-up appointments to psychiatric specialties in the United kingdom. *Psychiatric services (Washington, DC)*. 2007;58(6):868-71.

128. Neal RD, Lawlor DA, Allgar V, Colledge M, Ali S, Hassey A, et al. Missed appointments in general practice: retrospective data analysis from four practices. *Br J Gen Pract*. 2001;51(471):830-2.

129. Pakhomova TEE, Nicholson V, Fischer M, Ferguson J, Moore DMM, Salters K, et al. Exploring Primary Healthcare Experiences and Interest in Mobile Technology Engagement Amongst an Urban Population Experiencing Barriers to Care. *Qual Health Res.* 2023;33(8-9):765-77.

130. Marshall D, Quinn C, Child S, Shenton D, Pooler J, Forber S, et al. What IAPT services can learn from those who do not attend. *J Ment Health*. 2016;25(5):410-5.

131. Vetter I. *Primary health care for people with multiple and complex needs: what does best practice look like?* Fulfilling Lives/University of Brighton, 2020. https://www.bht.org.uk/wp-content/uploads/2021/02/Primary-Healthcare-What-does-best-practice-look-like-May-2020.pdf [accessed 30.04.2024]

132. Sun C-A, Taylor K, Levin S, Renda SM, Han H-R. Factors associated with missed appointments by adults with type 2 diabetes mellitus: a systematic review. *Diabetes Res Car*e 2021;9(1).

133. Lawal M, Woodman A. Socio-demographic determinants of attendance in diabetes education centres: a survey of patients’ views. *EMJ Diabetes*. 2021;9(1):102-9.

134. Nguyen DL, Dejesus RS, Wieland ML. Missed appointments in resident continuity clinic: patient characteristics and health care outcomes. *J Grad Med Educ.* 2011;3(3):350-5.

135. Abdulkadir LS, Mottelson IN, Nielsen DS. Why does the patient not show up? Clinical case studies in a Danish migrant health clinic. *Eur J Pers Cent Healthc*. 2019;7(2):316-24.

136. Sumarsono A, Case M, Kassa S, Moran B. Telehealth as a Tool to Improve Access and Reduce No-Show Rates in a Large Safety-Net Population in the USA. *J Urban Health.* 2023;100(2):398-407.

137. Tonnesen M, Hedeager Momsen A-M. Bridging gaps in health? A qualitative study about bridge-building and social inequity in Danish healthcare. *Int J Qual Stud Health Well-being.* 2023;18(1):2241235.

138. Revolving Doors Agency. *Navigating complexity: learning from Navigators across Birmingham.* Revolving Doors Agency, 2020. https://www.tnlcommunityfund.org.uk/media/insights/documents/Navigating-Complexity-Learning-from-the-navigators-across-Birmingham-2020.pdf?mtime=20220601114929&focal=none [accessed 30.04.2024]

139. Hickmott S, Stroud T. Hidden dimensions. The complexities of podiatry clinic non attendance of people with diabetes. *Diabet Med*. 2009;26(SUPPL. 1):174.

140. Martin C, Perfect T, Mantle G. Non-attendance in primary care: the views of patients and practices on its causes, impact and solutions. *Fam Pract*. 2005;22(6):638-43.

141. Link BG, Phelan JC. Conceptualizing stigma. *Annual Review of Sociology*. 2001;27(1):363-85.

142. Wilkinson MJ. Effecting change in frequent non-attenders. *Br J Gen Pract*. 1994;44(382):233.

143. Maggs C, Langley C. Why patients miss primary care appointments: involving patients in research. *Prim Health Care*. 2008;18(2):34-7.

144. Bull SL, Frost N, Bull ER. Behaviourally informed, patient-led interventions to reduce missed appointments in general practice: a 12-month implementation study. *Fam Pract*. 2022.

145. Ballantyne M, Liscumb L, Brandon E, Jaffar A, Macdonald A, Beaune L. Mothers' Perceived Barriers to and Recommendations for Health Care Appointment Keeping for Children Who Have Cerebral Palsy. *Glob Qual Nurs Res*. 2019;6.

146. Boshers EB, Cooley ME, Stahnke B. Examining no-show rates in a community health centre in the United States. *Health Soc Care Community.* 2022;30(5):e2041-e9.

147. Ruggeri K, Folke T, Benzerga A, Verra S, Buttner C, Steinbeck V, et al. Nudging New York: adaptive models and the limits of behavioral interventions to reduce no-shows and health inequalities. *BMC Health Serv Res*. 2020;20(1).

148. Department for Levelling Up. *Frontline support models for people experiencing multiple disadvantage: A Rapid Evidence Assessment.* London: HMSO. April 2023. https://assets.publishing.service.gov.uk/media/642af3507de82b000c31350c/Changing_Futures_Evaluation_-_Frontline_support_models_REA.pdf [accessed 30.04.2024]

149. Lawal MO. Non-attendance in diabetes education centres: Perceptions of patients and education providers. *Diabet Med*. 2014;31(SUPPL. 1):102-3.

150. Sharp DJ, Hamilton W. Non-attendance at general practices and outpatient clinics. *BMJ*. 2001;323(7321):1081-2.

151. Denneny EK, Black SE, Bogle Y, Macavei VM, O'Shaughnessy TC, White VLC, et al. Tackling poor attendance to tuberculosis clinic-who, why and what can be done. *Thorax*. 2014;69(SUPPL. 2):A210.

152. Mackenzie M, Conway E, Hastings A, Munro M, O’Donnell CA. Intersections and multiple ‘candidacies’: exploring connections between two theoretical perspectives on domestic abuse and their implications for practicing policy. *Soc Policy Soc*. 2015;14(1):43-62.

153. Gallacher K, Morrison D, Jani B, Macdonald S, May CR, Montori VM, et al. Uncovering treatment burden as a key concept for stroke care: a systematic review of qualitative research. *PLoS medicine*. 2013;10(6):e1001473.

154. Fairhurst K, Sheikh A. Texting appointment reminders to repeated non-attenders in primary care: randomised controlled study. *Qual Saf Health Care*. 2008;17(5):373-6.

155. Morris J, Campbell-Richards D, Wherton J, Sudra R, Vijayaraghavan S, Greenhalgh T, et al. Webcam consultations for diabetes: findings from four years of experience in Newham. *Pract Diabetes*. 2017;34(2):45-50.

156. Arber S, Sawyer L. Do Appointment Systems Work? *BMJ*. 1982;284(6314):478-80.

157. Qin J, Chan CW, Dong J, Homma S, Ye S. Telemedicine is associated with reduced socioeconomic disparities in outpatient clinic no-show rates of attendence. *Journal of Telemedicine and Telecare.* 2023:0(0)

158. Johnson BJ, Mold JW, Pontious JM. Reduction and management of no-shows by family medicine residency practice exemplars. *Annals Family Med*. 2007;5(6):534-9.

159. Car J, Sheikh A. Telephone consultations. *BMJ*. 2003;326(7396):966-9.

160. Parsons J, Abel G, Mounce LTA, Atherton H. The changing face of missed appointments. *Br J Gen Pract*. 2023;73(728):134-5.

161. Garuda SR, Javalgi RG, Talluri VS. Tackling no-show behavior: a market-driven approach. *Health Mark Q*. 1998;15(4):25-44.

162. Woodcock EW. Managing your appointment'no-shows'. *J Med Pract Manag*. 2000;15:284-8.

163. Kiruparan P, Kiruparan N, Debnath D. Impact of pre-appointment contact and short message service alerts in reducing 'Did Not Attend' (DNA) rate on rapid access new patient breast clinics: a DGH perspective. *BMC Health Serv Res.* 2020;20(1):9.

164. Ellis DA, Jenkins R. Weekday Affects Attendance Rate for Medical Appointments: Large-Scale Data Analysis and Implications. *PLoS One*. 2012;7(12):4.

165. NHS England. *Approaches to implementing two-way appointment reminders*​. NHS England, 2023.

166. Gurol-Urganci I, e Jongh T, Vodopivec-Jamsek V, Atun R, Car J. Mobile phone messaging reminders for attendance at healthcare appointments. *Cochrane Database Syst Rev*. 2013(12).

167. Opon SO, Tenambergen WM, Njoroge KM. The effect of patient reminders in reducing missed appointment in medical settings: a systematic review. *PAMJ-One Health*. 2020 2(9).

168. Boksmati N, Butler-Henderson K, Anderson K, Sahama T. The Effectiveness of SMS Reminders on Appointment Attendance: a Meta-Analysis. *J Med Syst*. 2016;40(4):10.

169. Schwebel FJ, Larimer ME. Using text message reminders in health care services: A narrative literature review. *Internet Interv*. 2018;13:82-104.

170. Sims H, Sanghara H, Hayes D, Wandiembe S, Finch M, Jakobsen H, et al. Text Message Reminders of Appointments: A Pilot Intervention at Four Community Mental Health Clinics in London. *Psychiatr Serv*. 2012;63(2):161-8.

171. Car J, Ng C, Atun R, Card A. SMS Text Message Healthcare Appointment Reminders in England. *J Ambul Care Manage*. 2008;31(3):216-9.

172. Fee PA, Hargan AM. An intervention study to assess the effectiveness of a reminder telephone call in improving patient appointment attendance at a Community Dental Service clinic. *Community Dent Health*. 2016;33(4):239-41.

173. Reekie D, Devlin H. Preventing failed appointments in general dental practice: a comparison of reminder methods. *Br Dent J*. 1998;185(9):472-4.

174. McLean SM, Booth A, Gee M, Salway S, Cobb M, Bhanbhro S, et al. Appointment reminder systems are effective but not optimal: results of a systematic review and evidence synthesis employing realist principles*. Patient Prefer Adherence.* 2016;10:479-99.

175. Milne RG. Reducing non‐attendance at specialist clinics: an evaluation of the effectiveness and cost of patient‐focussed booking and SMS reminders at a Scottish health board.

*Int J Consum Stud.* 2010;34(5):570-80.

176. Wang Y, Baidoo FA. Design of Integral Reminder for Collaborative Appointment Management. *50th Annual Hawaii International Conference on System Sciences(HICSS)*. 2017:910-9.

177. Shah SJ, Cronin P, Hong CS, Hwang AS, Ashburner JM, Bearnot BI, et al. Targeted Reminder Phone Calls to Patients at High Risk of No-Show for Primary Care Appointment: A Randomized Trial*. J Gen Intern Med*. 2016;31(12):1460-6.

178. Perron NJ, Dao MD, Kossovsky MP, Miserez V, Chuard C, Calmy A, et al. Reduction of missed appointments at an urban primary care clinic: a randomised controlled study. *BMC* *Fam Pract*. 2010;11.

179. Macharia WM. An overview of interventions to improve compliance with appointment keeping for medical services. *JAMA*. 1992;267(13):1813-7.

180. Rowett M, Reda S, Makhoul S. Prompts to Encourage Appointment Attendance for People With Serious Mental Illness. *Schizophr Bull.* 2010;36(5):910-1.

181. Yates L, Brittleton L, Bean N. An investigation into the factors which influence attendance rates for psychology appointments in an adult intellectual disability service. *Adv Ment Health Intellet Disabil*. 2022;16(4):216-25.

182. Guo J, F. Bard J, J. Morrice D, R. Jaen C, Poursani R. Offering transportation services to economically disadvantaged patients at a family health center: a case study. *Health Systems.* 2022;11(4):251-75.

183. Healthwatch. *Cost of living: People are increasingly avoiding NHS appointments and prescriptions.* Healthwatch. 2023. https://www.healthwatch.co.uk/news/2023-01-09/cost-living-people-are-increasingly-avoiding-nhs-appointments-and-prescriptions [accessed 30.04.2024]

184. Sun C-A, Shenk Z, Renda S, Maruthur N, Zheng S, Perrin N, et al. Experiences and Perceptions of Telehealth Visits in Diabetes Care During and After the COVID-19 Pandemic Among Adults With Type 2 Diabetes and Their Providers: Qualitative Study. *JMIR Diabetes*. 2023;8:e44283-e.

185. Tait J, Noyes K, Bath L, Henderson M, Elleri D. Clinic non-attendance, glycaemic control and deprivation score in paediatric and young persons' diabetes clinics in Lothian, Scotland. *Pediatr Diabetes*. 2017;18(Supplement 25):88.

186. Weltermann BM, Doost SM, Kersting C, Gesenhues S. Hypertension management in primary care: how effective is a telephone recall for patients with low appointment adherence in a practice setting? *Wien Klin Wochenschr*. 2014;126(19-20):613-8.

187. Maehl N, Bleckwenn M, Riedel-Heller SG, Mehlhorn S, Lippmann S, Deutsch T, et al. The Impact of the COVID-19 Pandemic on Avoidance of Health Care, Symptom Severity, and Mental Well-Being in Patients With Coronary Artery Disease. *Front Med (Lausanne).* 2021;8.
